# Supplementary material for: Code Status Discussions: A Standardized Patient Workshop for Senior Medical Students
Source: MedEdPORTAL. 2025 Sep 2;21:11546. doi: 10.15766/mep_2374-8265.11546 (PMC12402213; doi:10.15766/mep_2374-8265.11546)
Supplement: Supplementary file 1 — Didactic.pptxStudent Case Handouts.docxFacilitator Guide.docxWorkshop Frameworks Handouts.docxPre- and Postworkshop Survey.docxSP Guide.docx [file mep_2374-8265.11546-s001.zip › D. Workshop Frameworks Handouts.docx]

*for those with advanced illness*

# ESTABLISH THE SETTING.

- 1. Sit at eye level.
  2. Ask if patient wishes for others to be present.
  3. Introduce the subject and ask for permission to proceed:

i. admit to the


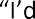

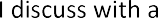

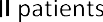

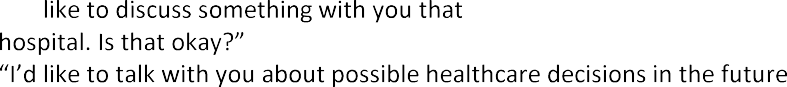


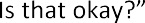
ii.

# WHAT DOES THE PATIENT OR CAREGIVER UNDERSTAND?

- 1.
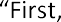

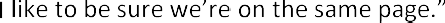

  2. Ask open-ended questions:

i.


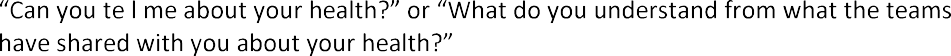


- 1. If the patient is open to it, fill in any details that may be missing: i.
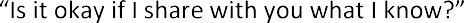


# WHAT IS IMPORTANT TO THEM IN THE CONTEXT OF THEIR ILLNESS?


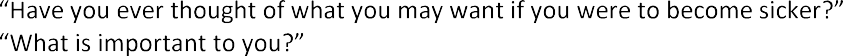
a.

b.

# DISCUSS CODE STATUS.

- 1. Introduce the topic and provide a basic definition. i.


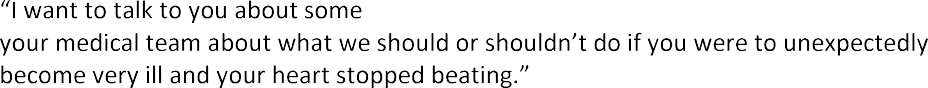


thing called code status. This provides directions to

- 1. Ask what they know or may like to know about code status and explain in simple terms.

i.
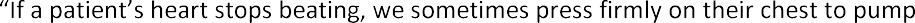


blood through their body, use electricity to shock their heart back into beating, and


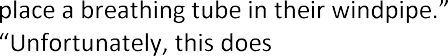

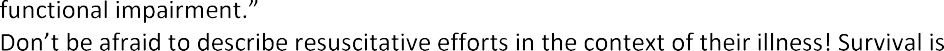
ii. not always lead to recovery or survival. In fact, only about 1 in 7 people survive CPR and are discharged from the hospital. Some of these may experience complications related to CPR, including permanent neurological and

iii. iv.

decreased with advanced illness.


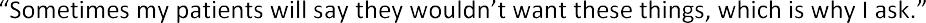


# ESTABLISH A PLAN THAT ALIGNS WITH THE PAT
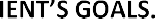


- 1. Feel free to make a recommendation with their permission.

# SUMMARIZE YOUR DISCUSSION.

a.


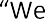

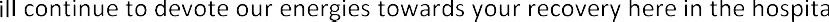

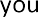


heart were to unexpectedly stop or your breathing worsen, instead of performing chest compressions or placing you on a breathing machine, we would make you comfortable at the


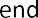

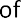

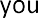

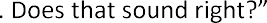


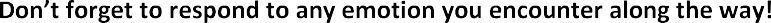


# This can be a difficult topic for patients and families!

*for those without advanced illness*

# NORMALIZE AND CONTEXTUALIZE THE CONVERSATION.

- 1.
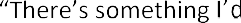


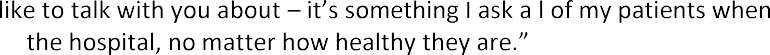


they are first admitted to

# INTRODUCE CODE STATUS AND ASK WHAT THEY KNOW.

a.


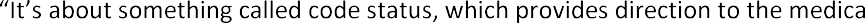

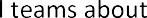


what patients may or may not want done should their heart stop beating or should they stop b.
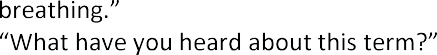


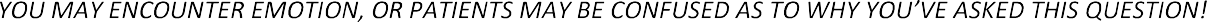


# EMPATHIZE AND REASSURE.


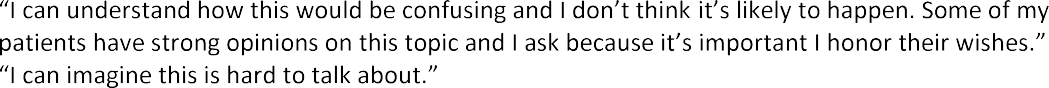
a.

b.

# PROVIDE EDUCATION IF NEEDED AND USE SIMPLE TERMS.

a.
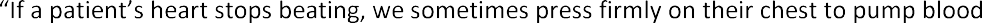


through their body, use electricity to shock their heart back into beating, and place a breathing


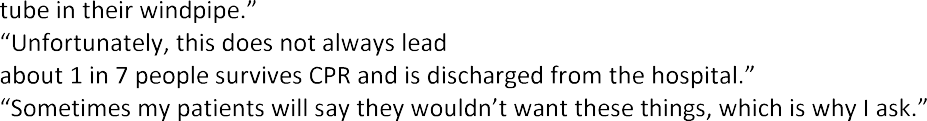
b. to recovery or survival like we see on TV. In fact, only

c.

# LISTEN FOR THEIR RESPONSE.

a.
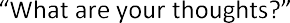


# SUMMARIZE THE PLAN.

a.


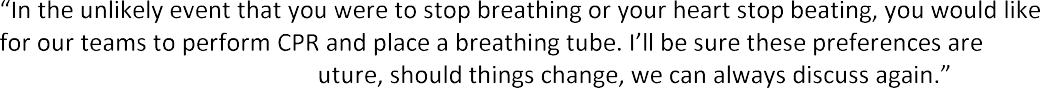


reflected in the chart. In the f
