# Supplementary material for: Code Status Discussions: A Standardized Patient Workshop for Senior Medical Students
Source: MedEdPORTAL. 2025 Sep 2;21:11546. doi: 10.15766/mep_2374-8265.11546 (PMC12402213; doi:10.15766/mep_2374-8265.11546)
Supplement: Supplementary file 1 — Didactic.pptxStudent Case Handouts.docxFacilitator Guide.docxWorkshop Frameworks Handouts.docxPre- and Postworkshop Survey.docxSP Guide.docx [file mep_2374-8265.11546-s001.zip › E. Pre- and Postworkshop Survey.docx]

| *I am confident I can…* | | | | | |
| --- | --- | --- | --- | --- | --- |
|  | Not confident at all | Somewhat confident | Moderately confident | Very confident | Extremely confident |
| 1. Recognize how a patient's prognosis impacts how I lead the code status conversation. | 🞎 | 🞎 | 🞎 | 🞎 | 🞎 |
| 1. Recognize the appropriate timing for when to address code status. | 🞎 | 🞎 | 🞎 | 🞎 | 🞎 |
| 1. Explain the phrase "code status" to a patient in easy-to-understand language. | 🞎 | 🞎 | 🞎 | 🞎 | 🞎 |
| 1. Propose a "code status" that aligns with the patient's values. | 🞎 | 🞎 | 🞎 | 🞎 | 🞎 |
| 1. Elicit values from the patient that help me guide code status discussion. | 🞎 | 🞎 | 🞎 | 🞎 | 🞎 |

| *Select how appropriate you feel each phrase is when discussing code status with a patient or their family:* | | | | | | | |
| --- | --- | --- | --- | --- | --- | --- | --- |
|  | Always  inappropriate | | Usually inappropriate | | Sometimes appropriate | Usually appropriate | Always appropriate |
| 1. “Every hospitalized patient needs to be DNR when actively dying.” | 🞎 | 🞎 | | 🞎 | | 🞎 | 🞎 |
| 1. “Only about 1 in 7 people survive CPR and are discharged from the hospital.” | 🞎 | 🞎 | | 🞎 | | 🞎 | 🞎 |
| 1. “Let’s re-discuss your wishes for end-of-life care.” | 🞎 | 🞎 | | 🞎 | | 🞎 | 🞎 |

| *I am confident I can…* | | | | | |
| --- | --- | --- | --- | --- | --- |
|  | Not confident at all | Somewhat confident | Moderately confident | Very confident | Extremely confident |
| 1. Recognize how a patient's prognosis impacts how I lead the code status conversation. | 🞎 | 🞎 | 🞎 | 🞎 | 🞎 |
| 1. Recognize the appropriate timing for when to address code status. | 🞎 | 🞎 | 🞎 | 🞎 | 🞎 |
| 1. Explain the phrase "code status" in health-care literate appropriate language. | 🞎 | 🞎 | 🞎 | 🞎 | 🞎 |
| 1. Propose a "code status" that aligns with the patient's values. | 🞎 | 🞎 | 🞎 | 🞎 | 🞎 |
| 1. Elicit values from the patient that help me guide code status discussion. | 🞎 | 🞎 | 🞎 | 🞎 | 🞎 |

| *Select how appropriate you feel each phrase is when discussing code status with a patient or their family:* | | | | | | | |
| --- | --- | --- | --- | --- | --- | --- | --- |
|  | Always  inappropriate | | Usually inappropriate | | Sometimes appropriate | Usually appropriate | Always appropriate |
| 1. “Every hospitalized patient needs to be DNR when actively dying.” | 🞎 | 🞎 | | 🞎 | | 🞎 | 🞎 |
| 1. “Only about 1 in 7 people survive CPR and are discharged from the hospital.” | 🞎 | 🞎 | | 🞎 | | 🞎 | 🞎 |
| 1. “Let’s re-discuss your wishes for end-of-life care.” | 🞎 | 🞎 | | 🞎 | | 🞎 | 🞎 |
