# Supplementary material for: Code Status Discussions: A Standardized Patient Workshop for Senior Medical Students
Source: MedEdPORTAL. 2025 Sep 2;21:11546. doi: 10.15766/mep_2374-8265.11546 (PMC12402213; doi:10.15766/mep_2374-8265.11546)
Supplement: Supplementary file 1 — Didactic.pptxStudent Case Handouts.docxFacilitator Guide.docxWorkshop Frameworks Handouts.docxPre- and Postworkshop Survey.docxSP Guide.docx [file mep_2374-8265.11546-s001.zip › F. SP Guide.docx]

**Medicine Scenario #1: Discussing Code Status on Admission**

Intern:

You are admitting Mr. / Ms. Reed, a 63yo with RUQ pain, fever, and leukocytosis, found to have acute cholecystitis. They are being admitted for IV fluids, pain control, and antibiotics – your upper level has finished placing the orders. The patient is now afebrile and appears comfortable. You wish to discuss code status.

Standardized Patient:

- You are the patient.
- You are not familiar with the term “code status” and ask the intern to explain what it is.
- You become alarmed as they provide the explanation, worried this means they expect you to die in the hospital.
- You ask what CPR looks like and what your chances are of survival.
- You are young and otherwise healthy – you would like chest compressions or to be placed on a ventilator if needed.

**Medicine Scenario #2: Discussing Code Status in the Clinic**

Intern:

You are seeing one of your primary care clinic patients, Mr. / Ms. Smith, for follow-up. During their last visit 2 weeks ago, you reviewed the unfortunate news they received from oncology that their metastatic melanoma has progressed through the most recent line of treatment and unfortunately, there are no more cancer-directed treatment options available. You scheduled this visit today as a general check-in. Now that Mr. / Ms. Smith has had time to process this news, your attending asks you to discuss code status with them.

Standardized Patient:

- You are the patient.
- You are saddened by the news that your cancer has progressed but are agreeable to talking about code status and what you may want if you were to become sicker.
- The most important things to you are having more quality time with your family and being comfortable.
- You have decided that you don’t want to pursue any further cancer-directed treatments (chemo, radiation, surgery)
- You are ok with being admitted to the hospital for a short time to treat a reversible illness or if you need inpatient treatments to stay comfortable, but you don’t want to ever be admitted to the ICU.
- You ask the intern to make a recommendation about what you should do as it pertains to resuscitation.
- If the student asks about being put on a ventilator, life support, or getting artificial nutrition (tube feeds), you can share that those are not things you would ever want.

**Medicine Scenario #3 – Discussing Code Status with Clinical Worsening while Inpatient**

Intern:

You are the intern in the ICU caring for Mr./Mrs. Baker, who is a 38yo with a history of substance use disorder admitted after an unintentional heroin overdose, at which time they suffered a cardiac arrest. They have unfortunately remained minimally responsive without sedation and now, on day 9 of admission, have developed a ventilator-associated pneumonia. Throughout their course, you have spoken with their spouse about what Mr./Ms. Baker values — their sense of humor, being active, time with family, and their independence. They are now in septic shock and requiring three pressors. Your team met with their spouse earlier in the day and delivered the unfortunate news that given the severity of their infection now with multi-organ failure, you worry Mr./Mrs. Baker is going to die. After giving their spouse some time to process this, your upper level asks you to address code status.

Standardized Patient:

- You are the spouse of the patient.
- You can’t believe that you are in this spot and ask, “Why is this happening?”
- After the intern responds empathically to this question, you calm down and say you are willing to discuss next steps.
- When the intern discusses code status with you, you ask for more information about what this means.
- When the intern asks whether you think your spouse would want chest compressions should his heart stop, you say that you want everything done that can help him to stay alive.
- If the intern continues to push, you explain that as his spouse, you can’t make the decision to stop doing aggressive things. You feel that it’s your job to fight for your husband and couldn’t go on knowing that you didn’t do everything you could for him.

**Peds Scenario #1: Discussing Code Status in the NICU**

Intern:

You are caring for Jade, a 10-day-old full term baby girl with a skeletal dysplasia. As part of her condition, she has a small thorax with abnormal ribs. Her family was prepared that she would very likely require respiratory support and were accepting of intubation if needed. She is now on the ventilator and clinically stable, though you know her hospitalization is likely to be prolonged and perhaps complicated. You have come to know her family over the past few days and after providing medical updates, wish to discuss code status.

Standardized Patient:

- You are the parent of Jade.
- You’re not sure what the term “code status” means and ask the intern to explain.
- As they do this, you become concerned saying you thought Jade’s condition had stabilized and are wondering if this means the medical team expects her to get sicker.
- After the intern responds to this emotion and concern, you calm down.
- You are fine with Jade being on a ventilator, but you worry that if her heart stops, doing chest compressions would cause her a lot of pain given how small her chest is.
- You decide you wouldn’t want Jade to have chest compressions, but would rather let her die peacefully if her heart stops.

**Peds Scenario #2: Discussing Code Status in the Clinic**

Intern:

You are rotating through the neurology clinic and are now seeing TJ, an 18-month-old with SMA type 1. SMA is *spinal muscle atrophy*, a neuromuscular disorder characterized by progressive muscle weakness, type 1 being most severe and typically associated with death by 2 years of age due to respiratory failure. TJ is diffusely weak, minimally able to move his body. His parents have noticed his cough is weak and he has begun to lose weight, unable to take sufficient oral nutrition. Given his progressive decline, your attending wishes for you to discuss what parents may prefer for TJ’s medical treatment in the future. They specifically ask that you discuss code status.

Standardized Patient:

- You are the parent of TJ.
- You know that TJ’s neuromuscular condition is incurable and likely to lead to death in a short time.
- You have worried over the past few months that TJ has been having more bad days than good.
- You don’t want TJ to suffer and worry this is beginning to happen.
- Based on this, you ask the intern to make a recommendation regarding code status.
- You agree with their recommendation that the teams focus on keeping TJ comfortable as his body continues to decline – should his heart stop beating or his respiratory status worsen, you would prefer for him to be made comfortable at the end of his life.

**Peds Scenario #3: Discussing Code Status with Clinical Decline While in the PICU**

Intern:

You are the intern caring for Luke, a 15yo M with history of refractory AML admitted with complications related to bone marrow transplant. He has been in the hospital for 2 months and is now bacteremic with septic shock. He is intubated and is requiring maximal pressor support. Throughout his course, you have spoken with his parents about what makes Luke himself – his sense of humor, his love of family, his independence – all things he values deeply. You meet with his parents and deliver the unfortunate news that given the severity of his infection, you worry Luke will die. After giving them some time to process this news, your upper level asks you to return to discuss code status.

Standardized Patient:

- You are Luke’s parent.
- You can’t believe you are in this situation and thought Luke would get better.
- After the intern responds to your emotion, you begin to calm down.
- You ask for more details about what code status means and as the intern responds, you become scared and say you don’t want to feel like you are “giving up” on him.
- You feel that as his parent, it’s your job to fight for Luke, and you need to know that you did everything you could for him. You want the doctors to do everything they possibly can to keep him alive, including chest compressions if his heart stops.

**MP/FM Scenario #1: Discussing Code Status on Admission**

Intern:

You are admitting Mr. / Ms. Reed, a 63yo with RUQ pain, fever, and leukocytosis, found to have acute cholecystitis. They are being admitted for IV fluids, pain control, and antibiotics – your upper level has finished placing their orders. They are now afebrile and appear comfortable. You wish to discuss code status.

Standardized Patient:

- You are the patient.
- You are not familiar with the term “code status” and ask the intern to explain what it is.
- You become alarmed as they provide the explanation, worried this means they expect you to die in the hospital.
- You ask what CPR looks like and what your chances are of survival.
- You are young and otherwise healthy – you would like chest compressions or to be placed on a ventilator if needed.

**MP/FM Scenario #2: Discussing Code Status in the Clinic**

Intern:

You are rotating through the neurology clinic and are now seeing TJ, an 18-month-old with SMA type 1. SMA is *spinal muscle atrophy*, a neuromuscular disorder characterized by progressive muscle weakness, type 1 being most severe and typically associated with death by 2 years of age due to respiratory failure. TJ is diffusely weak, minimally able to move his body. His parents have noticed his cough is weak and he has begun to lose weight, unable to take sufficient oral nutrition. Given his progressive decline, your attending wishes for you to discuss what parents may prefer for TJ’s medical treatment in the future. They specifically ask that you discuss code status with his parent.

Standardized Patient:

- You are the parent of TJ.
- You know that TJ’s neuromuscular condition is incurable and likely to lead to death in a short time.
- You have worried over the past few months that TJ has been having more bad days than good.
- You don’t want TJ to suffer and worry this is beginning to happen.
- Based on this, you ask the intern to make a recommendation regarding code status.
- You agree with their recommendation that the teams focus on keeping TJ comfortable as his body continues to decline – should his heart stop beating or his respiratory status worsen, you would prefer for him to be made comfortable at the end of his life.

**MP/FM Scenario #3: Discussing Code Status with Clinical Decline While in the PICU**

Intern:

You are the intern caring for Luke, a 15yo M with history of refractory AML admitted with complications related to bone marrow transplant. He has been in the hospital for 2 months and is now bacteremic with septic shock. He is intubated and is requiring maximal pressor support. Throughout his course, you have spoken with his parents about what makes Luke himself – his sense of humor, his love of family, his independence – all things he values deeply. You meet with his parents and deliver the unfortunate news that given the severity of his infection, you worry Luke will die. After giving them some time to process this news, your upper level asks you to return to discuss code status.

Standardized Patient:

- You are Luke’s parent.
- You can’t believe you are in this situation and thought Luke would get better.
- After the intern responds to your emotion, you begin to calm down.
- You ask for more details about what code status means and as the intern responds, you become scared and say you don’t want to feel like you are “giving up” on him.
- You feel that as his parent, it’s your job to fight for Luke, and you need to know that you did everything you could for him. You want the doctors to do everything they possibly can to keep him alive, including chest compressions if his heart stops.

**OB/GYN Scenario #1: Discussing Code Status on Admission**

Intern

You are admitting Ms. Reed, a nulliparous 63-year-old presenting with abnormal uterine bleeding. She is being admitted for overnight observation after receiving 1 unit packed red blood cell transfusion for a hemoglobin of 6.8. She has a known history of iron deficiency anemia with a baseline hemoglobin of 7.5, and she has been hemodynamically stable since arrival to the ER today. Your upper level is placing her orders and you are tasked with discussing her code status.

Standardized Patient:

- You are the patient.
- You are not familiar with the term “code status” and ask the intern to explain what it is.
- You become alarmed as they provide the explanation, worried this means they expect you to die in the hospital.
- You ask what CPR looks like and what your chances are of survival.
- You are young and otherwise healthy – you would like chest compressions or to be placed on a ventilator if needed.

**OB/GYN Scenario #2: Discussing Code Status with Worsening Prognosis**

Intern:

You are admitting Ms. Root, a 58 year-old with known stage III ovarian cancer presenting to the hospital from the GYN/ONC clinic with failure to thrive. She was diagnosed 4 years ago and has received several rounds of chemotherapy. She initially responded well to therapy but her latest CMP revealed markedly elevated ALP and transaminases concerning for extension of her disease into the liver. CT in the ED confirmed new liver metastases. You are admitting her to the GYN/ONC inpatient service and now will discuss her code status. She has previously been full code.

Standardized Patient:

- You are the patient.
- You are saddened by the news that your cancer has progressed but are agreeable to talking about code status and what you may want if you were to become sicker.
- The most important things to you are having more quality time with your family and being comfortable.
- You have decided that you don’t want to pursue any further cancer-directed treatments (chemo, radiation, surgery)
- You are ok with being admitted to the hospital for a short time to treat a reversible illness or if you need inpatient treatments to stay comfortable, but you don’t want to ever be admitted to the ICU.
- You ask the intern to make a recommendation about what you should do as it pertains to resuscitation.
- If the student asks about being put on a ventilator, life support, or getting artificial nutrition (tube feeds), you can share that those are not things you would ever want.

**OB/GYN Scenario #3: Discussing Code Status with Clinical Worsening while Inpatient**

Intern:

You are the intern caring for Ms. Road, a 70 yo F who presented with a saddle pulmonary embolism (PE) and was found to have a new diagnosis of high-grade endometrial carcinoma. Her PE was complicated by cardiac arrest for which she received chest compressions, was intubated, and required management in the cardiac ICU. After extubation, she was transferred to the GYN/ONC team for further work-up of her abnormal uterine bleeding. Biopsy revealed high grade endometrial carcinoma and she was not offered surgery due to the extent of her disease. She continues to have vaginal bleeding that requires frequent blood transfusions and, unfortunately, her kidneys are beginning to fail. Due to her organ dysfunction and poor performance status, she is not currently a candidate for chemotherapy, but is receiving palliative radiation to control bleeding. She has been disturbed by the recent events, but believes she can recover if she receives chemotherapy. Your team is worried that she may soon require ICU-level care and that she is at high risk for having another cardiac arrest. Your upper level asks you to address code status.

Standardized Patient:

- - You are the patient.
  - You can’t believe that you are in this spot and ask, “Why is this happening?”
  - After the intern responds empathetically to this question, you calm down and say you are willing to discuss next steps.
  - When the intern discusses code status with you, you ask for more information about what this means.
  - When the intern asks whether you want chest compressions should your heart stop, you say that you want everything done to stay alive.
- You previously survived your heart stopping – why would you not survive this time?
- You want everything done so your body can continue to get stronger and you can get chemotherapy.
  - If the intern continues to push, you explain that you want everything done to buy more time to be with family.

**Surgery Scenario #1: Discussing Code Status on Hospital Admission**

Intern:

You are admitting Mr. / Ms. Reed, a 63yo with RUQ pain, fever, and leukocytosis, found to have acute cholecystitis. They are being admitted for IV fluids, pain control, and antibiotics – your upper level has finished placing the orders. The patient is now afebrile and appears comfortable. You wish to discuss code status.

Standardized Patient:

- You are the patient.
- You are not familiar with the term “code status” and ask the intern to explain what it is.
- You become alarmed as they provide the explanation, worried this means they expect you to die in the hospital.
- You ask what CPR looks like and what your chances are of survival.
- You are young and otherwise healthy – you would like chest compressions or to be placed on a ventilator if needed.

**Surgery Scenario #2: Discussing Code Status Prior to Surgery**

Intern:

Mr./Ms. Smith is an 85yo with history of hypertension admitted now with left hip fracture after sustaining a fall at their assisted living facility. Their spouse passed away several years ago and, due to mounting debility, Mr./Mrs. Smith previously made the decision to be DNR/DNI, stating “when it’s my time to go, I want to go”. Due to pain, they are wishing to undergo fracture repair. Your upper level asks that you discuss with Mr./Ms. Smith’s code status in the context of their upcoming surgery.

Standardized Patient:

- You are the patient
- As the intern brings up code status, you say you are familiar with the term and have previously made the decision to be DNR.
- Your spouse died 6 years ago, and you’d like to go when it’s your time and avoid any painful interventions
- You wonder why this may need to be changed in the setting of a surgery
- After the intern explains to you the risks associated with anesthesia and potential interventions that may need to be performed intraoperatively, you agree to temporarily change your code status to Full Code.
- You wish to re-visit this discussion after your surgery.

**Surgery Scenario #3: Clinical Worsening While in the SICU**

Intern:

Mr./Ms. Lee is a 65yo with a history of T2DM admitted two weeks ago with urosepsis and profound hypotension. Hemodynamics have improved, but they remain intubated due to weakness. Three days ago, they developed abdominal distension with intolerance of NG tube feeds. CT of the abdomen demonstrated bowel wall edema. They were taken to the OR where they were found to have bowel necrosis and underwent small bowel resection. They returned to the SICU with open abdomen. Unfortunately, over the past 24 hours, their clinical condition has deteriorated. Re-look of the abdomen reveals extensive necrosis of the remaining bowel not amenable to surgical intervention. You meet with the spouse and deliver the unfortunate news that given the severity of Mr./Ms. L’s illness, you believe they will not survive. After giving their spouse some time to process this news, you wish to discuss code status.

Standardized Patient:

- You are the spouse of the patient.
- You can’t believe that you are in this spot and ask, “Why is this happening?”
- After the intern responds empathically to this question, you calm down and say you are willing to discuss next steps.
- When the intern discusses code status with you, you ask for more information about what this means.
- When the intern asks whether you think your spouse would want chest compressions should their heart stop, you say that you want everything done that can help them stay alive. You can’t believe this is happening and aren’t sure what to think.
- If the intern continues to push, you explain that as the spouse, you can’t make the decision to stop doing aggressive things. You feel that it’s your job to fight for your spouse and couldn’t go on knowing that you didn’t do everything you could.
