# Supplementary material for: Code Status Discussions: A Standardized Patient Workshop for Senior Medical Students
Source: MedEdPORTAL. 2025 Sep 2;21:11546. doi: 10.15766/mep_2374-8265.11546 (PMC12402213; doi:10.15766/mep_2374-8265.11546)
Supplement: Supplementary file 1 — Didactic.pptxStudent Case Handouts.docxFacilitator Guide.docxWorkshop Frameworks Handouts.docxPre- and Postworkshop Survey.docxSP Guide.docx [file mep_2374-8265.11546-s001.zip › A. Didactic.pptx]

## Slide 1
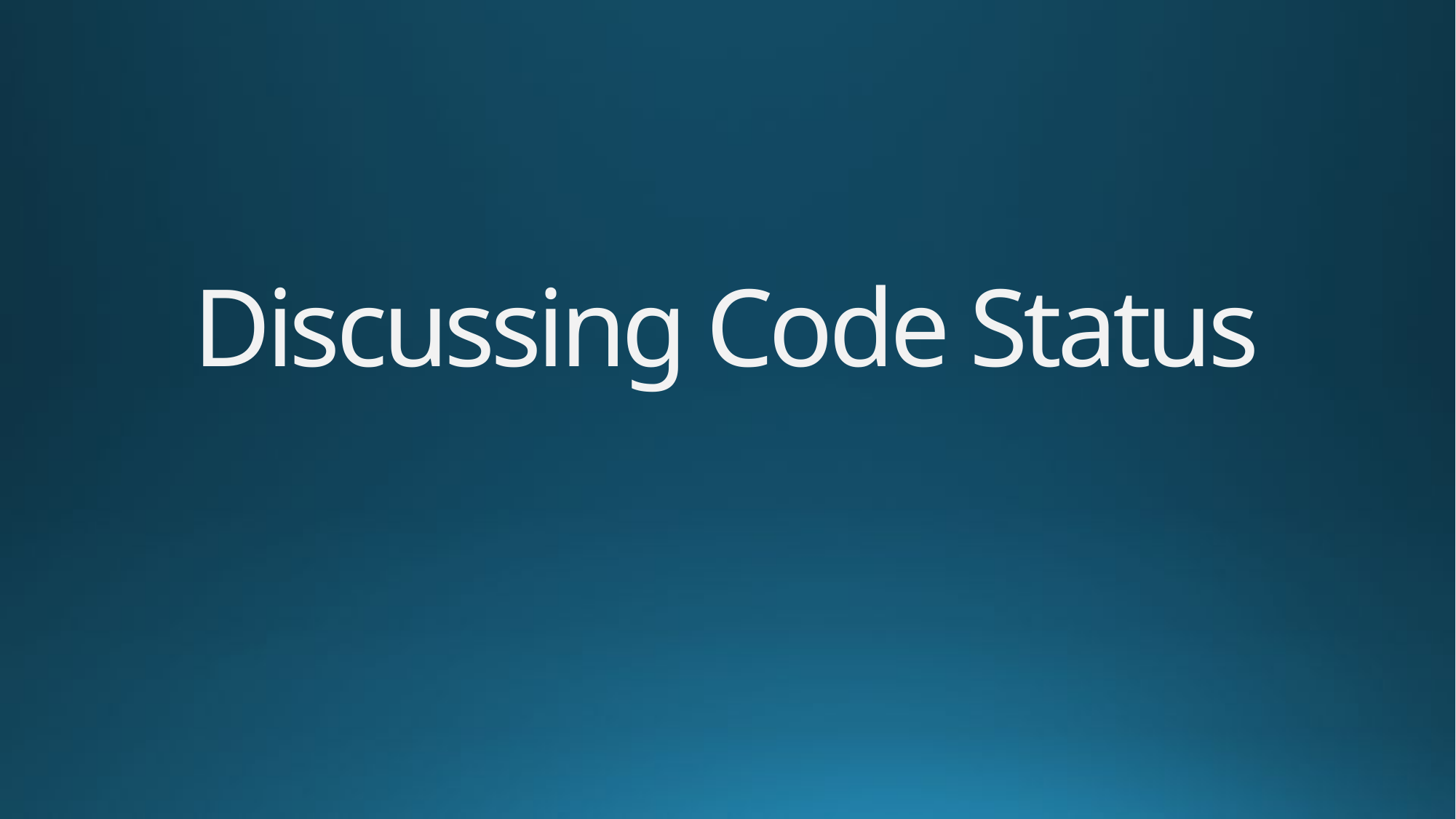

# Discussing Code Status

## Slide 2
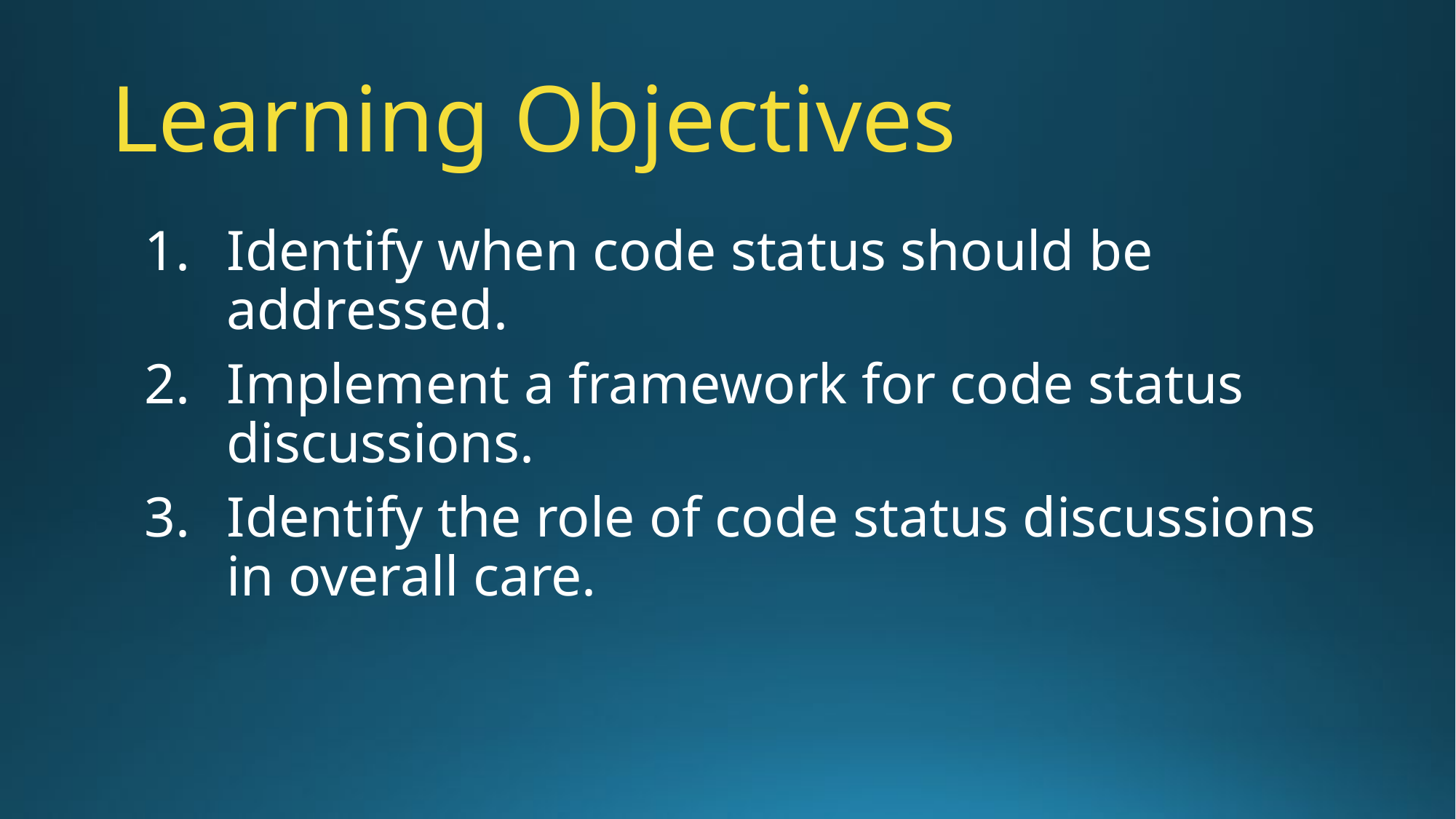

# Learning Objectives
Identify when code status should be addressed.
Implement a framework for code status discussions.
Identify the role of code status discussions in overall care.

## Slide 3
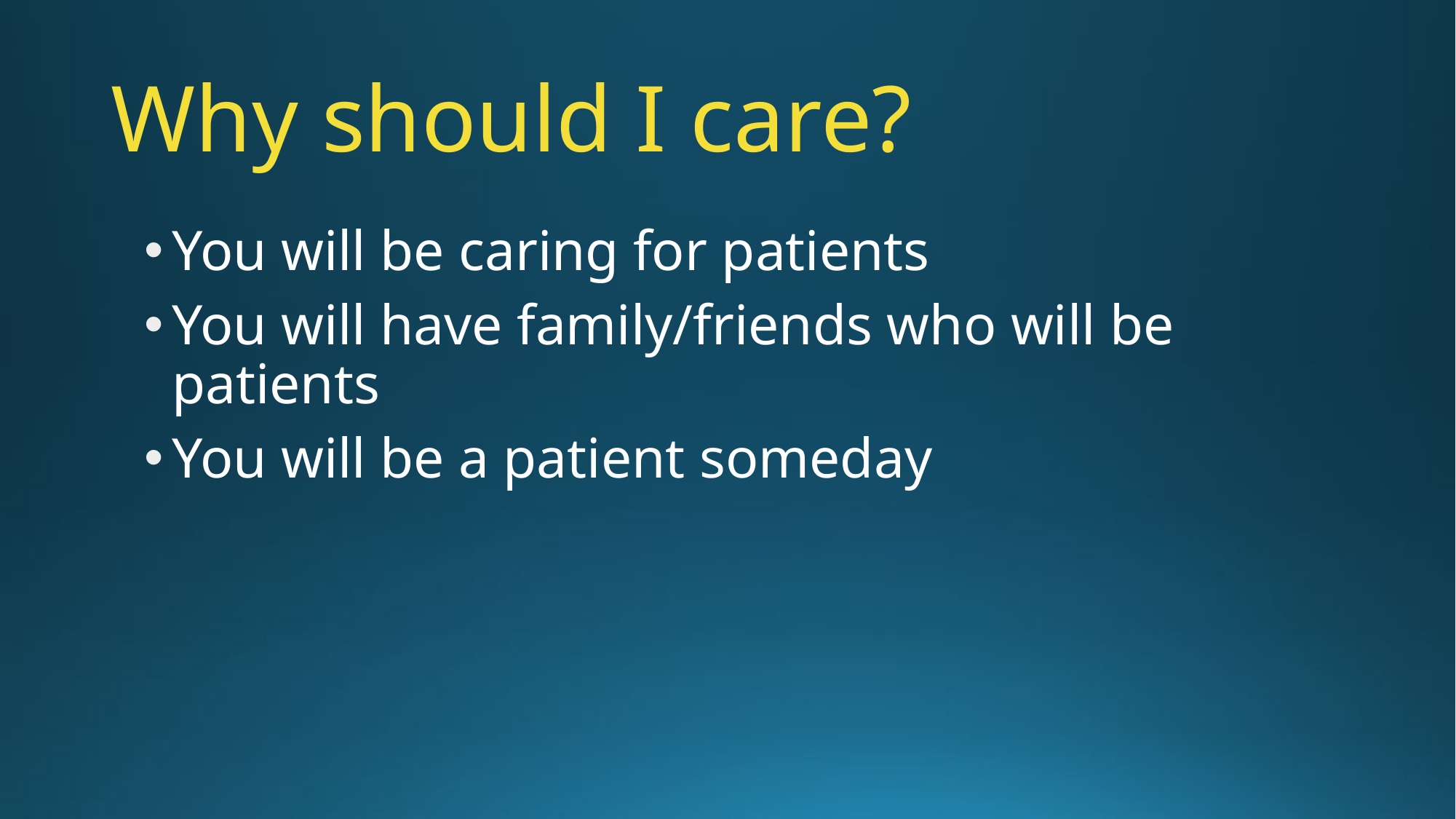

# Why should I care?
You will be caring for patients
You will have family/friends who will be patients
You will be a patient someday

## Slide 4
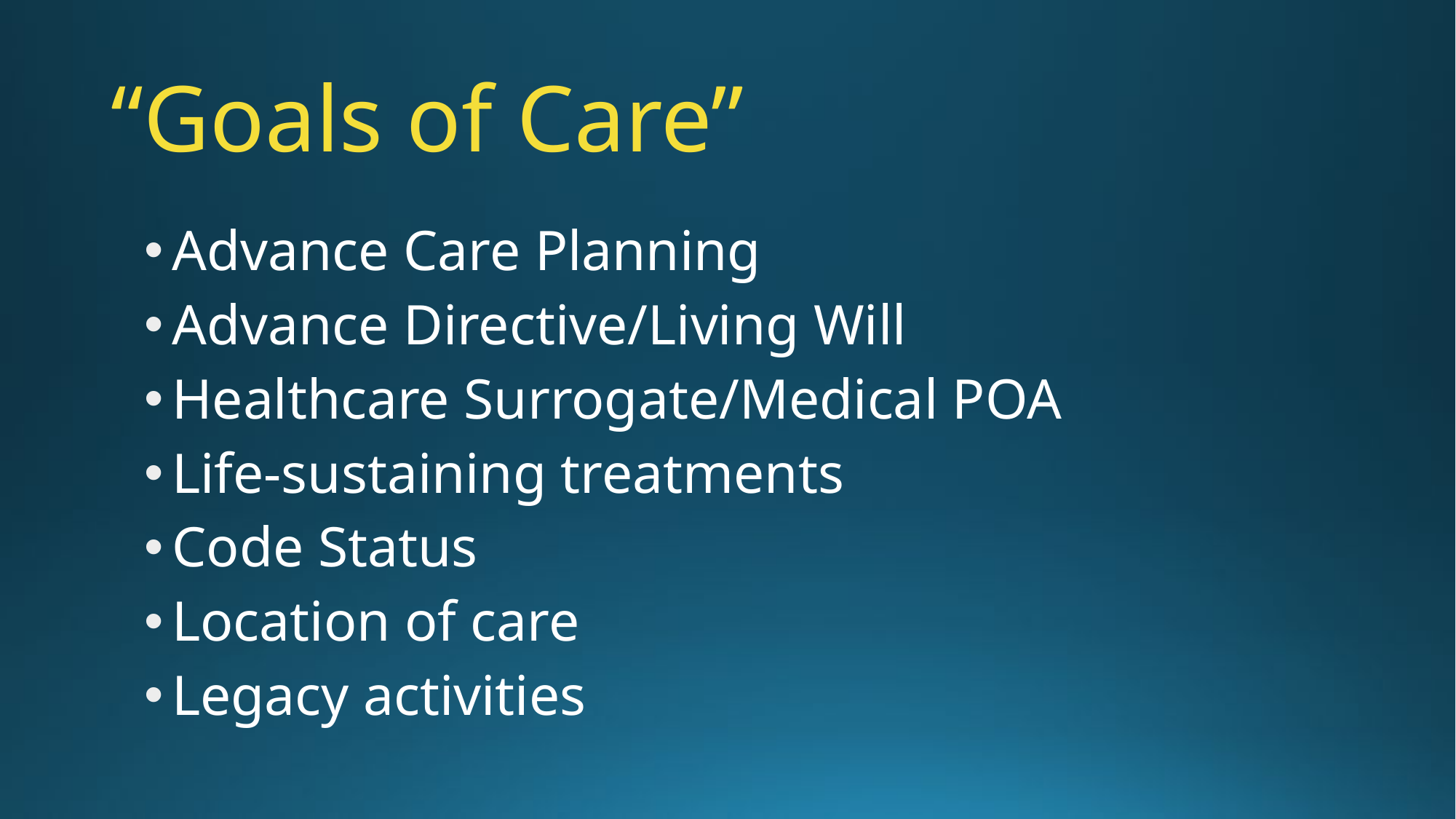

# “Goals of Care”
Advance Care Planning
Advance Directive/Living Will
Healthcare Surrogate/Medical POA
Life-sustaining treatments
Code Status
Location of care
Legacy activities

## Slide 5
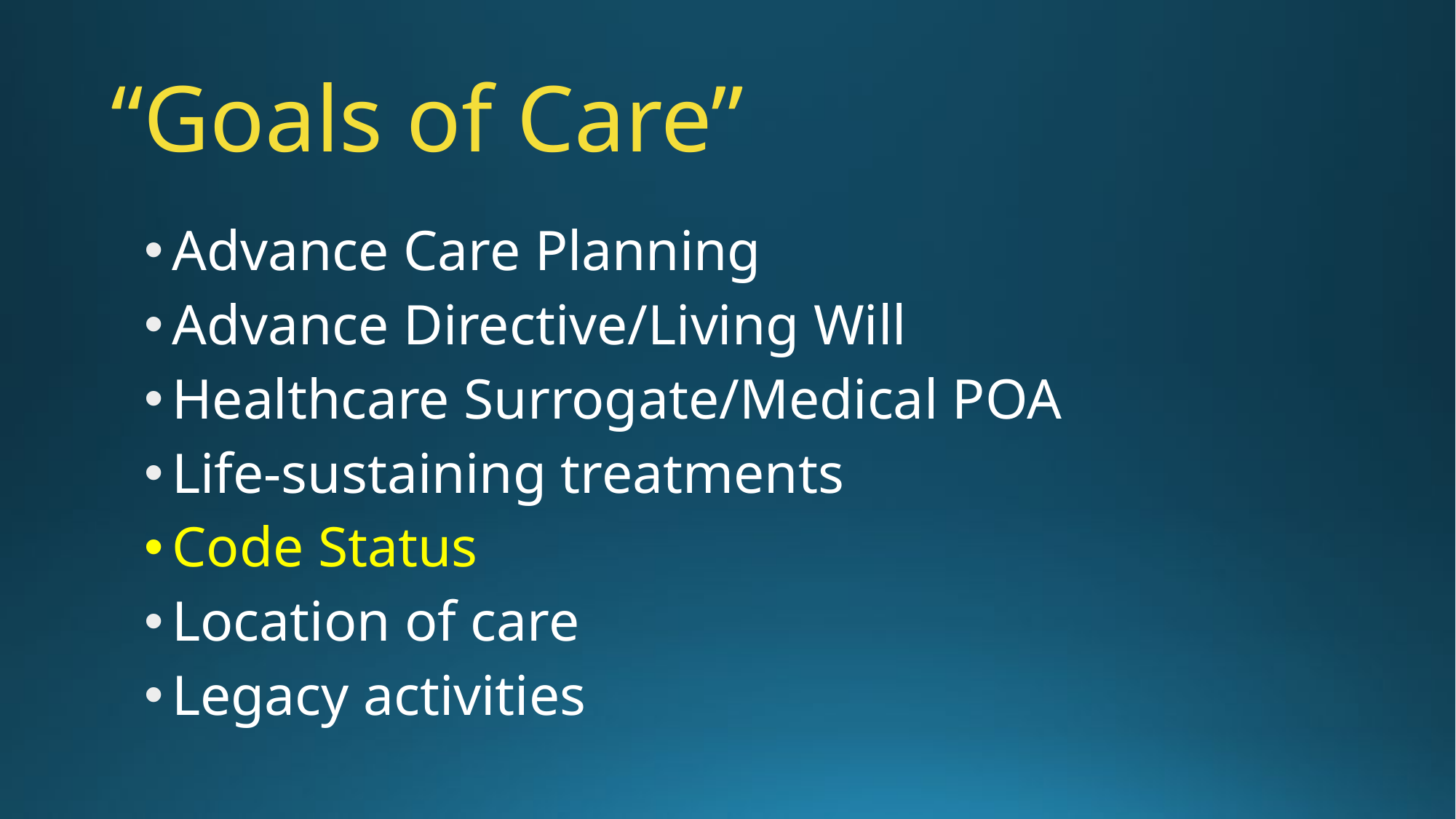

# “Goals of Care”
Advance Care Planning
Advance Directive/Living Will
Healthcare Surrogate/Medical POA
Life-sustaining treatments
Code Status
Location of care
Legacy activities

## Slide 6
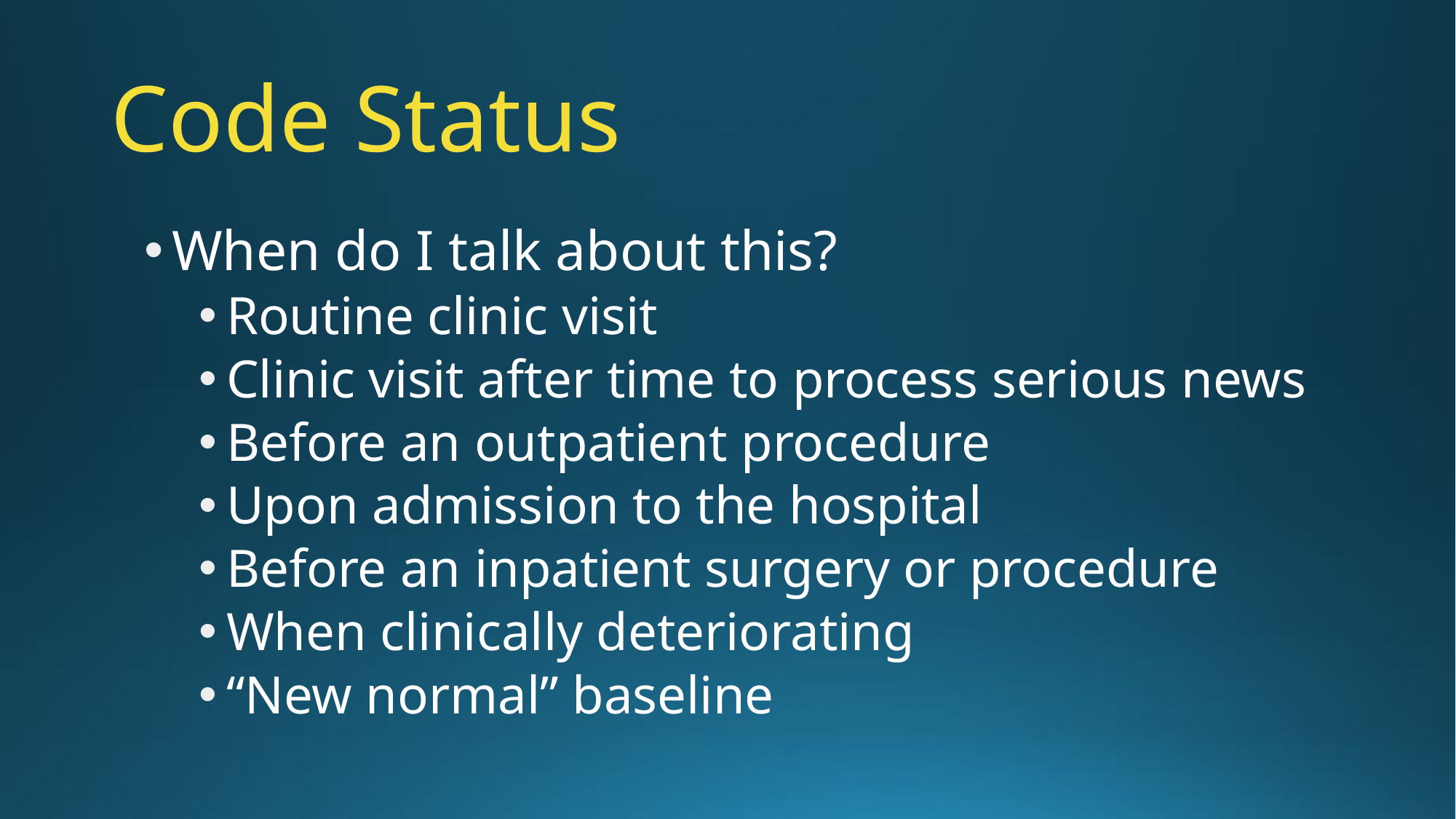

# Code Status
When do I talk about this?
Routine clinic visit
Clinic visit after time to process serious news
Before an outpatient procedure
Upon admission to the hospital
Before an inpatient surgery or procedure
When clinically deteriorating
“New normal” baseline

## Slide 7
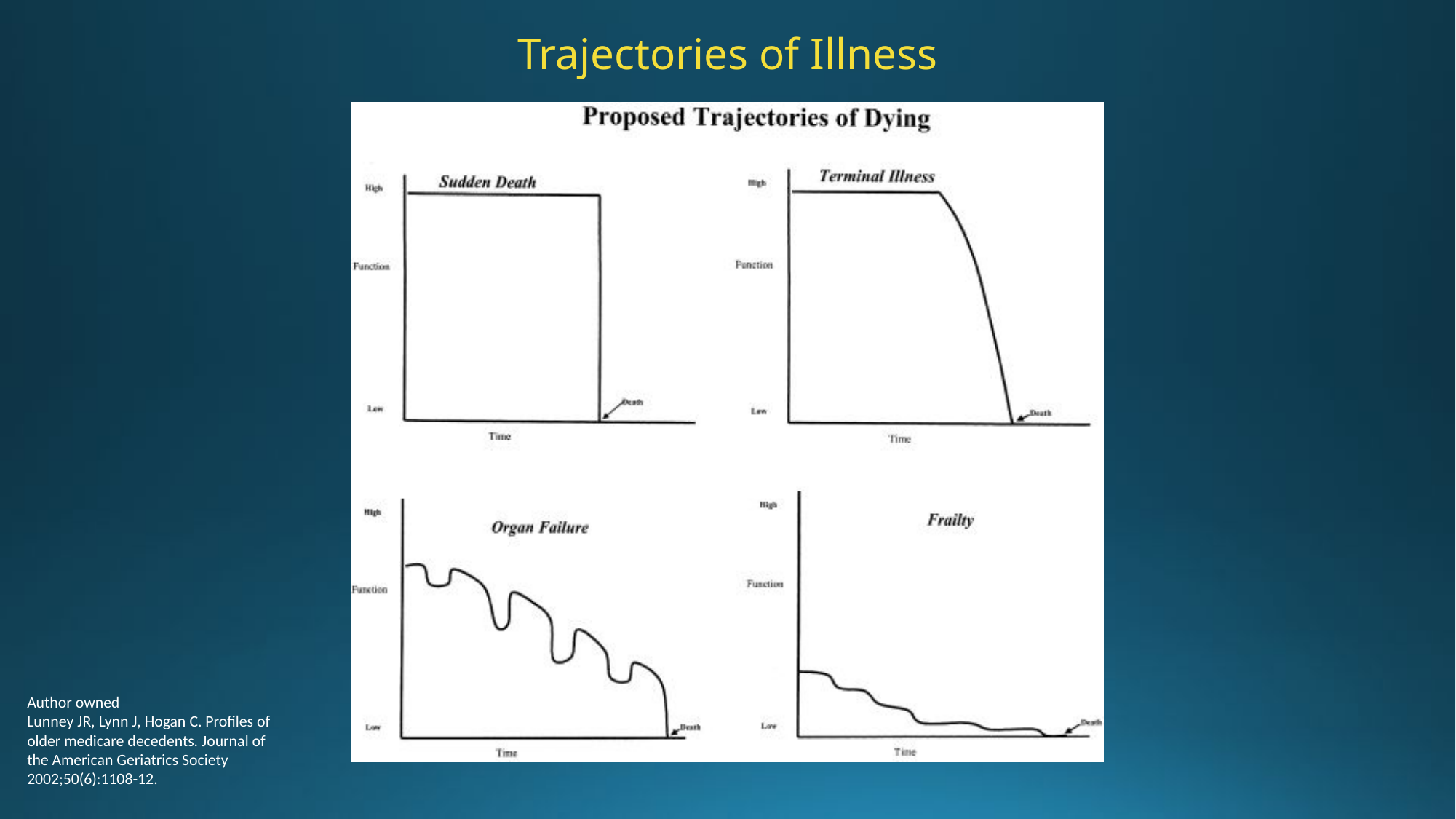

# Trajectories of Illness
Author owned
Lunney JR, Lynn J, Hogan C. Profiles of older medicare decedents. Journal of the American Geriatrics Society 2002;50(6):1108-12.

## Slide 8
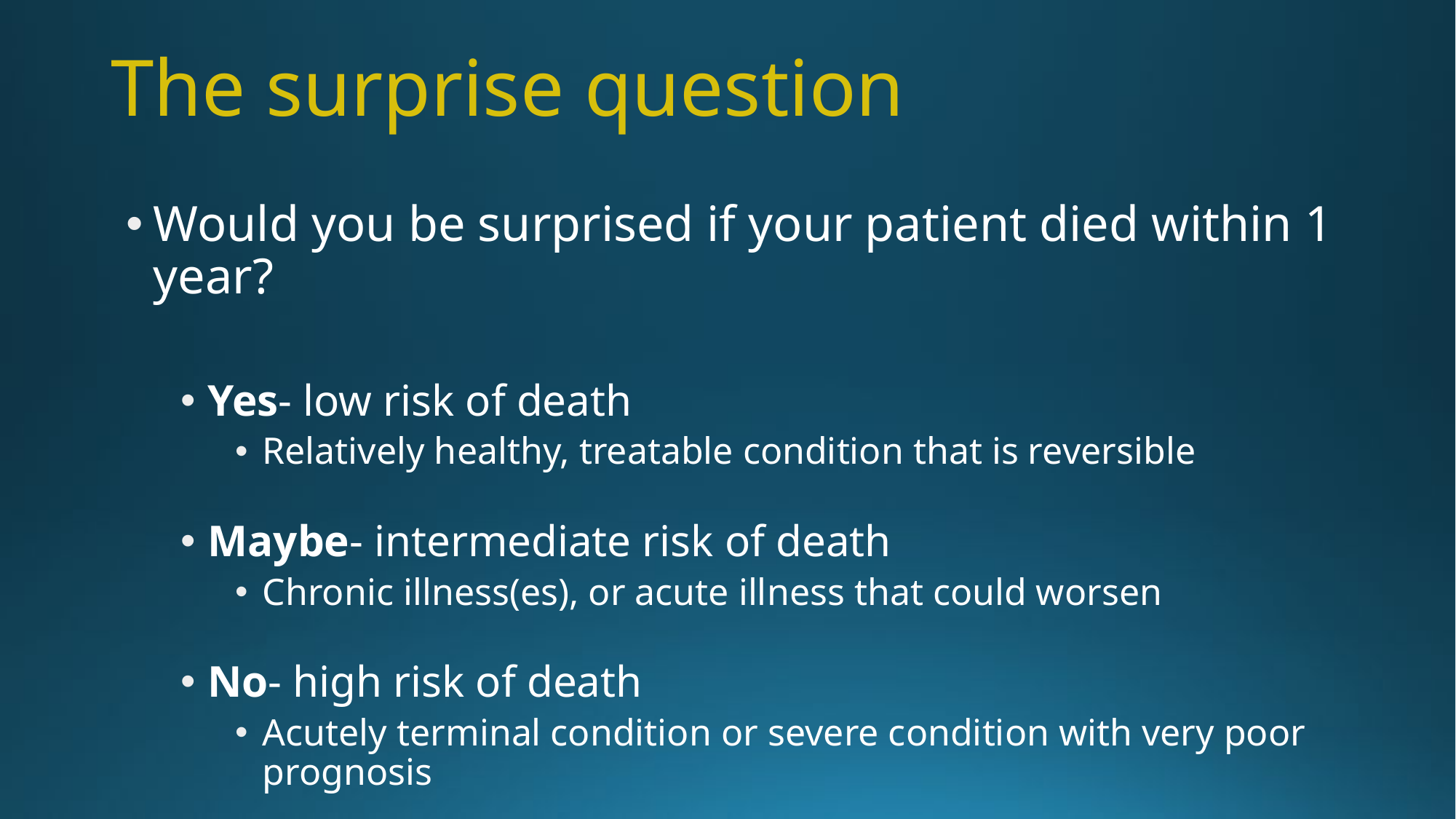

# The surprise question
Would you be surprised if your patient died within 1 year?
Yes- low risk of death
Relatively healthy, treatable condition that is reversible
Maybe- intermediate risk of death
Chronic illness(es), or acute illness that could worsen
No- high risk of death
Acutely terminal condition or severe condition with very poor prognosis

## Slide 9
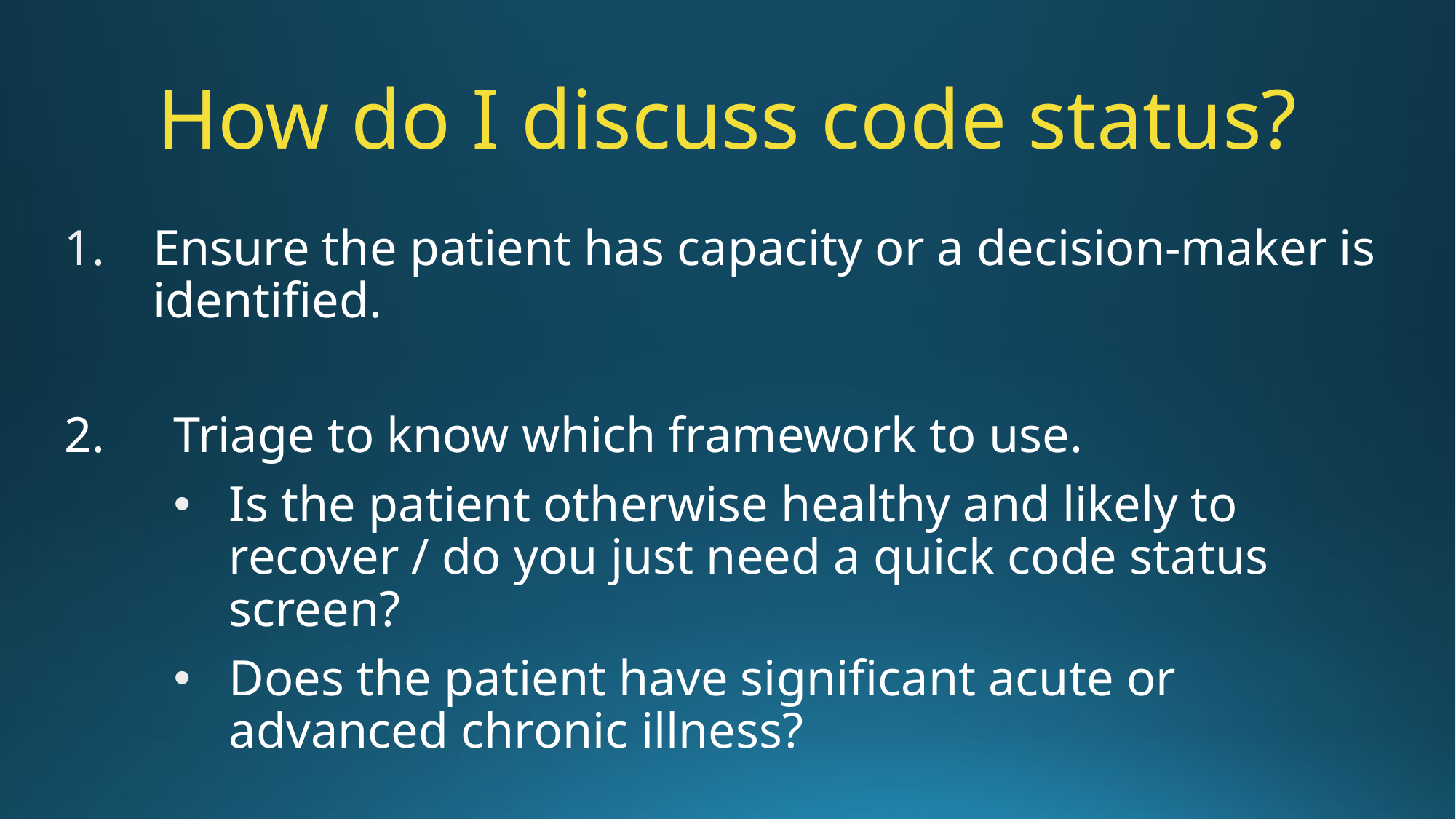

# How do I discuss code status?
Ensure the patient has capacity or a decision-maker is identified.
2.	Triage to know which framework to use.
Is the patient otherwise healthy and likely to recover / do you just need a quick code status screen?
Does the patient have significant acute or advanced chronic illness?

## Slide 10
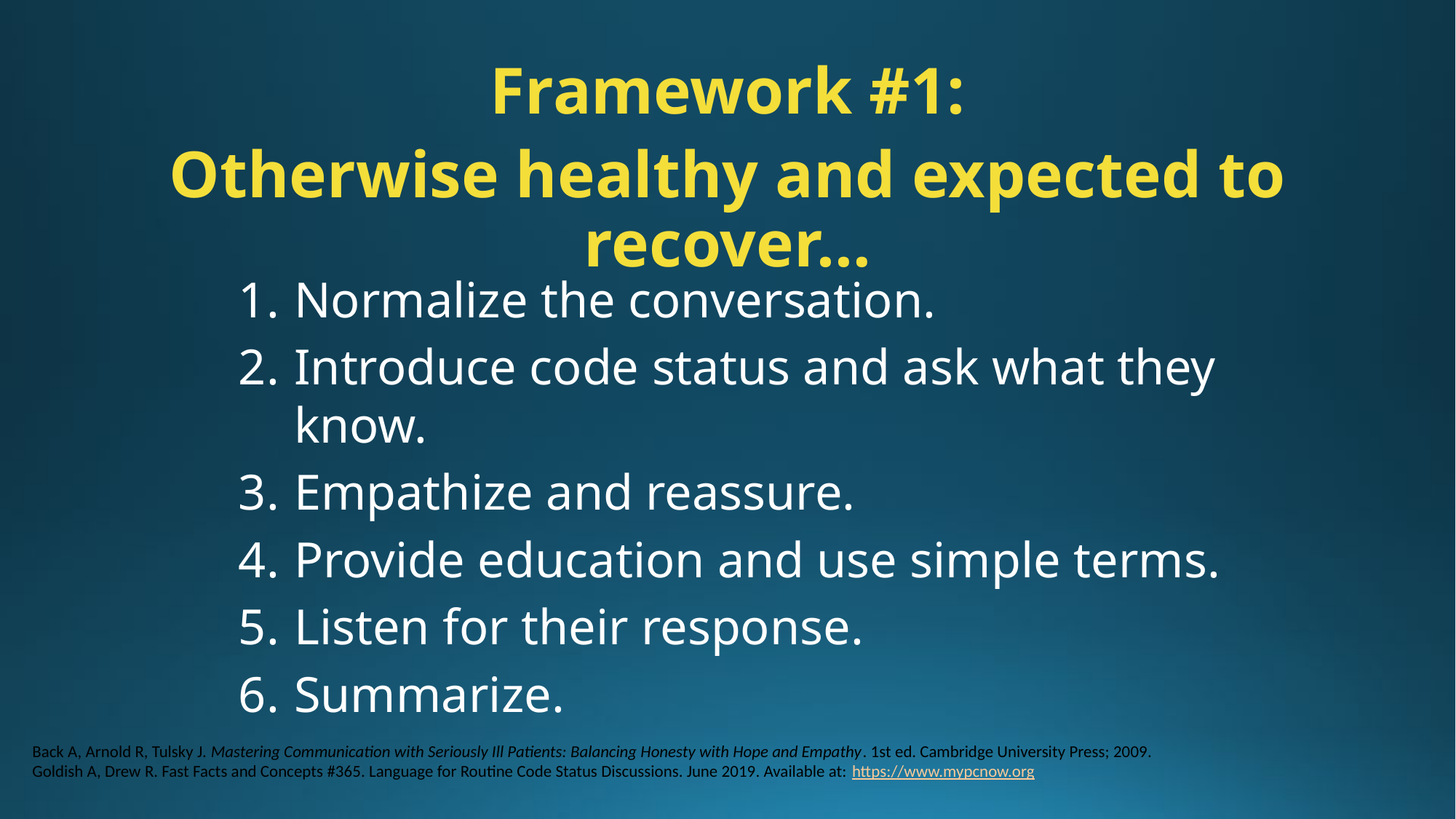

Framework #1:
Otherwise healthy and expected to recover…
Normalize the conversation.
Introduce code status and ask what they know.
Empathize and reassure.
Provide education and use simple terms.
Listen for their response.
Summarize.
Back A, Arnold R, Tulsky J. Mastering Communication with Seriously Ill Patients: Balancing Honesty with Hope and Empathy. 1st ed. Cambridge University Press; 2009.
Goldish A, Drew R. Fast Facts and Concepts #365. Language for Routine Code Status Discussions. June 2019. Available at: https://www.mypcnow.org

## Slide 11
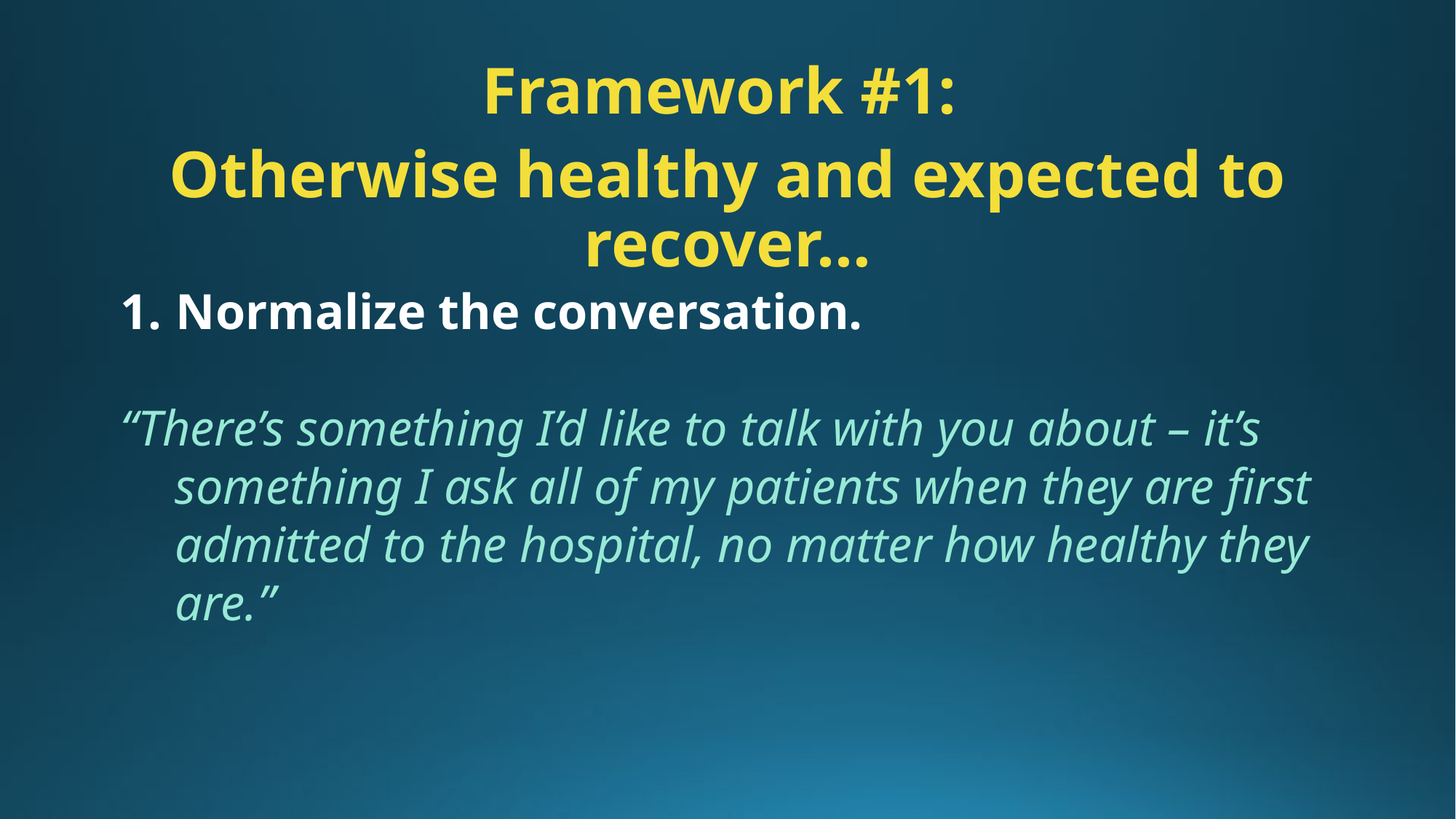

Framework #1:
Otherwise healthy and expected to recover…
1. 	Normalize the conversation.
“There’s something I’d like to talk with you about – it’s something I ask all of my patients when they are first admitted to the hospital, no matter how healthy they are.”

## Slide 12
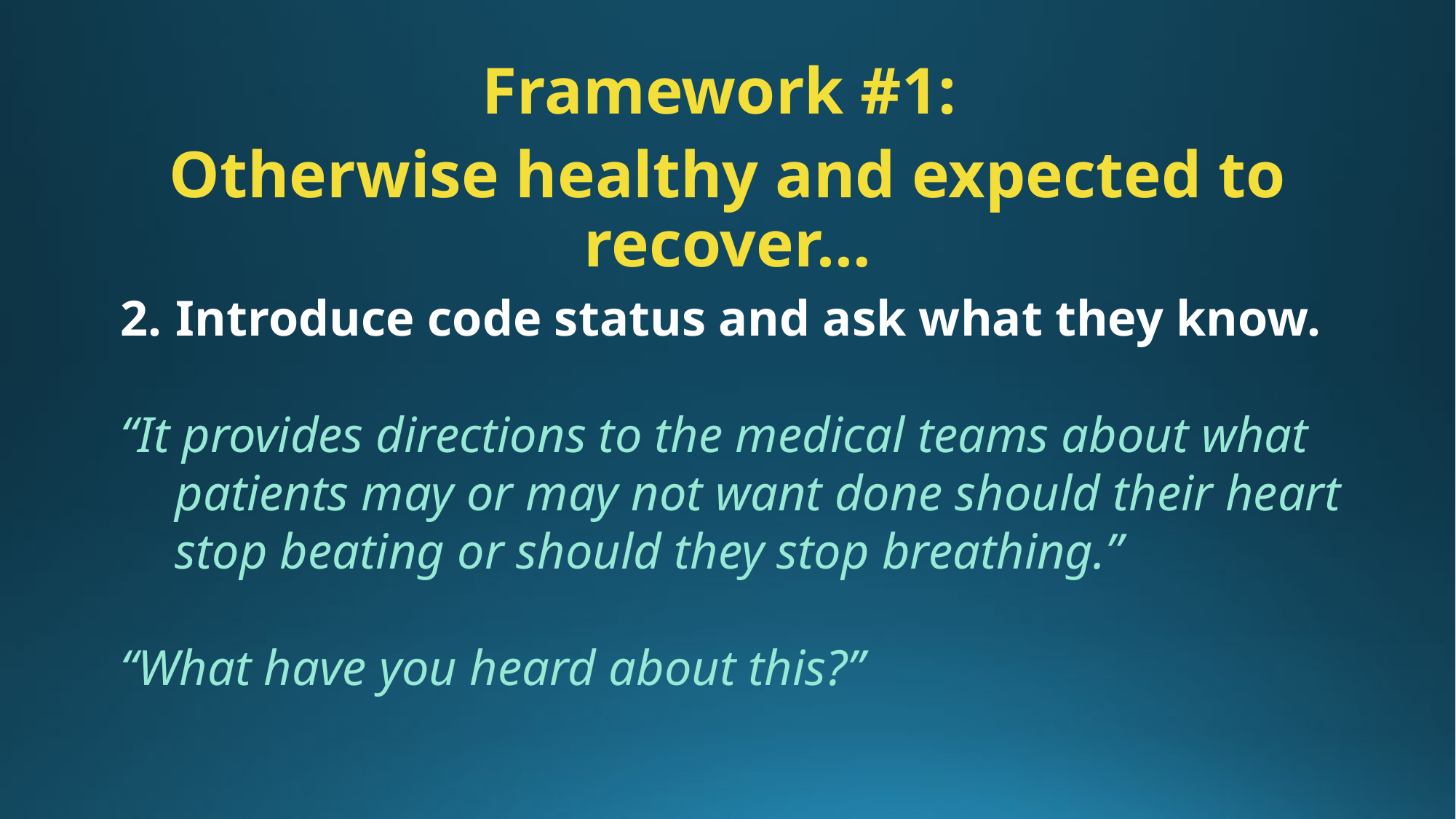

Framework #1:
Otherwise healthy and expected to recover…
2. 	Introduce code status and ask what they know.
“It provides directions to the medical teams about what patients may or may not want done should their heart stop beating or should they stop breathing.”
“What have you heard about this?”

## Slide 13
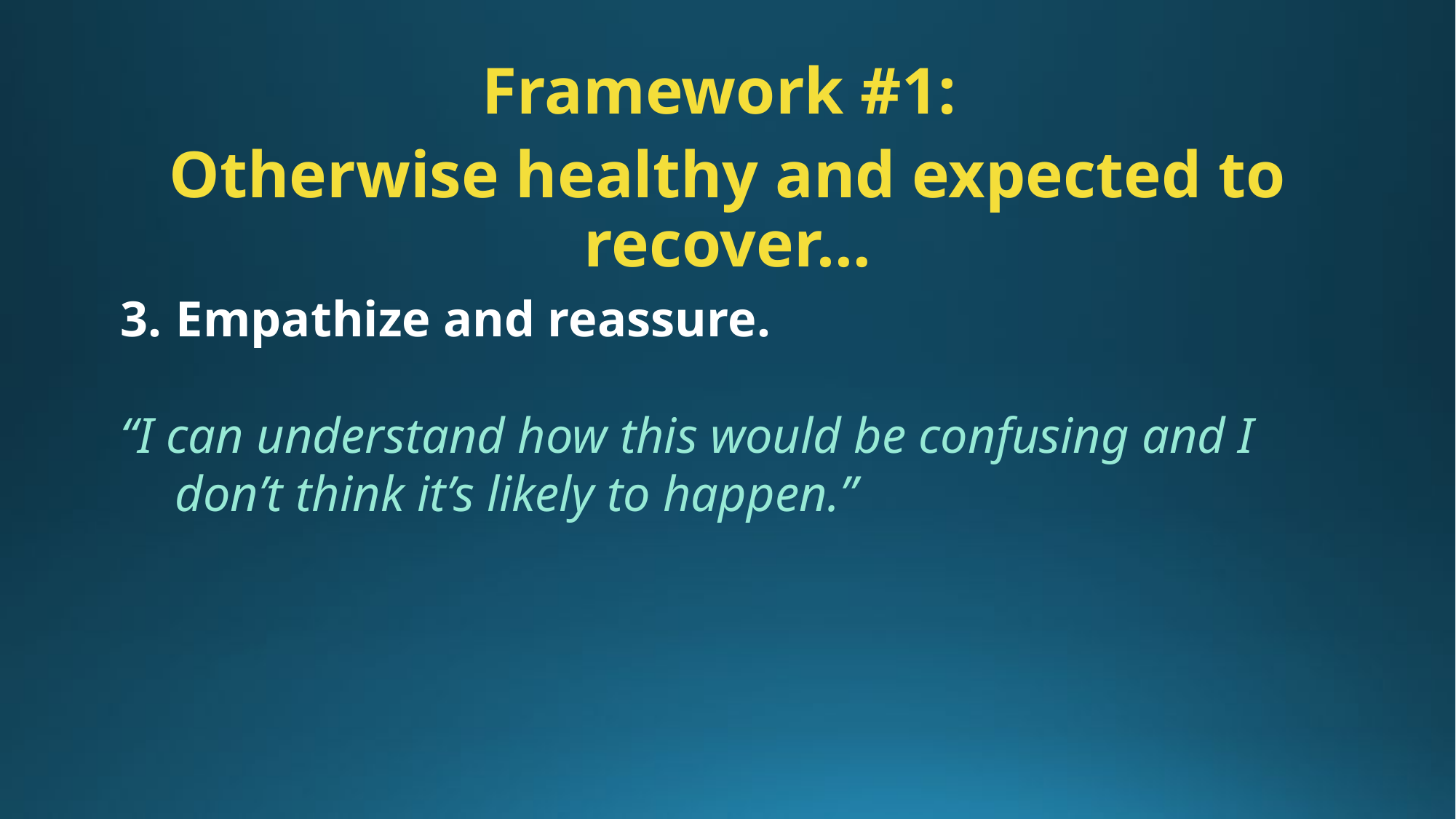

Framework #1:
Otherwise healthy and expected to recover…
3. 	Empathize and reassure.
“I can understand how this would be confusing and I don’t think it’s likely to happen.”

## Slide 14
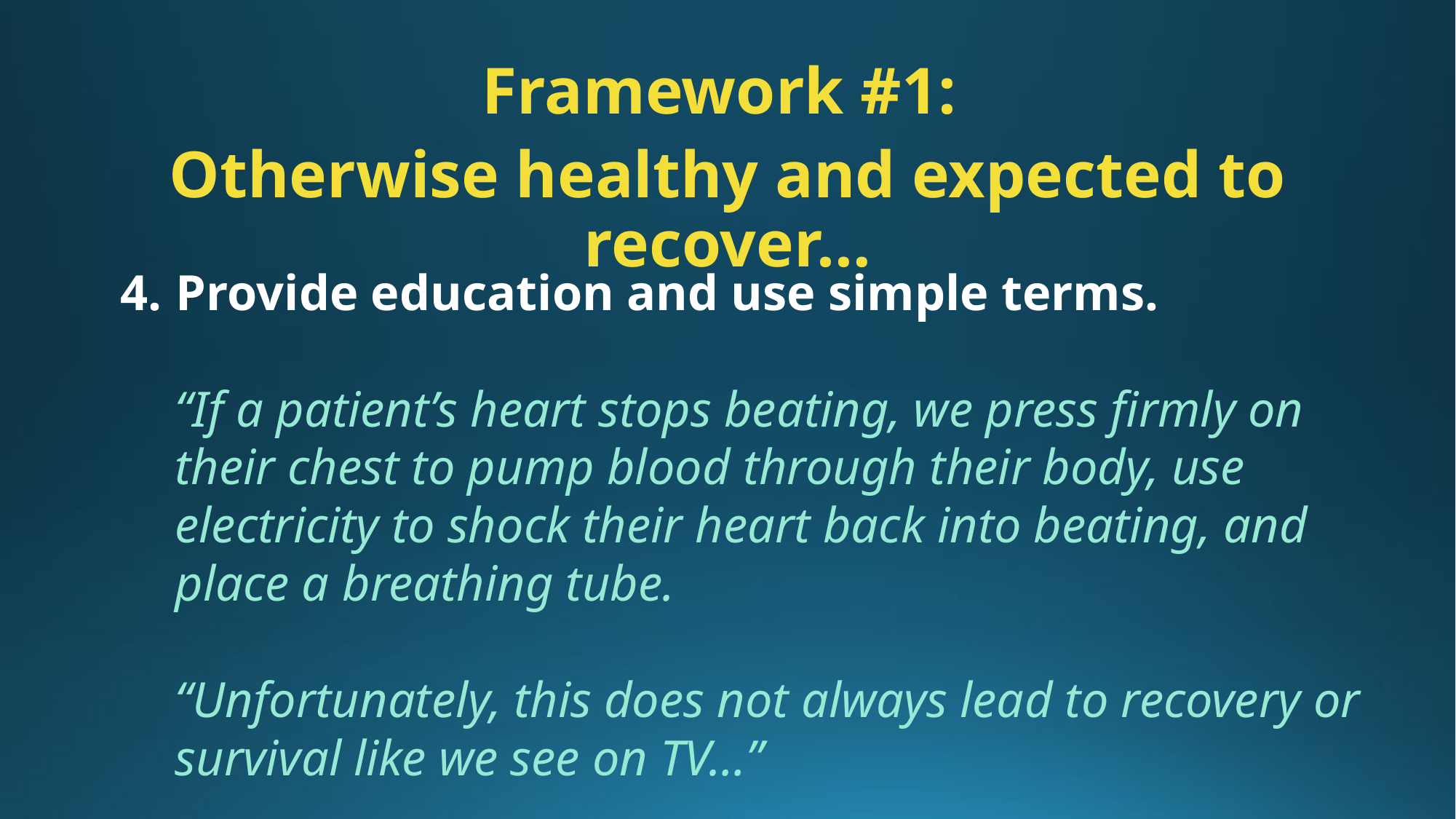

Framework #1:
Otherwise healthy and expected to recover…
Provide education and use simple terms.
“If a patient’s heart stops beating, we press firmly on their chest to pump blood through their body, use electricity to shock their heart back into beating, and place a breathing tube.
“Unfortunately, this does not always lead to recovery or survival like we see on TV…”

## Slide 15
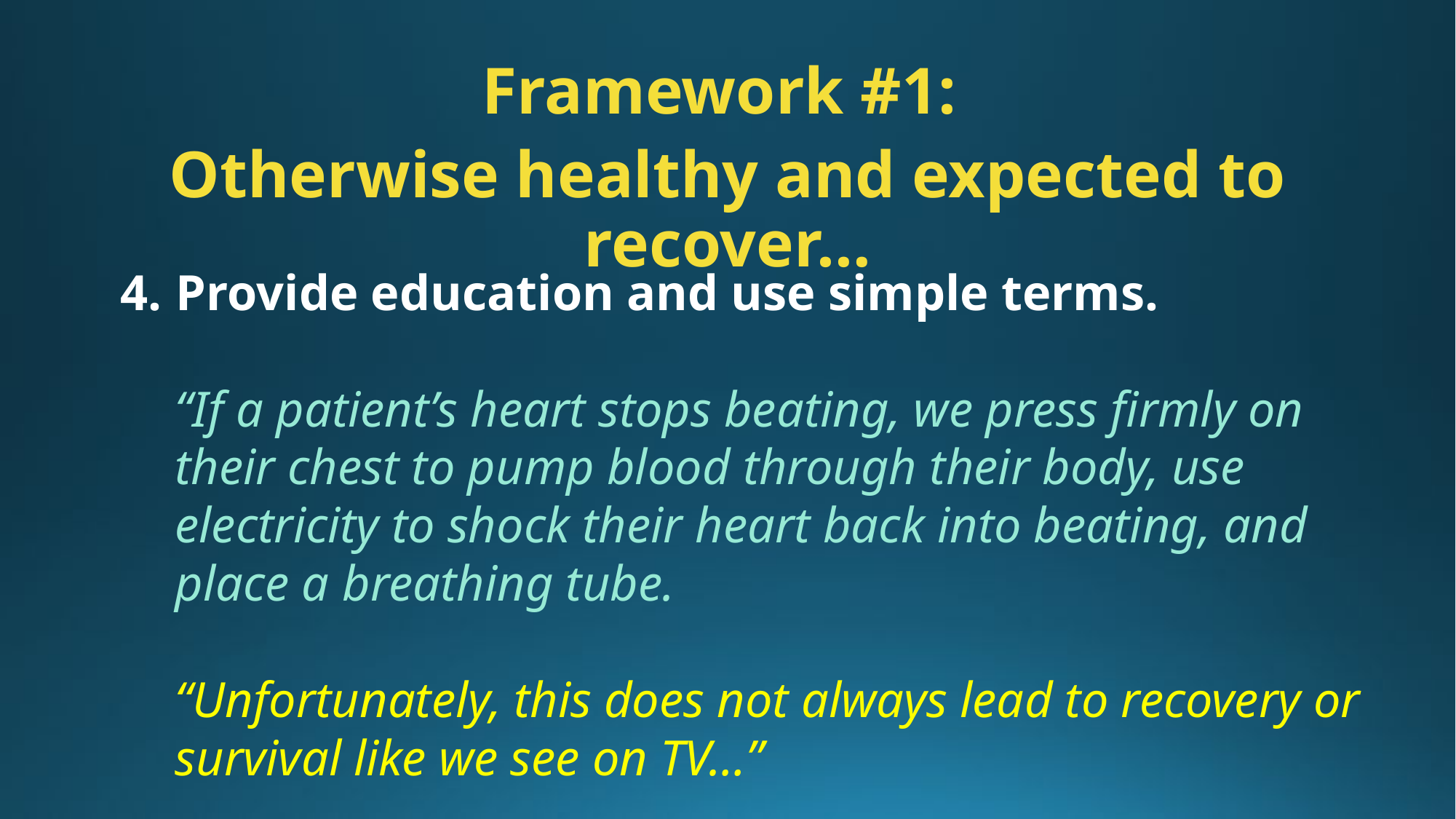

Framework #1:
Otherwise healthy and expected to recover…
Provide education and use simple terms.
“If a patient’s heart stops beating, we press firmly on their chest to pump blood through their body, use electricity to shock their heart back into beating, and place a breathing tube.
“Unfortunately, this does not always lead to recovery or survival like we see on TV…”

## Slide 16
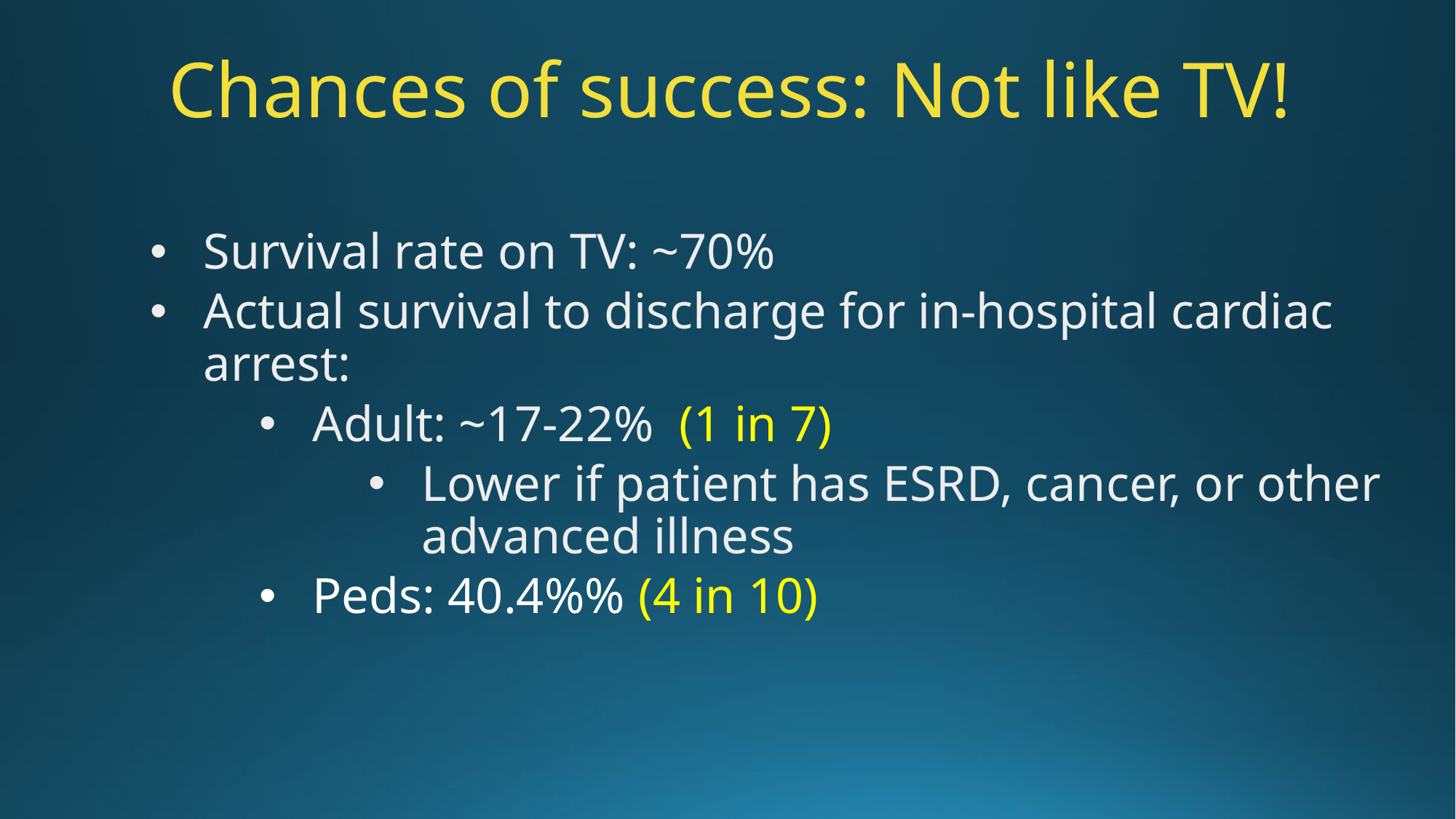

# Chances of success: Not like TV!
Survival rate on TV: ~70%
Actual survival to discharge for in-hospital cardiac arrest:
Adult: ~17-22% (1 in 7)
Lower if patient has ESRD, cancer, or other advanced illness
Peds: 40.4%% (4 in 10)

## Slide 17
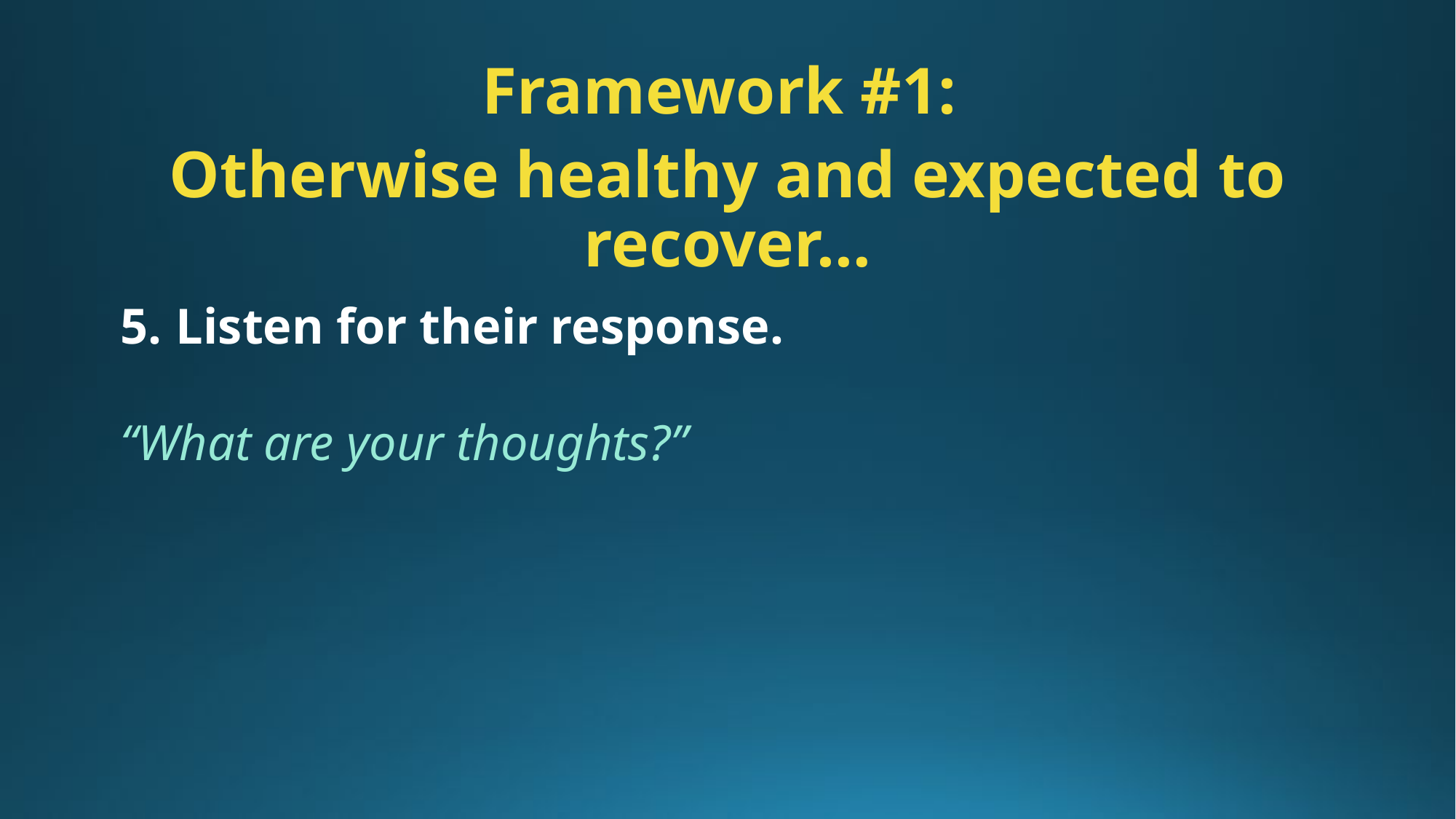

Framework #1:
Otherwise healthy and expected to recover…
5. 	Listen for their response.
“What are your thoughts?”

## Slide 18
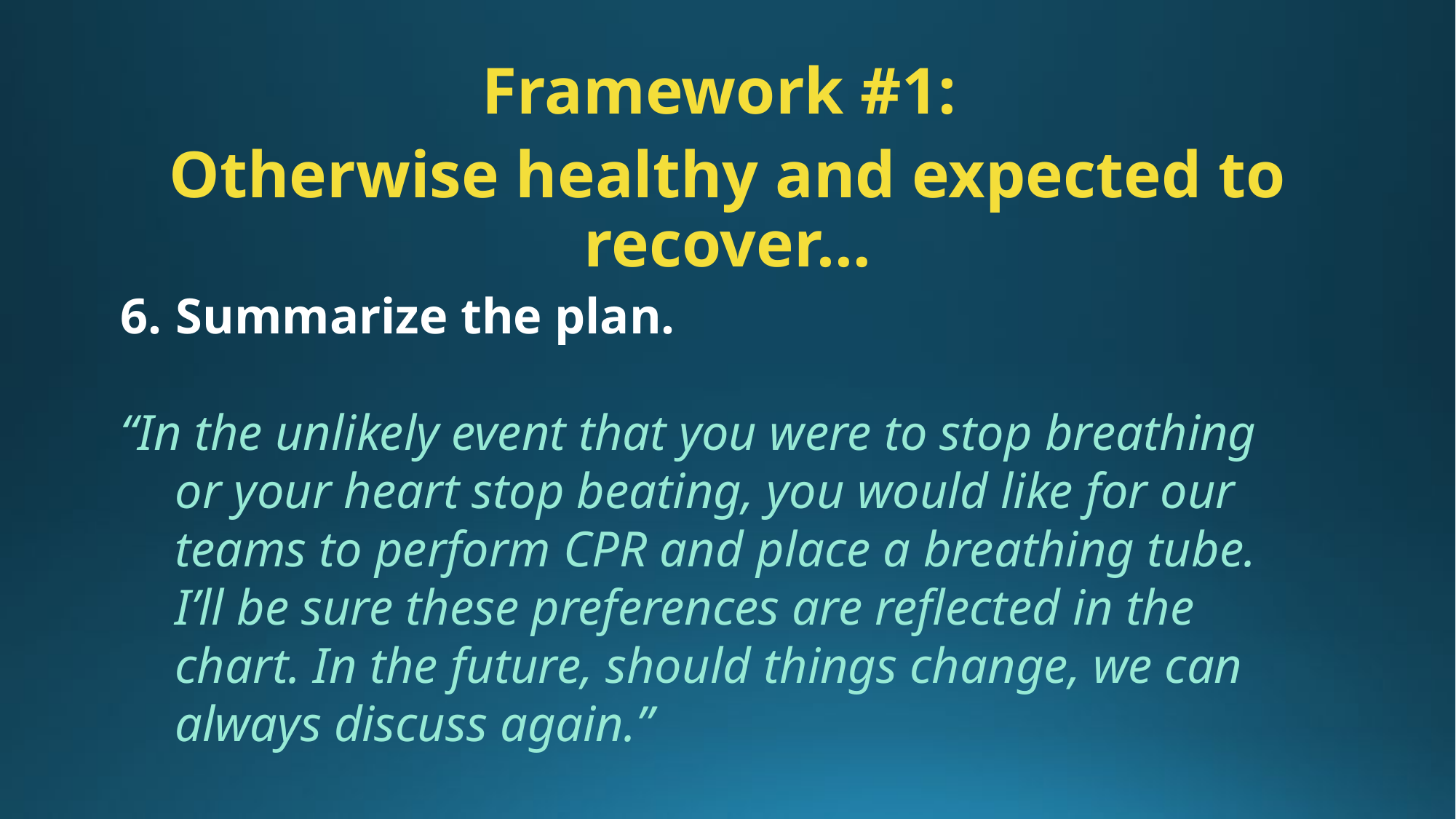

Framework #1:
Otherwise healthy and expected to recover…
6. 	Summarize the plan.
“In the unlikely event that you were to stop breathing or your heart stop beating, you would like for our teams to perform CPR and place a breathing tube. I’ll be sure these preferences are reflected in the chart. In the future, should things change, we can always discuss again.”

## Slide 19
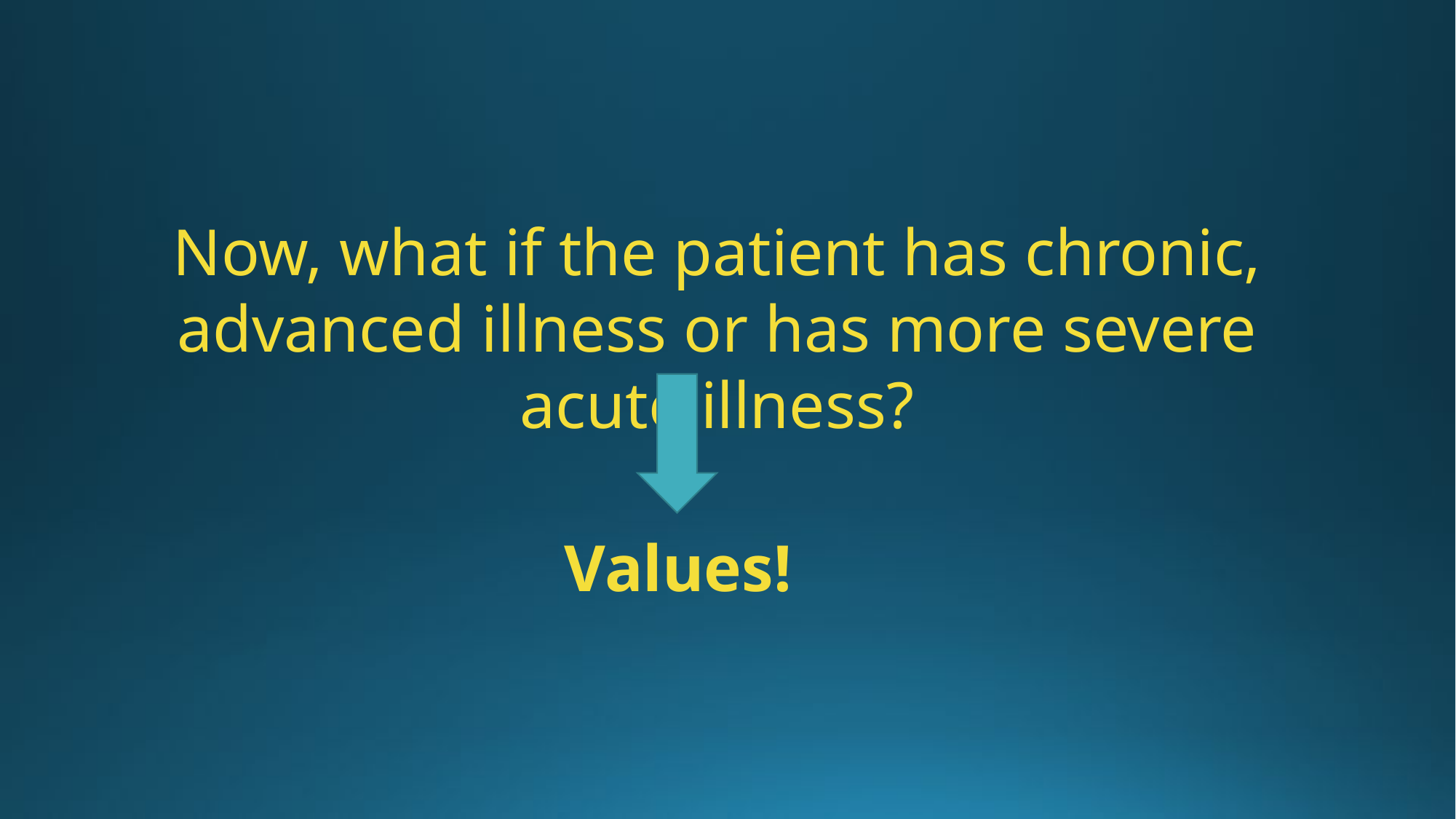

Now, what if the patient has chronic, advanced illness or has more severe acute illness?
Values!

## Slide 20
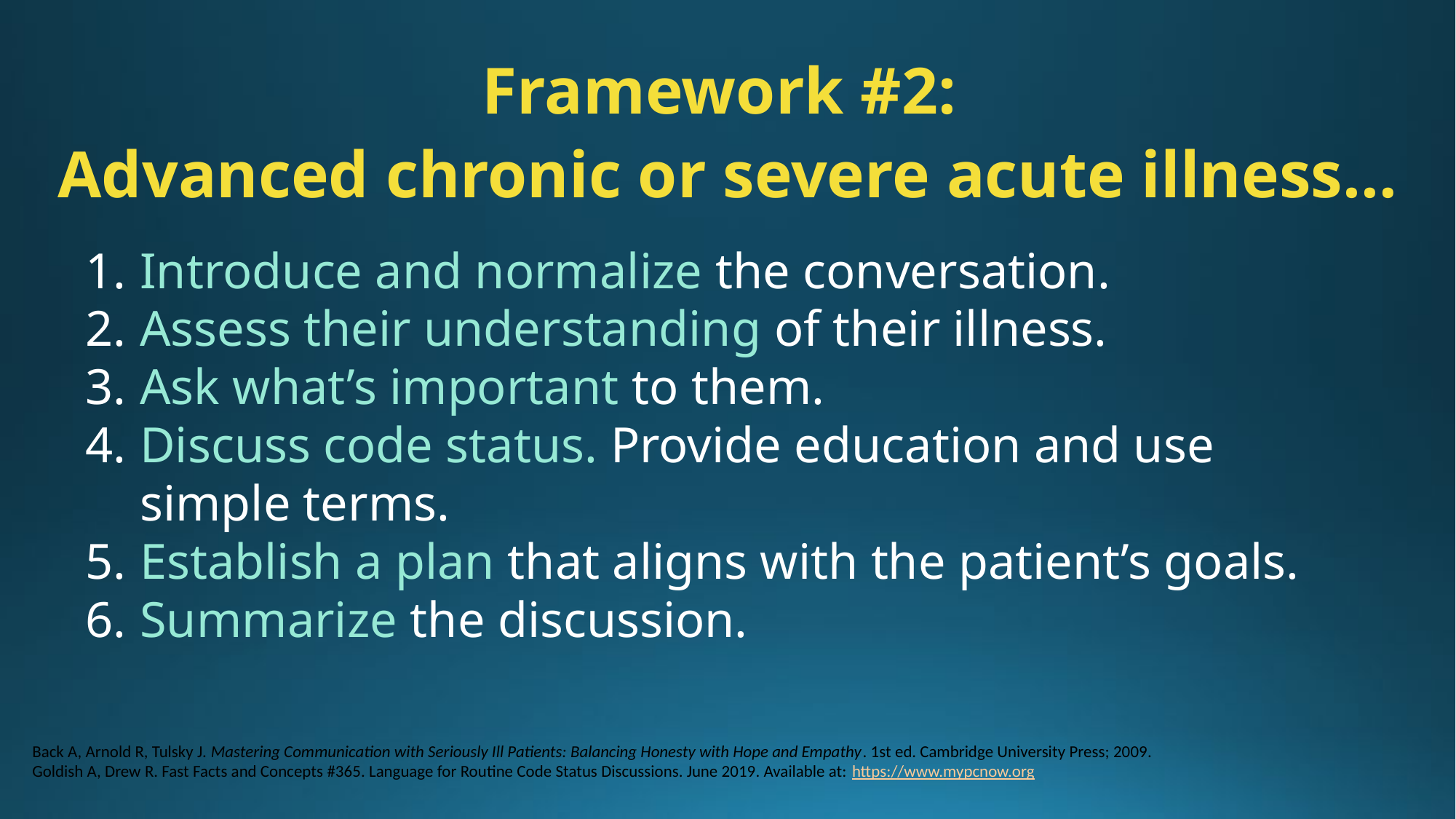

Framework #2:
Advanced chronic or severe acute illness…
Introduce and normalize the conversation.
Assess their understanding of their illness.
Ask what’s important to them.
Discuss code status. Provide education and use simple terms.
Establish a plan that aligns with the patient’s goals.
Summarize the discussion.
Back A, Arnold R, Tulsky J. Mastering Communication with Seriously Ill Patients: Balancing Honesty with Hope and Empathy. 1st ed. Cambridge University Press; 2009.
Goldish A, Drew R. Fast Facts and Concepts #365. Language for Routine Code Status Discussions. June 2019. Available at: https://www.mypcnow.org

## Slide 21
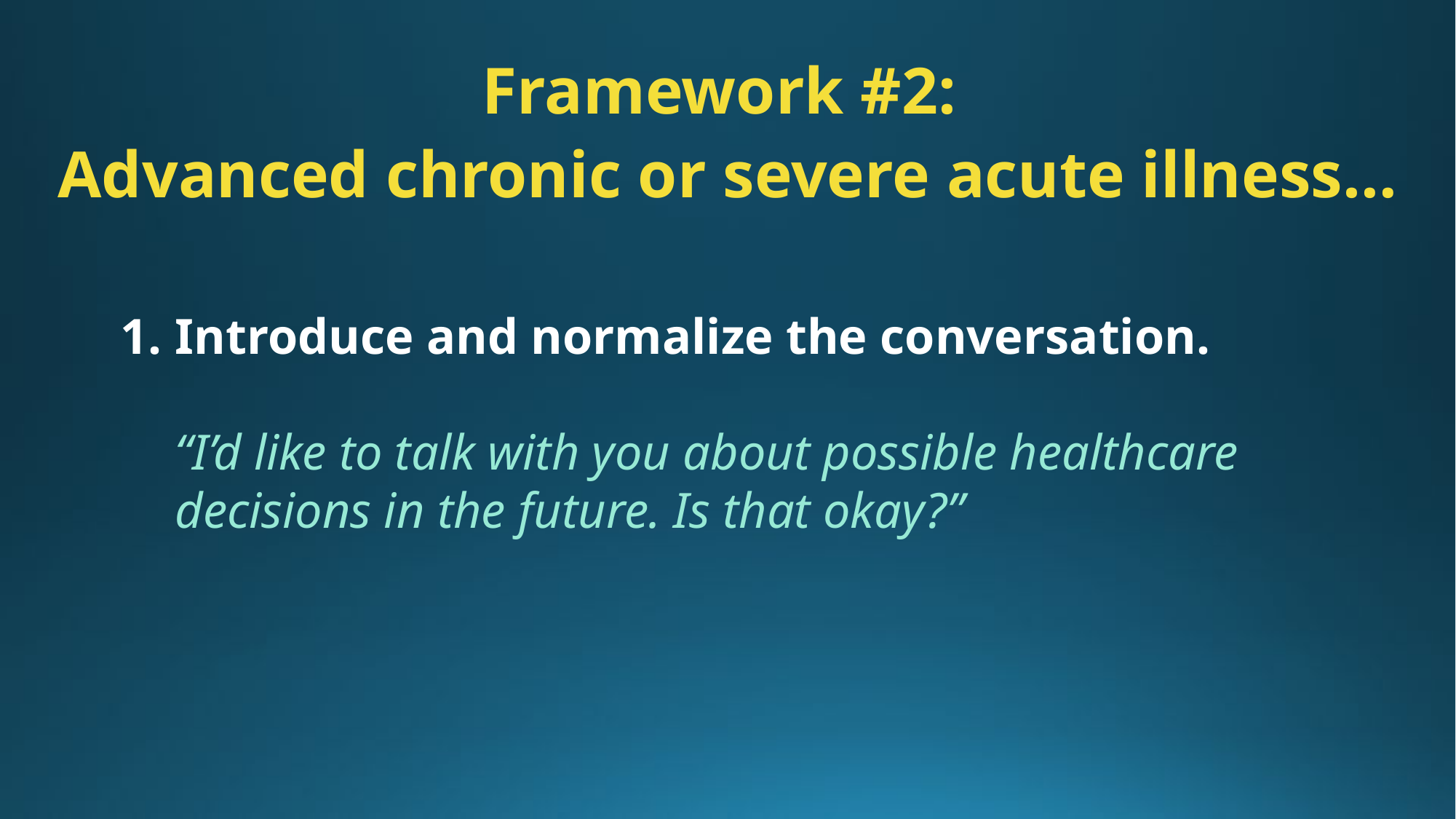

Framework #2:
Advanced chronic or severe acute illness…
Introduce and normalize the conversation.
“I’d like to talk with you about possible healthcare decisions in the future. Is that okay?”

## Slide 22
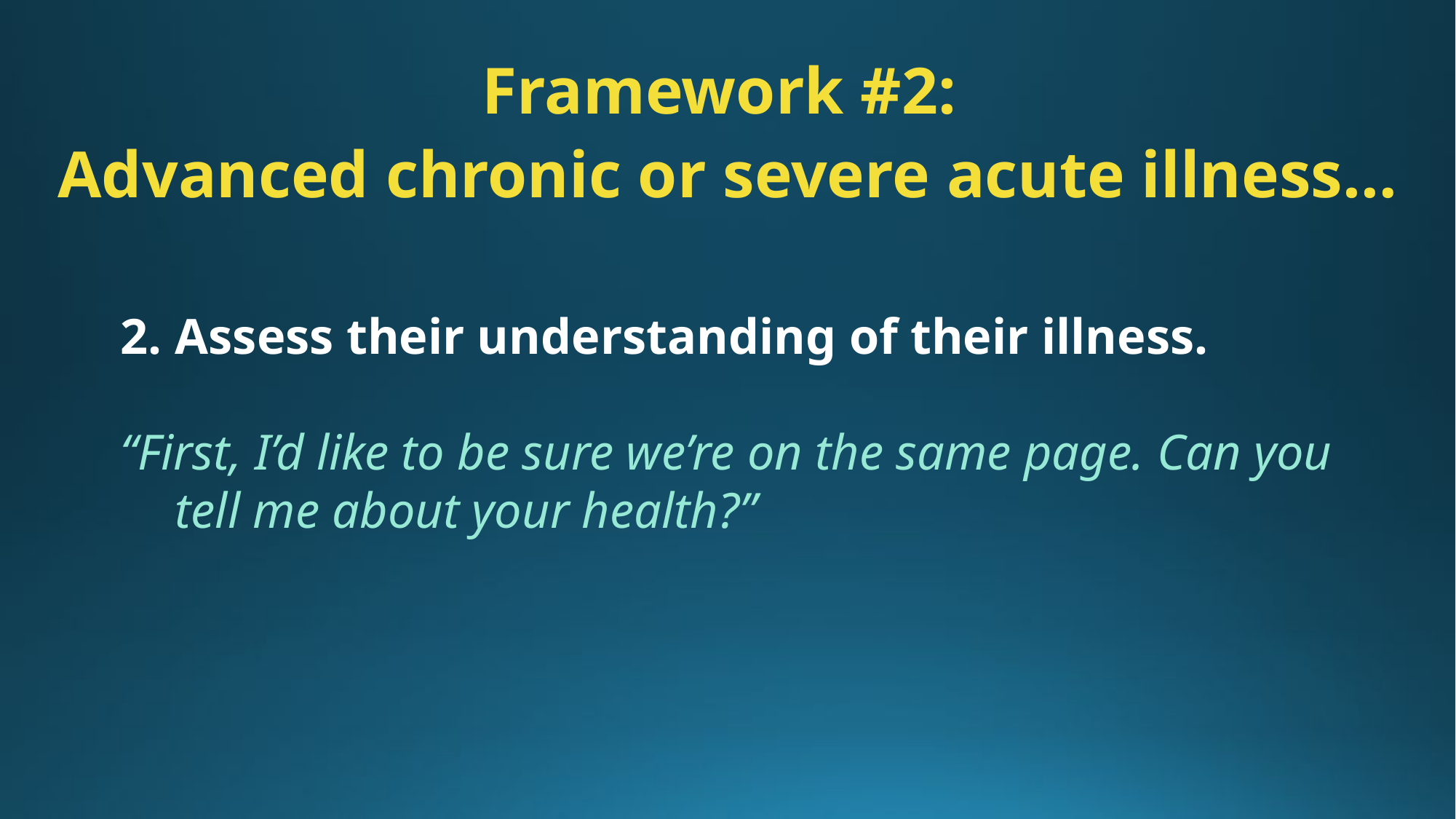

Framework #2:
Advanced chronic or severe acute illness…
2. 	Assess their understanding of their illness.
“First, I’d like to be sure we’re on the same page. Can you tell me about your health?”

## Slide 23
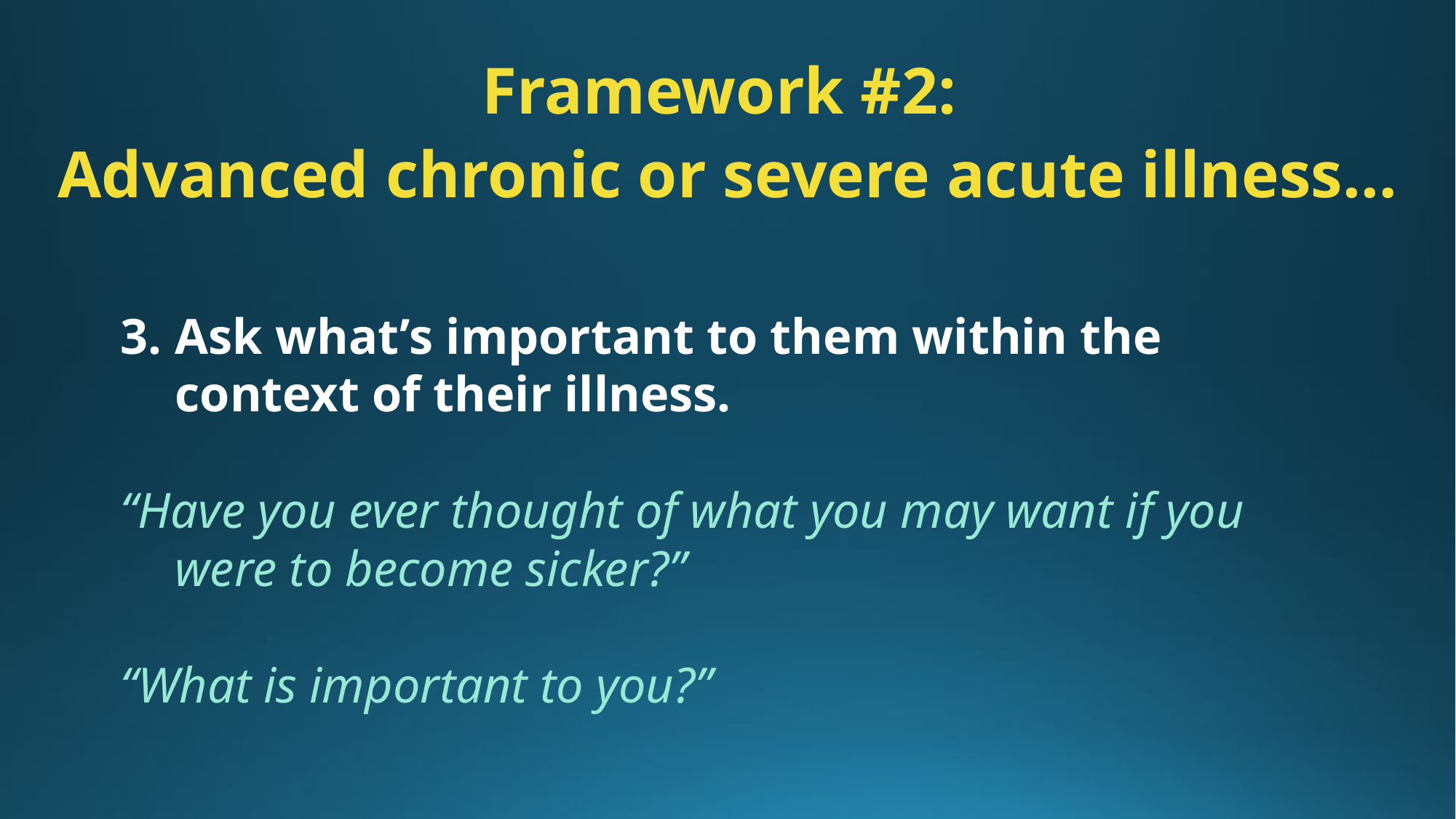

Framework #2:
Advanced chronic or severe acute illness…
3. 	Ask what’s important to them within the context of their illness.
“Have you ever thought of what you may want if you were to become sicker?”
“What is important to you?”

## Slide 24
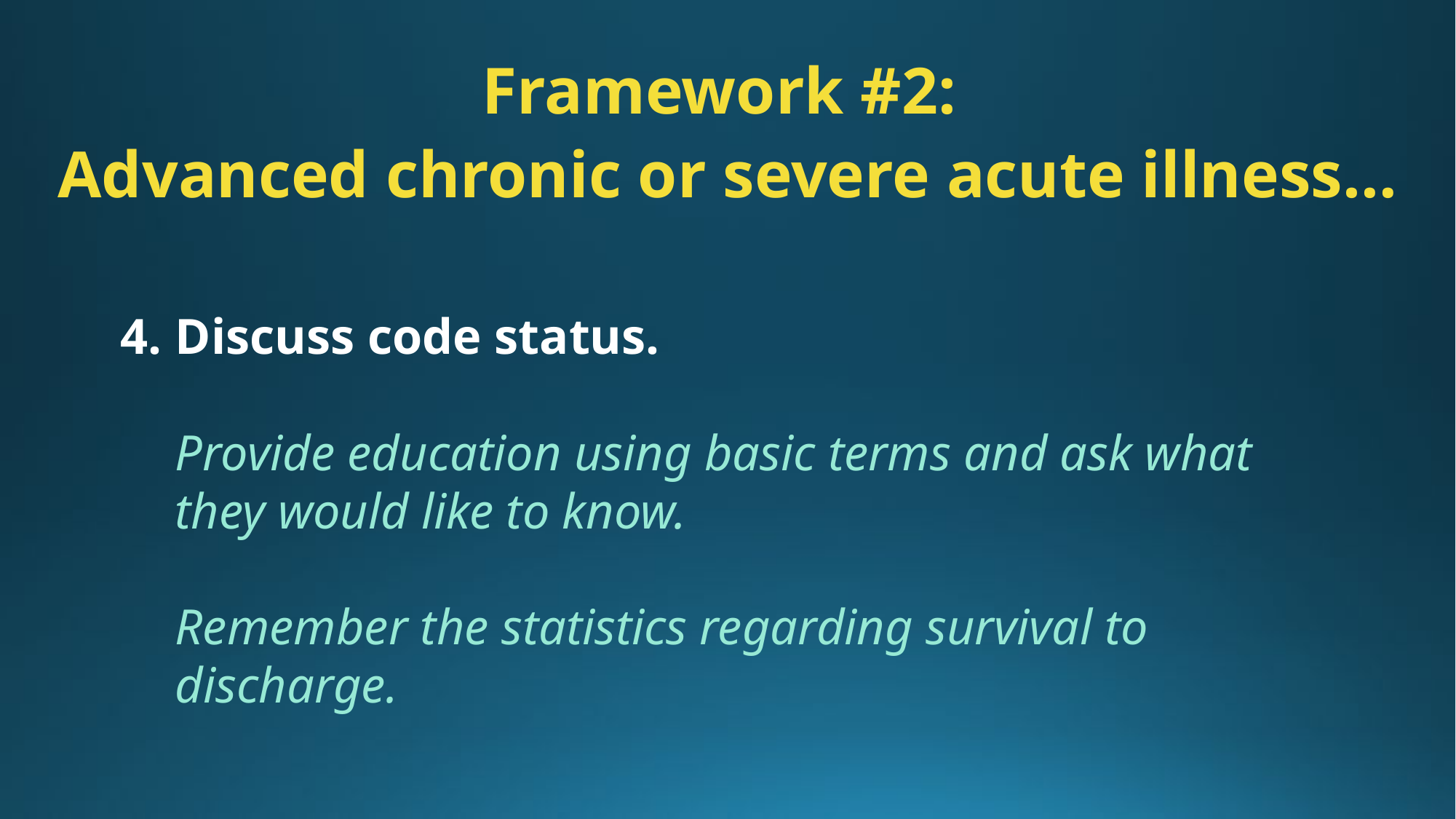

Framework #2:
Advanced chronic or severe acute illness…
Discuss code status.
Provide education using basic terms and ask what they would like to know.
Remember the statistics regarding survival to discharge.

## Slide 25
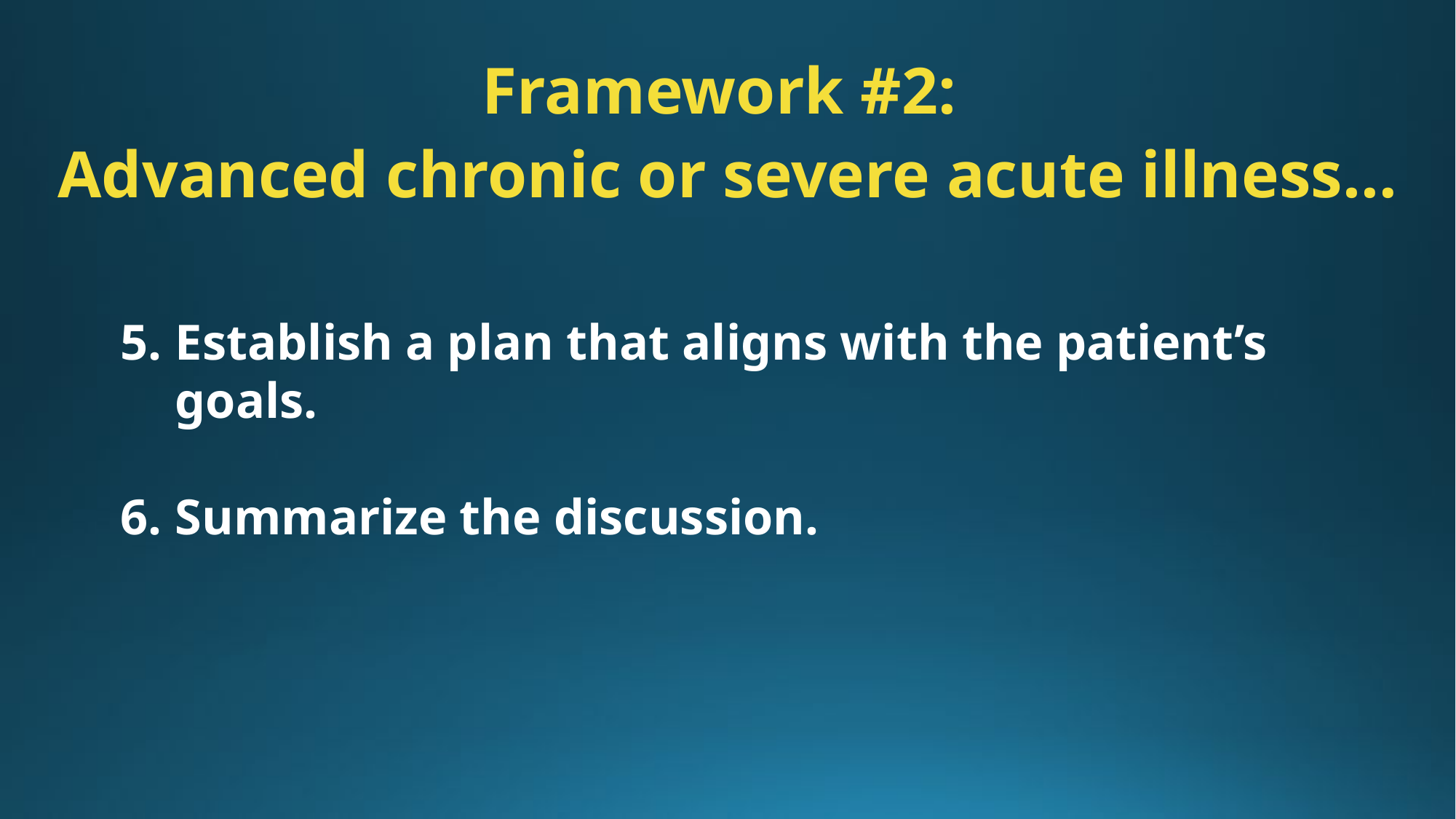

Framework #2:
Advanced chronic or severe acute illness…
Establish a plan that aligns with the patient’s goals.
Summarize the discussion.

## Slide 26
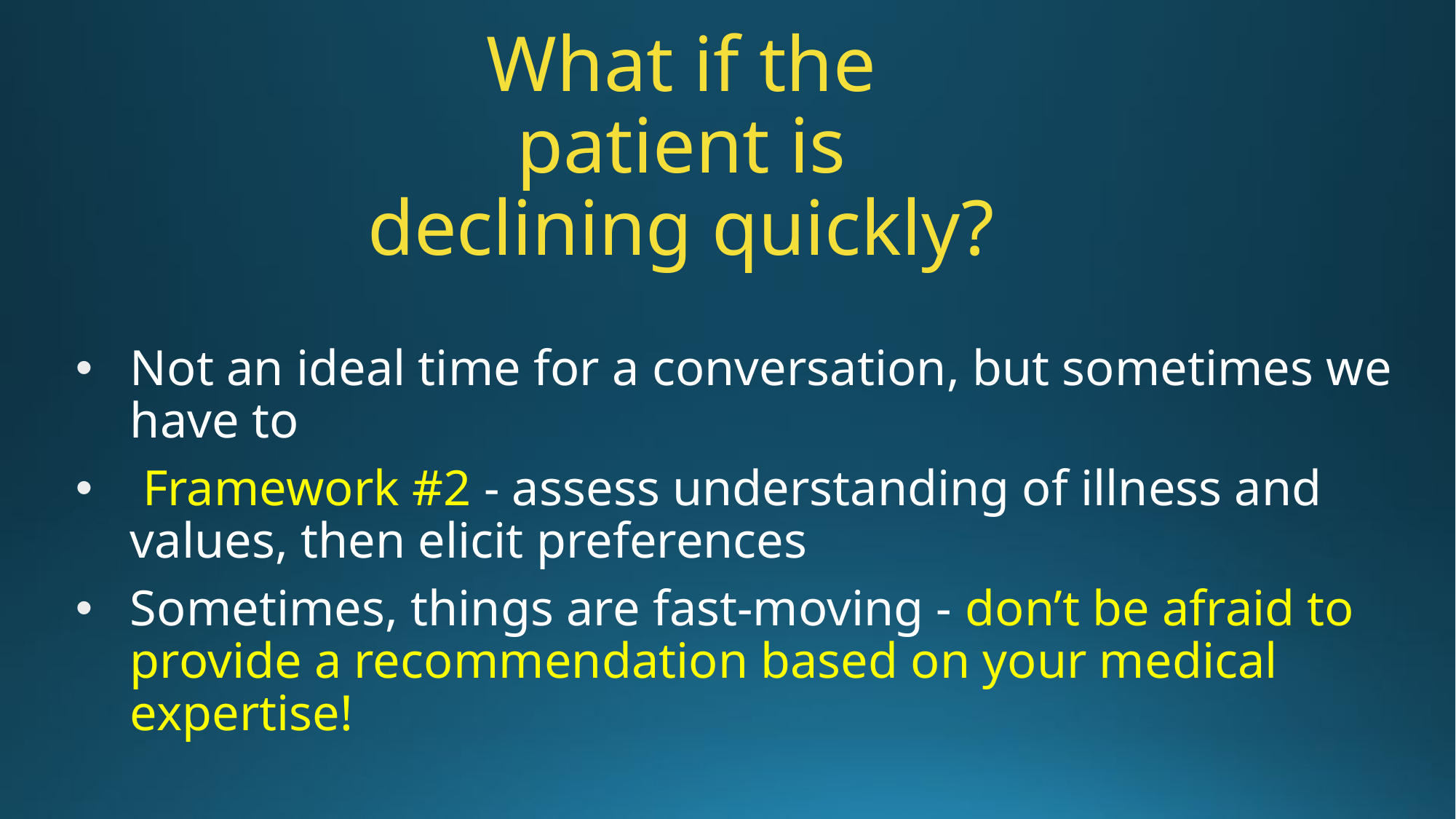

# What if the patient is declining quickly?
Not an ideal time for a conversation, but sometimes we have to
 Framework #2 - assess understanding of illness and values, then elicit preferences
Sometimes, things are fast-moving - don’t be afraid to provide a recommendation based on your medical expertise!

## Slide 27
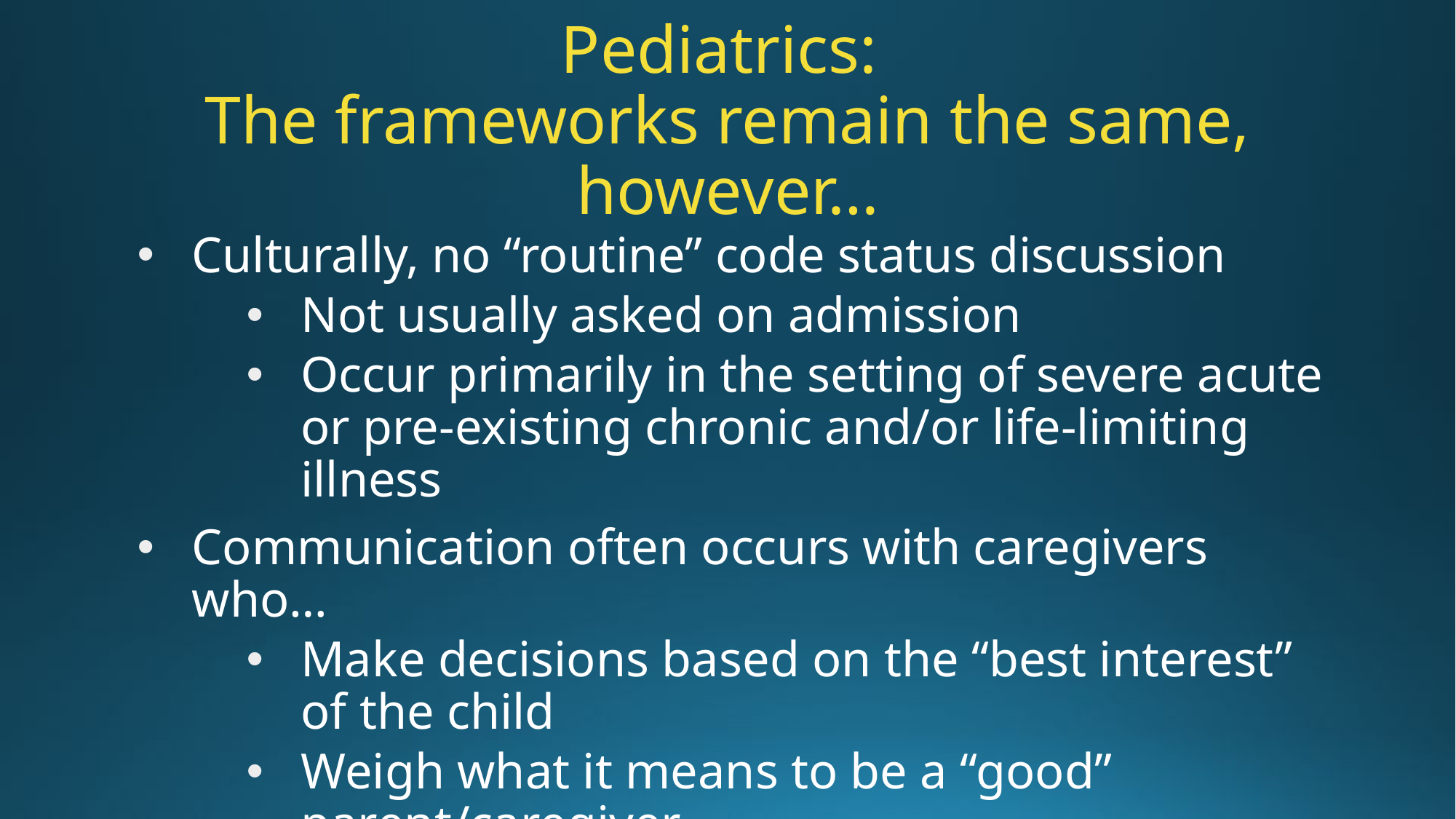

# Pediatrics: The frameworks remain the same, however…
Culturally, no “routine” code status discussion
Not usually asked on admission
Occur primarily in the setting of severe acute or pre-existing chronic and/or life-limiting illness
Communication often occurs with caregivers who…
Make decisions based on the “best interest” of the child
Weigh what it means to be a “good” parent/caregiver

## Slide 28
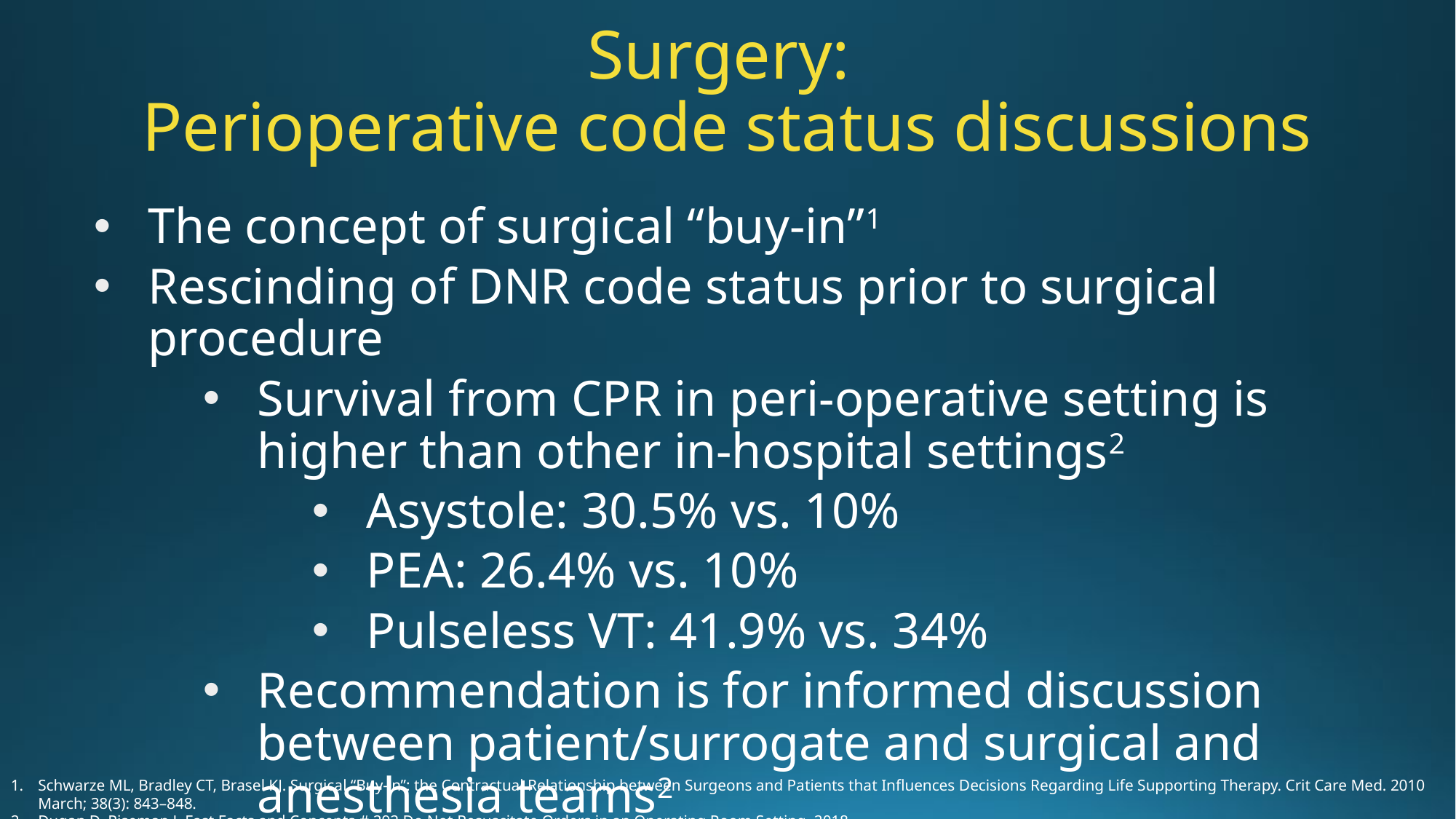

# Surgery: Perioperative code status discussions
The concept of surgical “buy-in”1
Rescinding of DNR code status prior to surgical procedure
Survival from CPR in peri-operative setting is higher than other in-hospital settings2
Asystole: 30.5% vs. 10%
PEA: 26.4% vs. 10%
Pulseless VT: 41.9% vs. 34%
Recommendation is for informed discussion between patient/surrogate and surgical and anesthesia teams2
Schwarze ML, Bradley CT, Brasel KJ. Surgical “Buy-in”: the Contractual Relationship between Surgeons and Patients that Influences Decisions Regarding Life Supporting Therapy. Crit Care Med. 2010 March; 38(3): 843–848.
Dugan D, Riseman J. Fast Facts and Concepts # 292 Do Not Resuscitate Orders in an Operating Room Setting. 2018.

## Slide 29
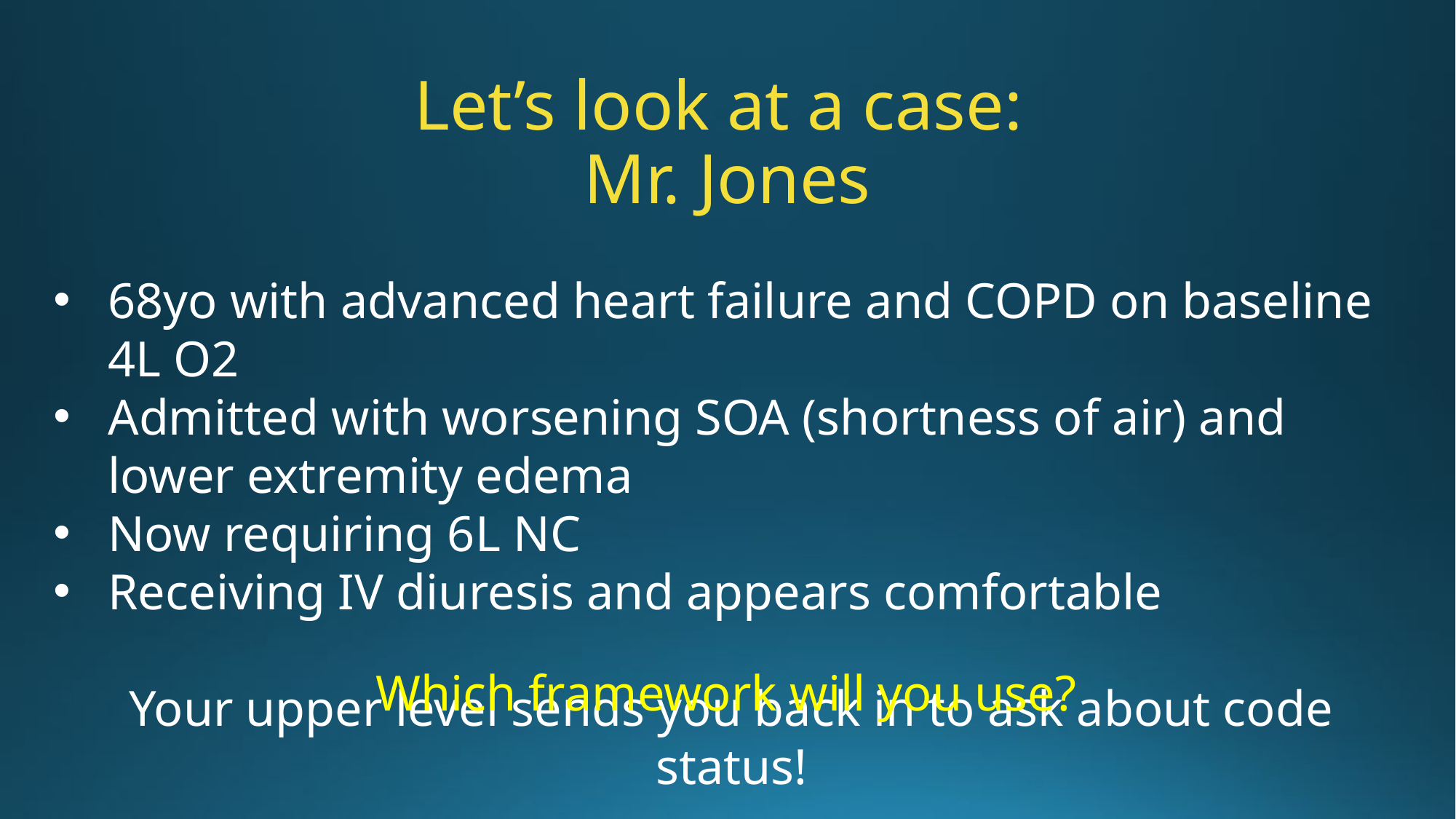

# Let’s look at a case: Mr. Jones
68yo with advanced heart failure and COPD on baseline 4L O2
Admitted with worsening SOA (shortness of air) and lower extremity edema
Now requiring 6L NC
Receiving IV diuresis and appears comfortable
Your upper level sends you back in to ask about code status!
Which framework will you use?

## Slide 30
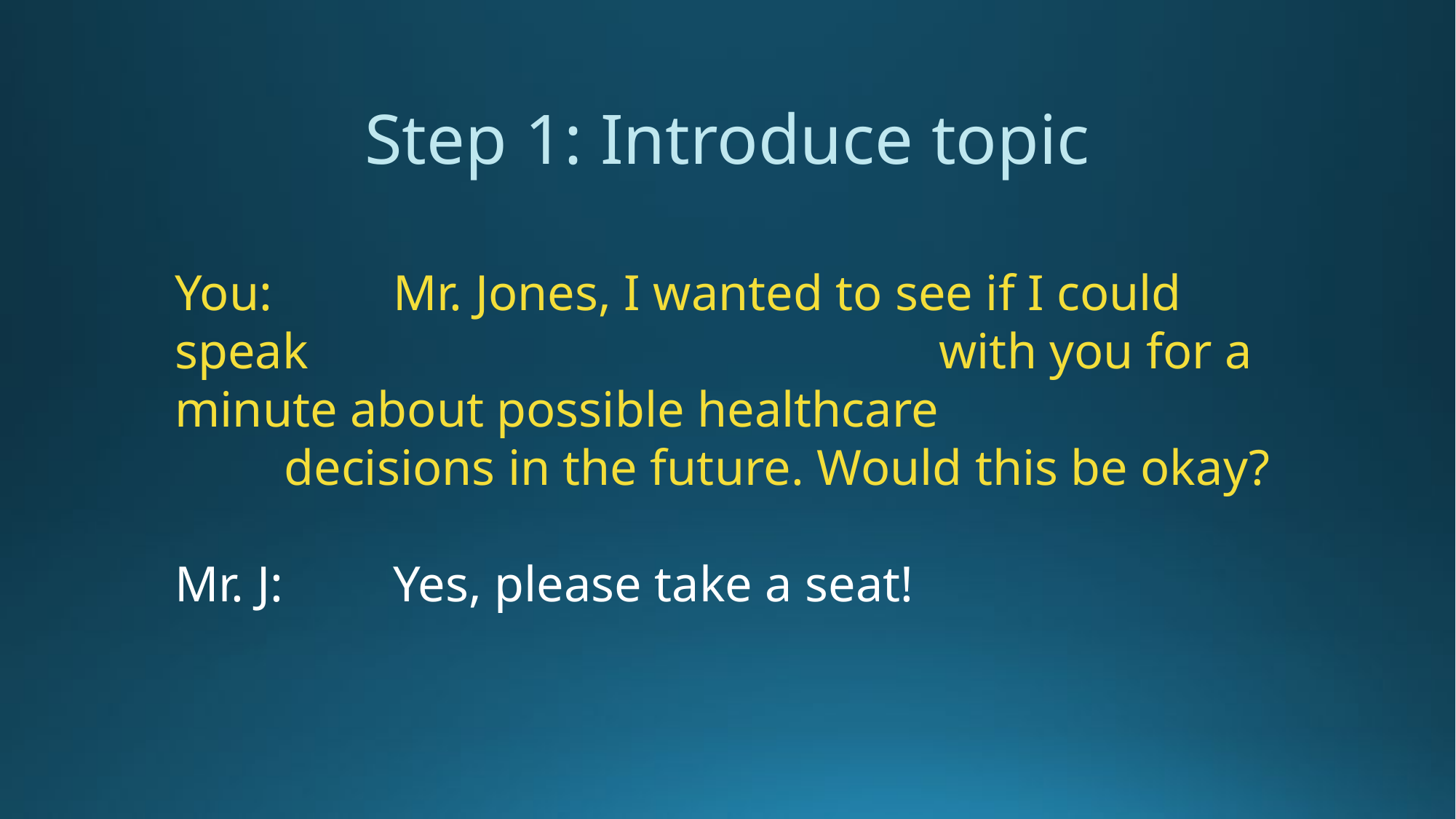

Step 1: Introduce topic
You:		Mr. Jones, I wanted to see if I could speak 						with you for a minute about possible healthcare 				decisions in the future. Would this be okay?
Mr. J:		Yes, please take a seat!

## Slide 31
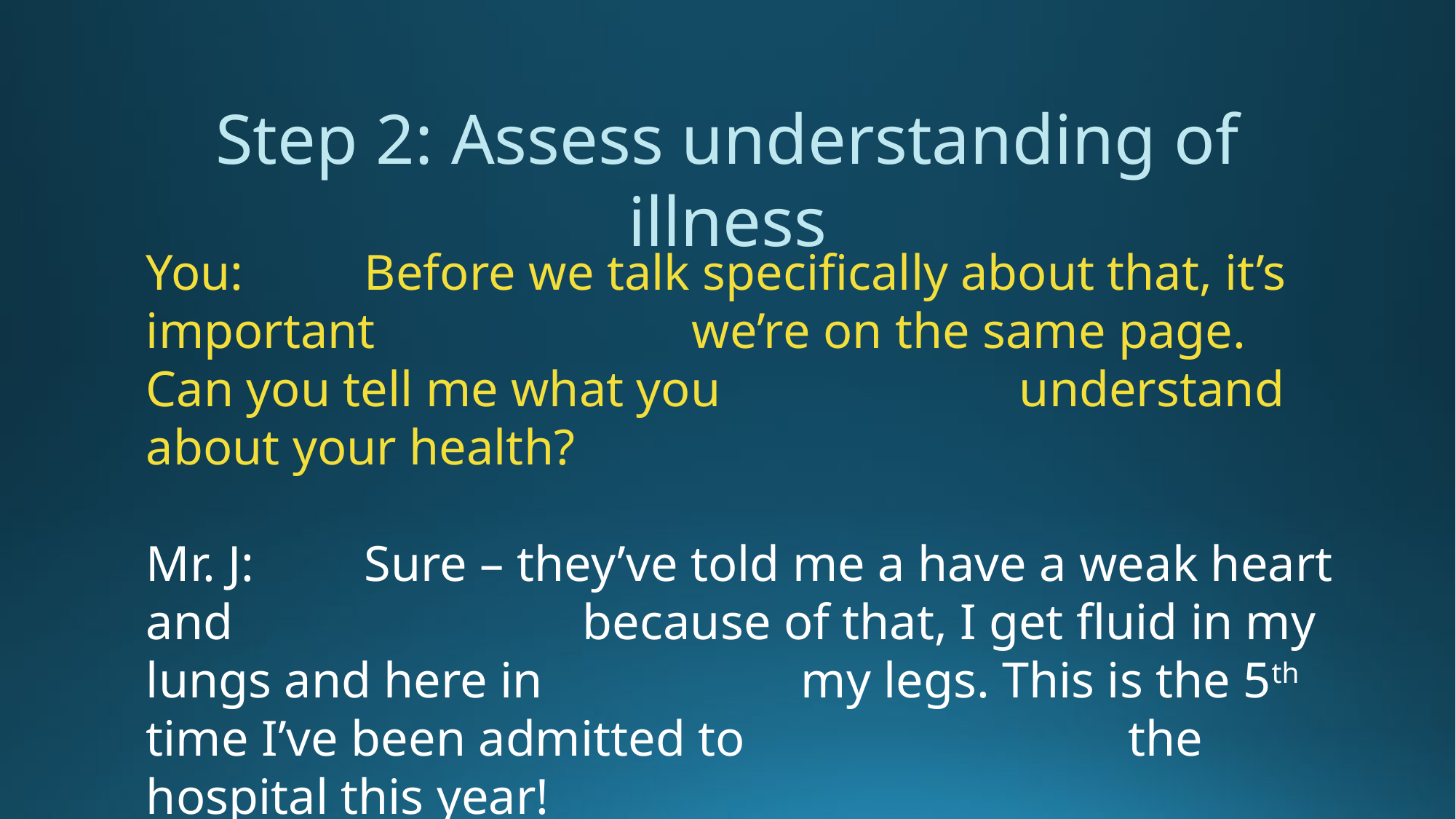

Step 2: Assess understanding of illness
You:		Before we talk specifically about that, it’s important 			we’re on the same page. Can you tell me what you 			understand about your health?
Mr. J:		Sure – they’ve told me a have a weak heart and 				because of that, I get fluid in my lungs and here in 			my legs. This is the 5th time I’ve been admitted to 				the hospital this year!

## Slide 32
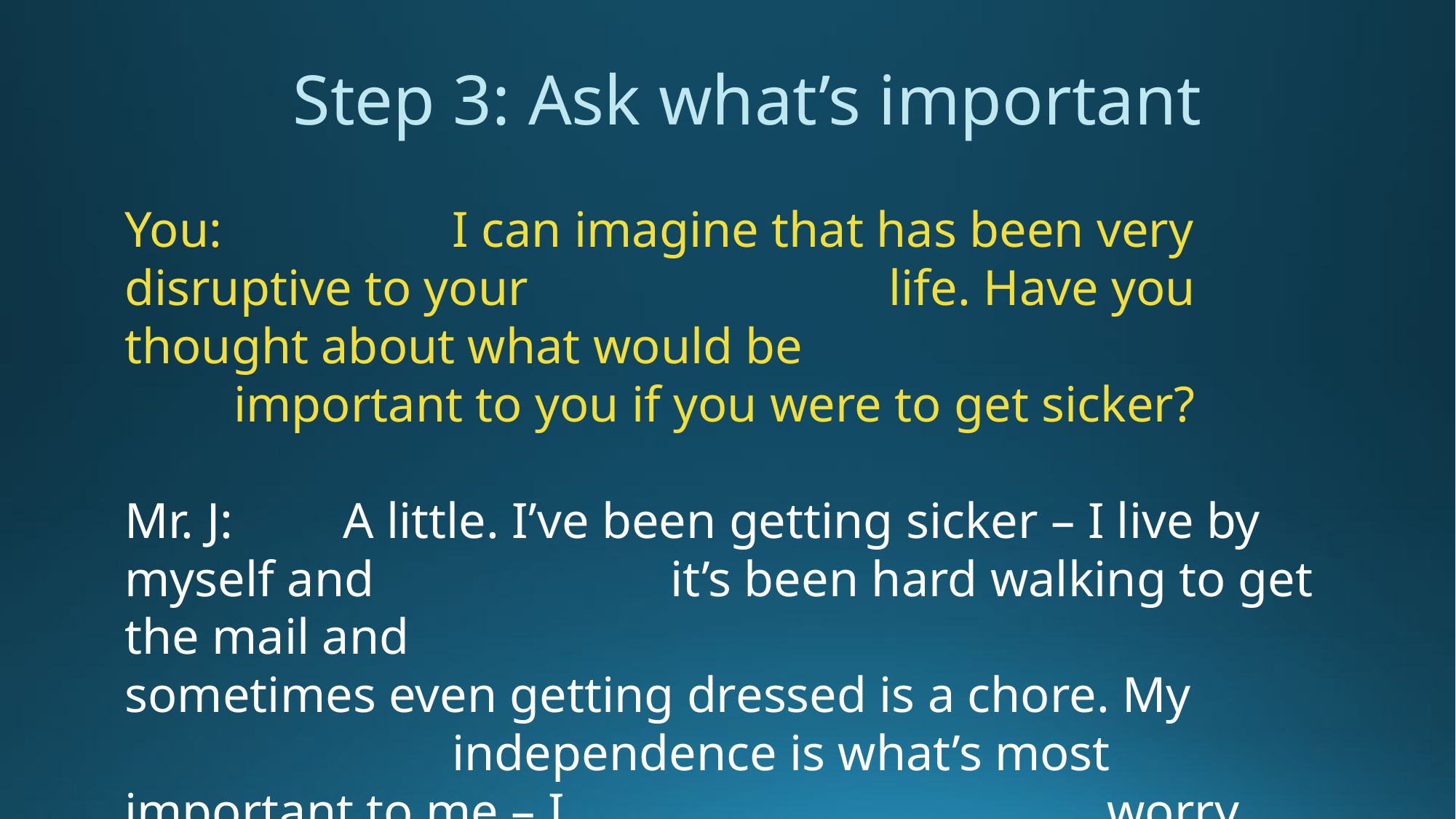

Step 3: Ask what’s important
You: 		I can imagine that has been very disruptive to your 				life. Have you thought about what would be 						important to you if you were to get sicker?
Mr. J: 	A little. I’ve been getting sicker – I live by myself and 			it’s been hard walking to get the mail and 							sometimes even getting dressed is a chore. My 					independence is what’s most important to me – I 					worry that’s starting to go away.

## Slide 33
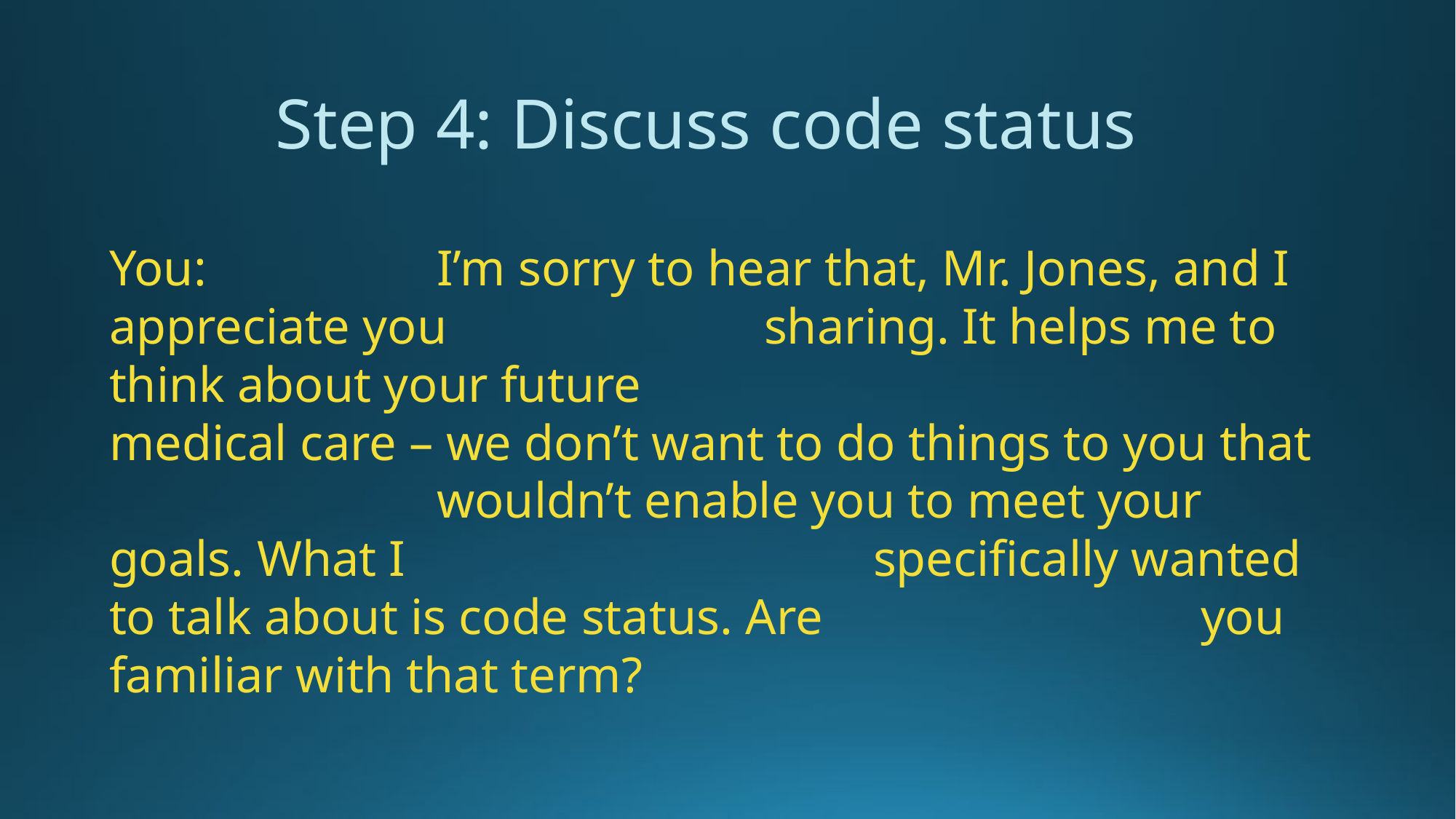

Step 4: Discuss code status
You: 		I’m sorry to hear that, Mr. Jones, and I appreciate you 			sharing. It helps me to think about your future 						medical care – we don’t want to do things to you that 			wouldn’t enable you to meet your goals. What I 					specifically wanted to talk about is code status. Are 				you familiar with that term?

## Slide 34
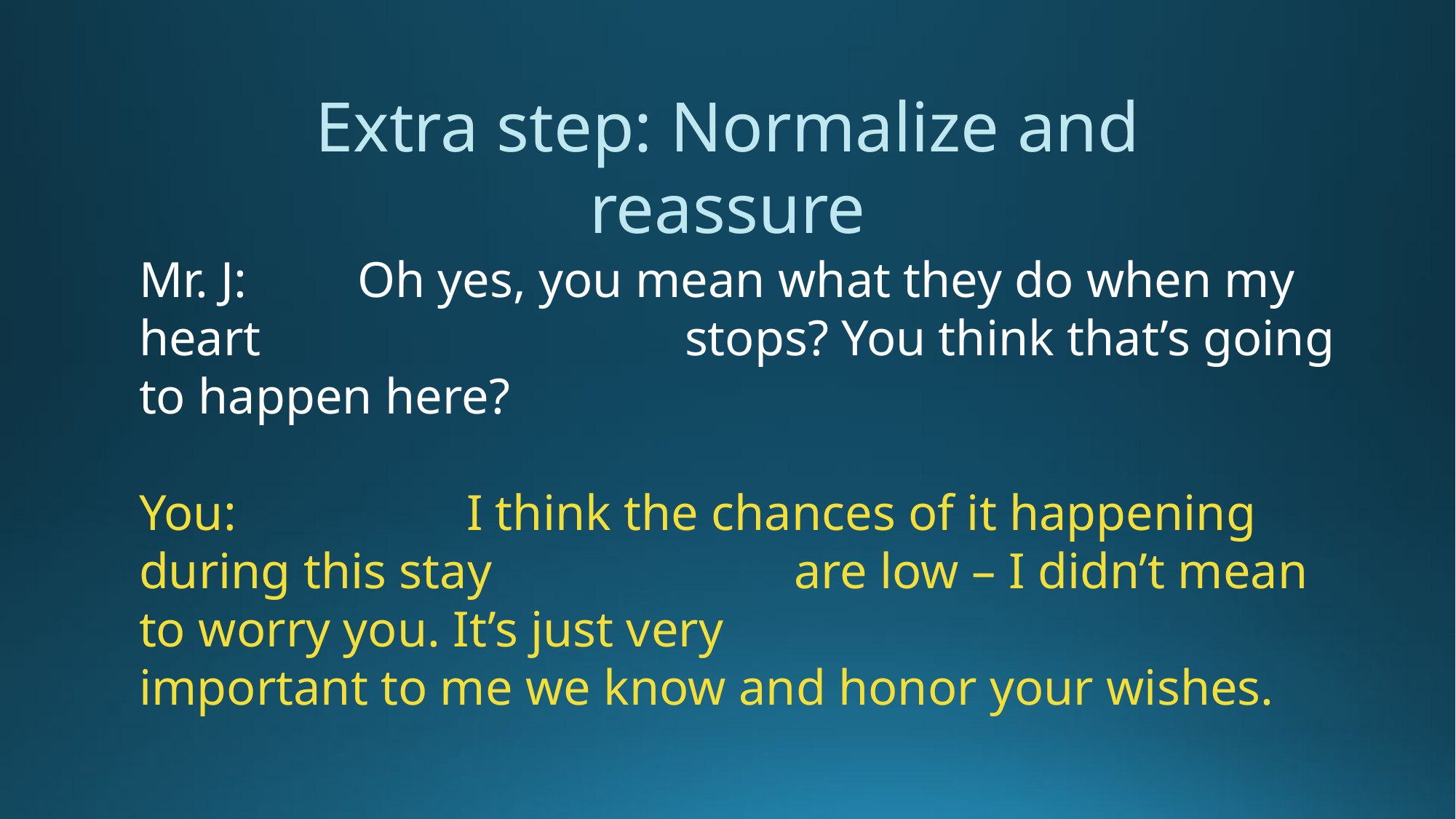

Extra step: Normalize and reassure
Mr. J: 	Oh yes, you mean what they do when my heart 				stops? You think that’s going to happen here?
You: 		I think the chances of it happening during this stay 			are low – I didn’t mean to worry you. It’s just very 				important to me we know and honor your wishes.

## Slide 35
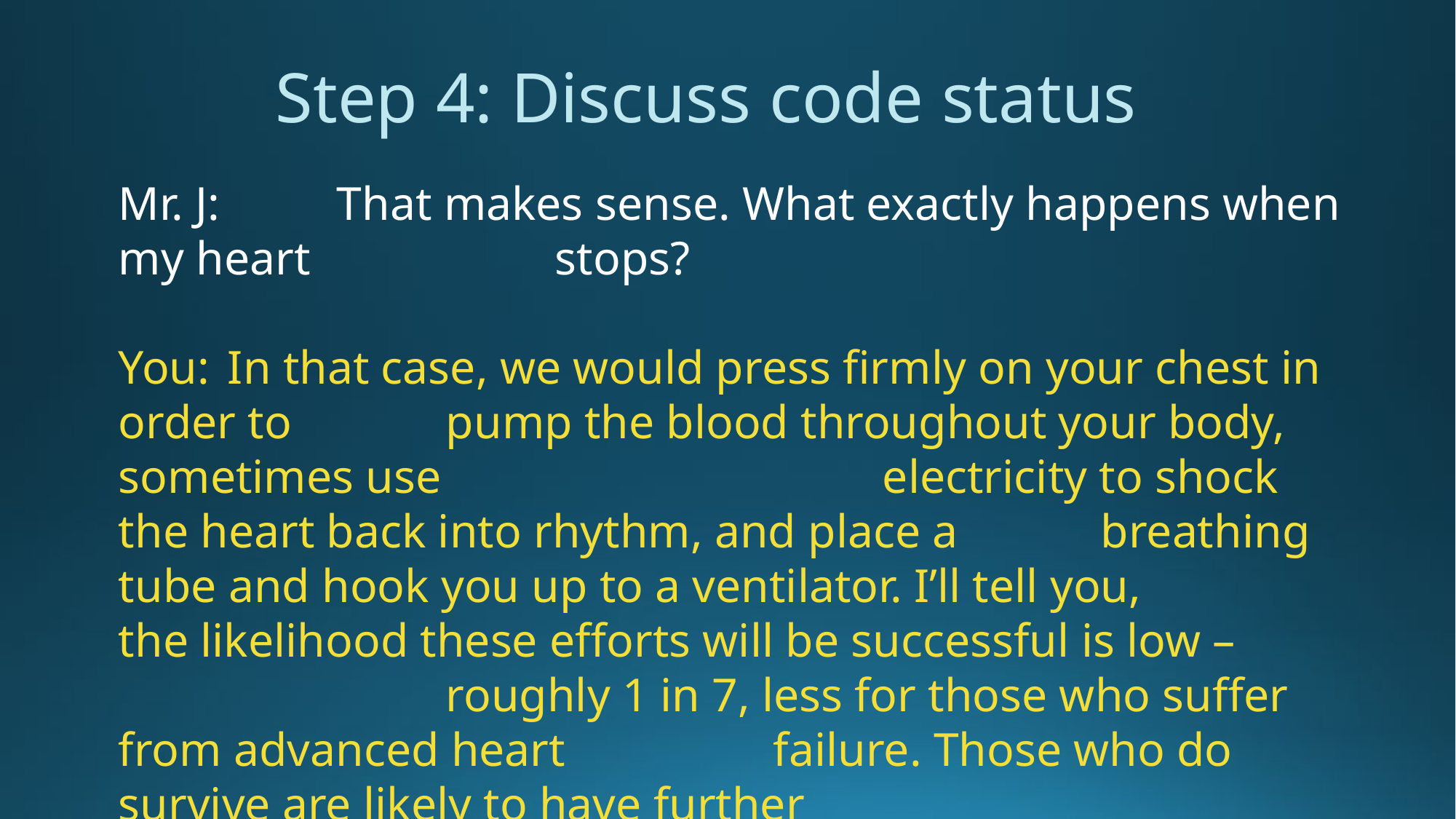

Step 4: Discuss code status
Mr. J: 	That makes sense. What exactly happens when my heart 			stops?
You: 	In that case, we would press firmly on your chest in order to 		pump the blood throughout your body, sometimes use 				electricity to shock the heart back into rhythm, and place a 		breathing tube and hook you up to a ventilator. I’ll tell you, 		the likelihood these efforts will be successful is low – 				roughly 1 in 7, less for those who suffer from advanced heart 		failure. Those who do survive are likely to have further 				disability.

## Slide 36
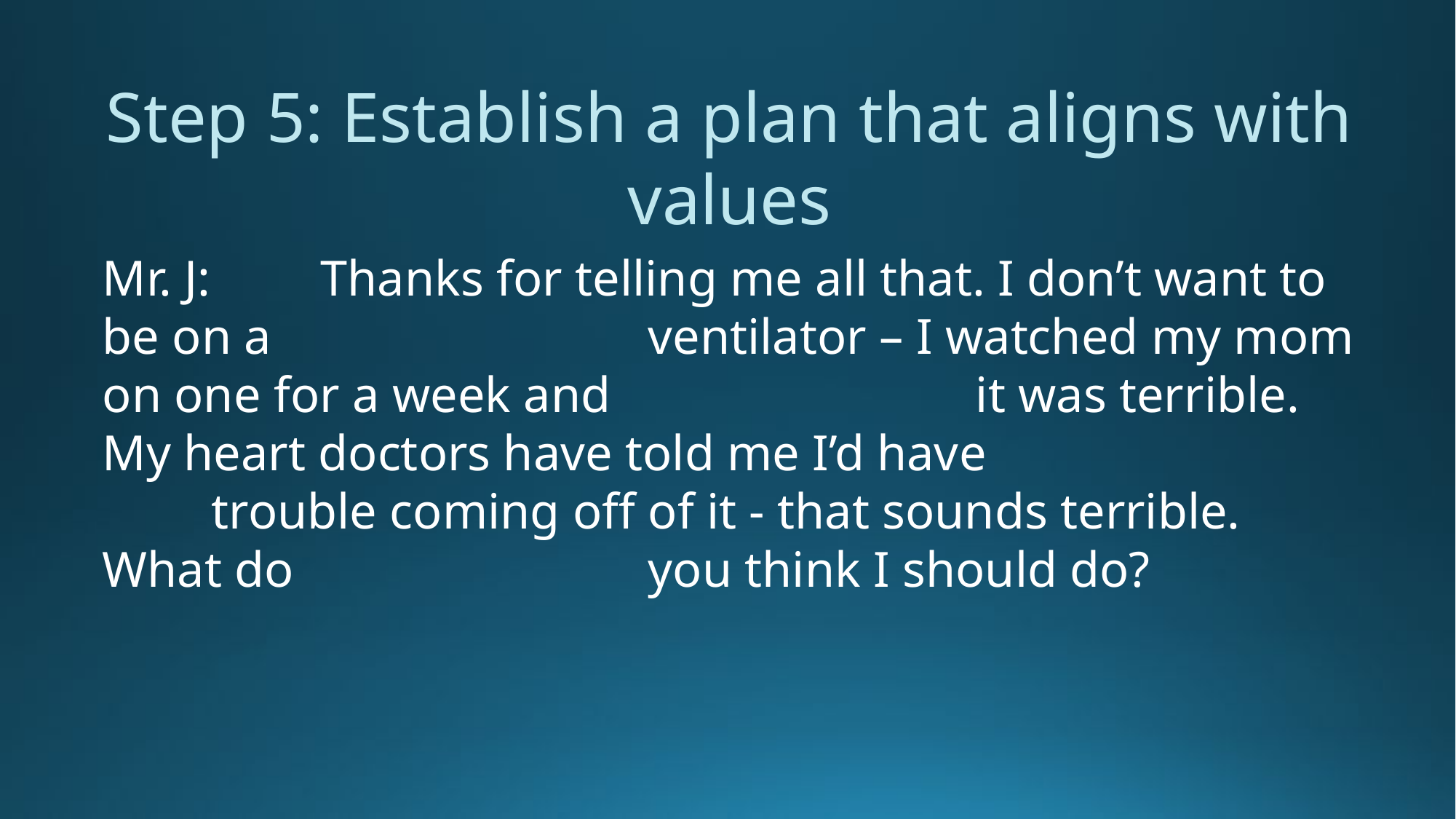

Step 5: Establish a plan that aligns with values
Mr. J: 	Thanks for telling me all that. I don’t want to be on a 				ventilator – I watched my mom on one for a week and 				it was terrible. My heart doctors have told me I’d have 				trouble coming off of it - that sounds terrible. What do 				you think I should do?

## Slide 37
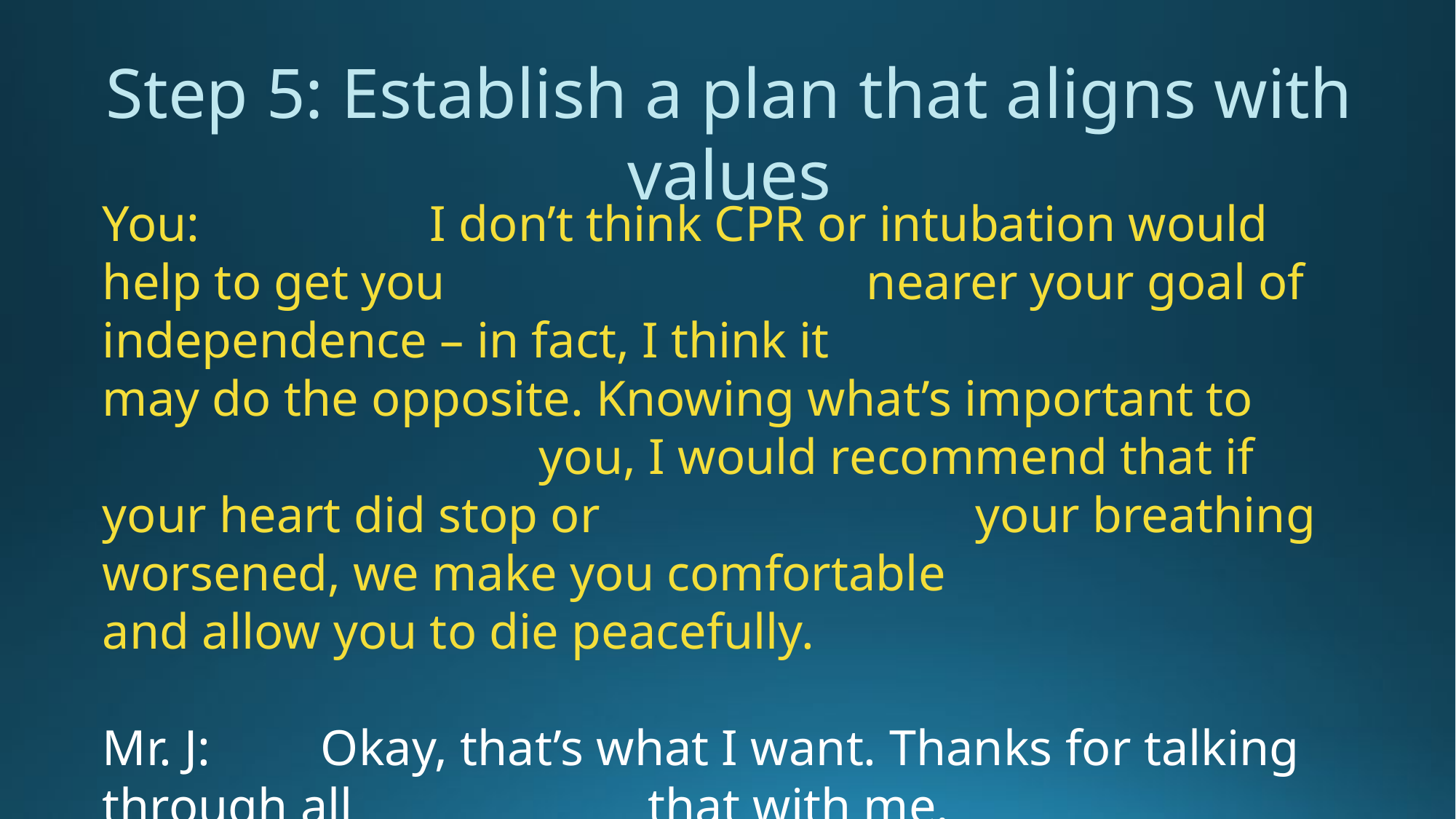

Step 5: Establish a plan that aligns with values
You: 		I don’t think CPR or intubation would help to get you 				nearer your goal of independence – in fact, I think it 					may do the opposite. Knowing what’s important to 					you, I would recommend that if your heart did stop or 				your breathing worsened, we make you comfortable 				and allow you to die peacefully.
Mr. J:	 	Okay, that’s what I want. Thanks for talking through all 			that with me.

## Slide 38
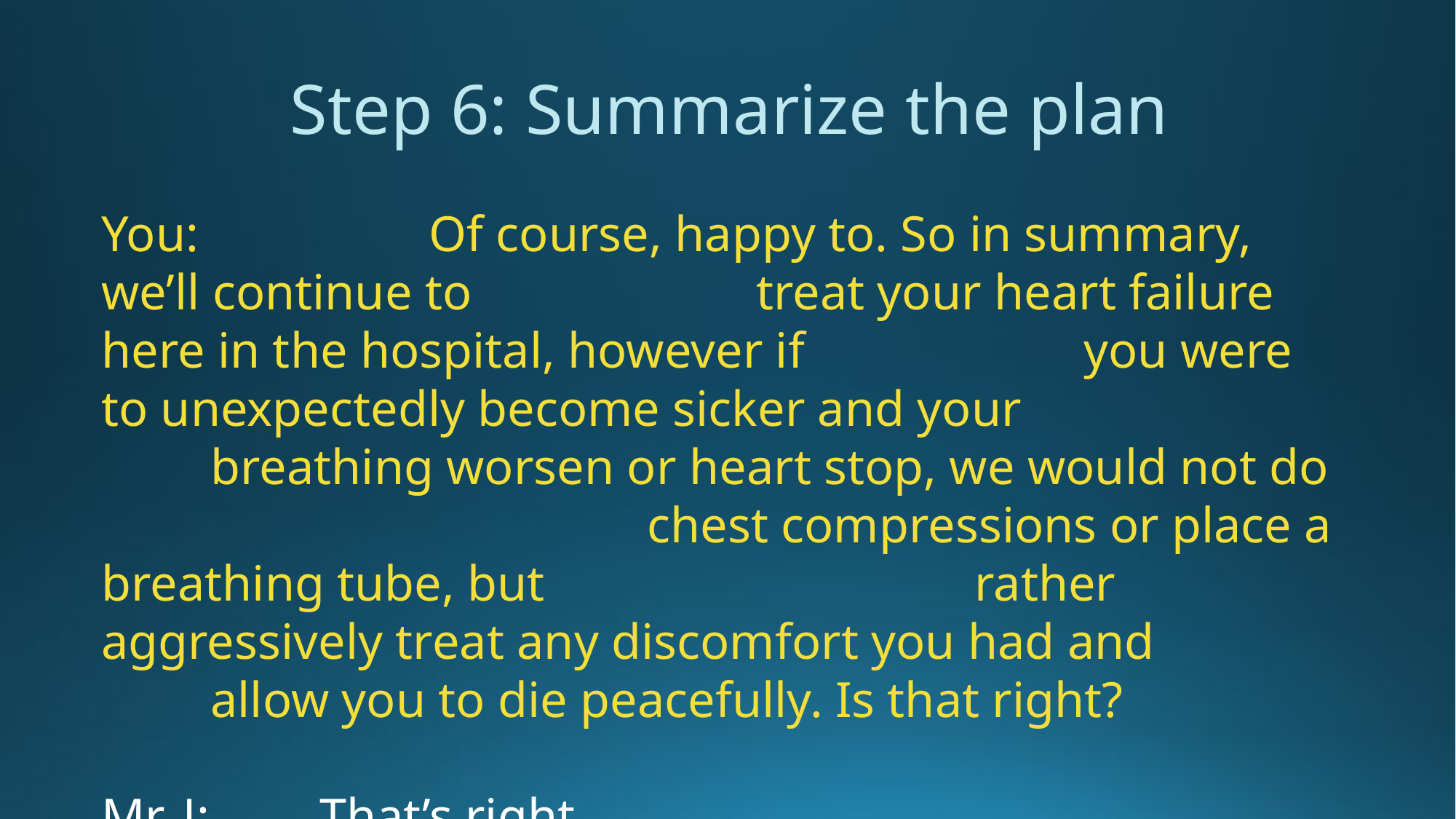

Step 6: Summarize the plan
You: 		Of course, happy to. So in summary, we’ll continue to 			treat your heart failure here in the hospital, however if 			you were to unexpectedly become sicker and your 				breathing worsen or heart stop, we would not do 					chest compressions or place a breathing tube, but 				rather aggressively treat any discomfort you had and 			allow you to die peacefully. Is that right?
Mr. J: 	That’s right.

## Slide 39
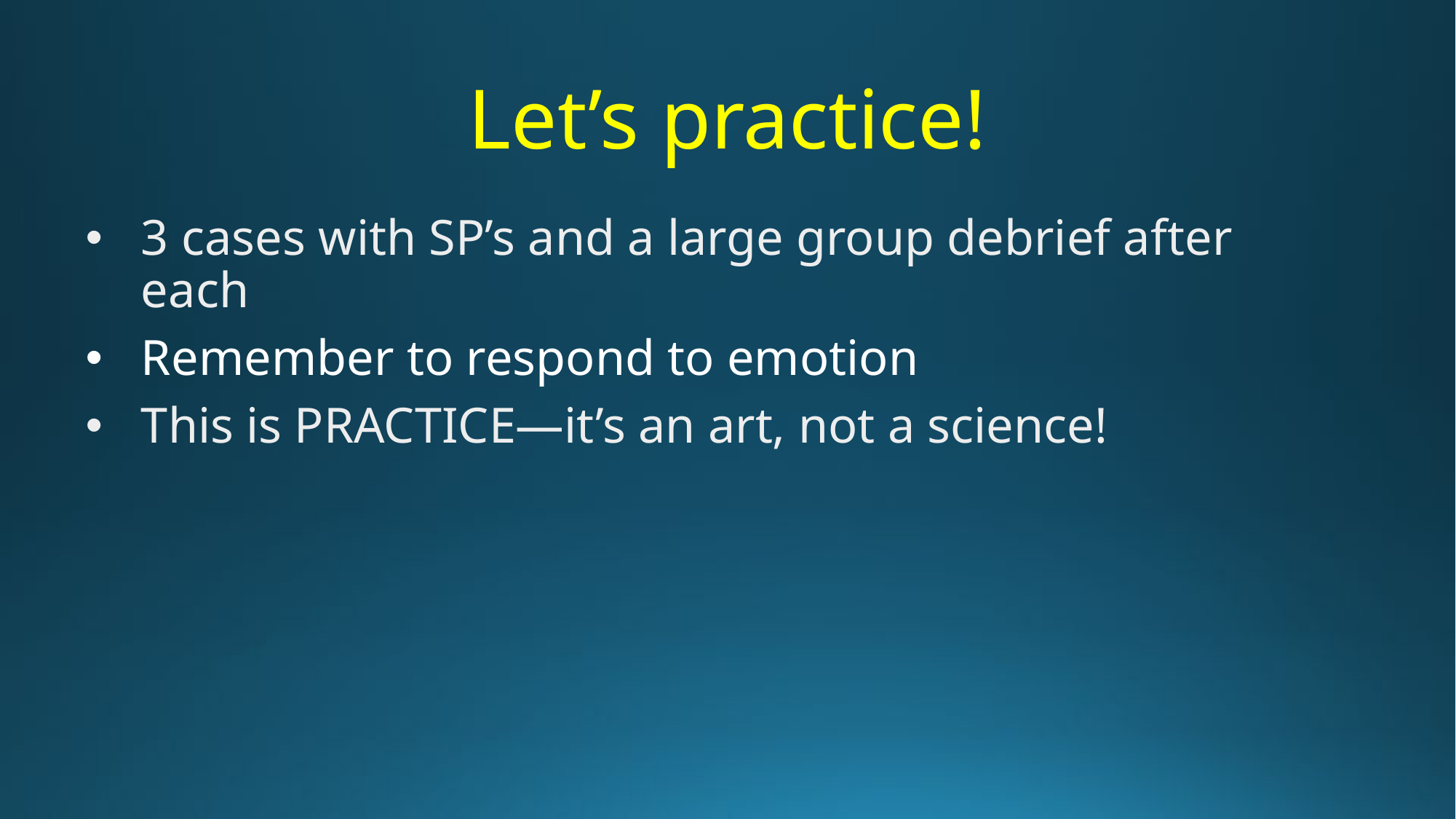

# Let’s practice!
3 cases with SP’s and a large group debrief after each
Remember to respond to emotion
This is PRACTICE—it’s an art, not a science!

## Slide 40
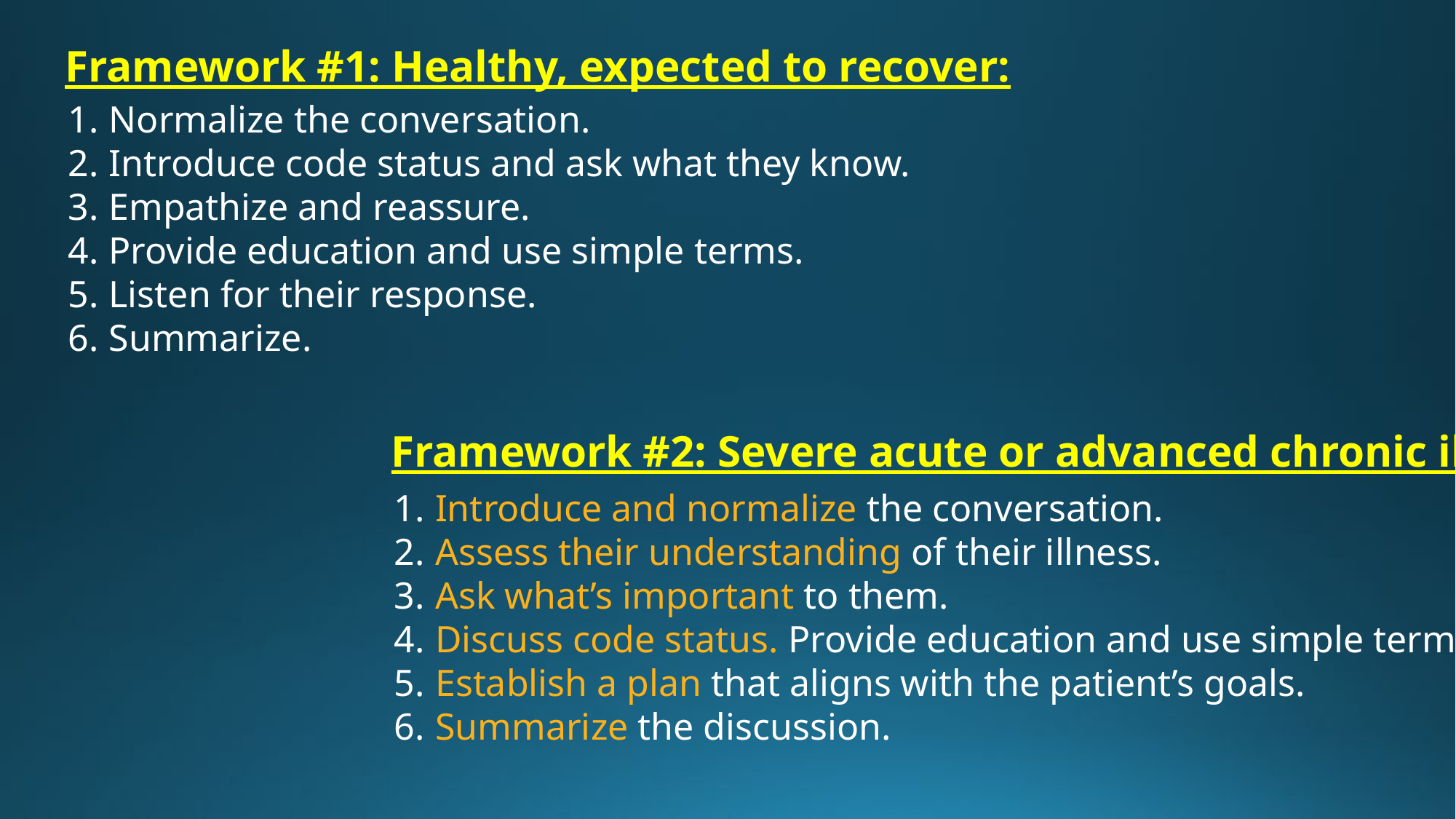

Framework #1: Healthy, expected to recover:
Normalize the conversation.
Introduce code status and ask what they know.
Empathize and reassure.
Provide education and use simple terms.
Listen for their response.
Summarize.
Framework #2: Severe acute or advanced chronic illness:
Introduce and normalize the conversation.
Assess their understanding of their illness.
Ask what’s important to them.
Discuss code status. Provide education and use simple terms.
Establish a plan that aligns with the patient’s goals.
Summarize the discussion.

## Slide 41
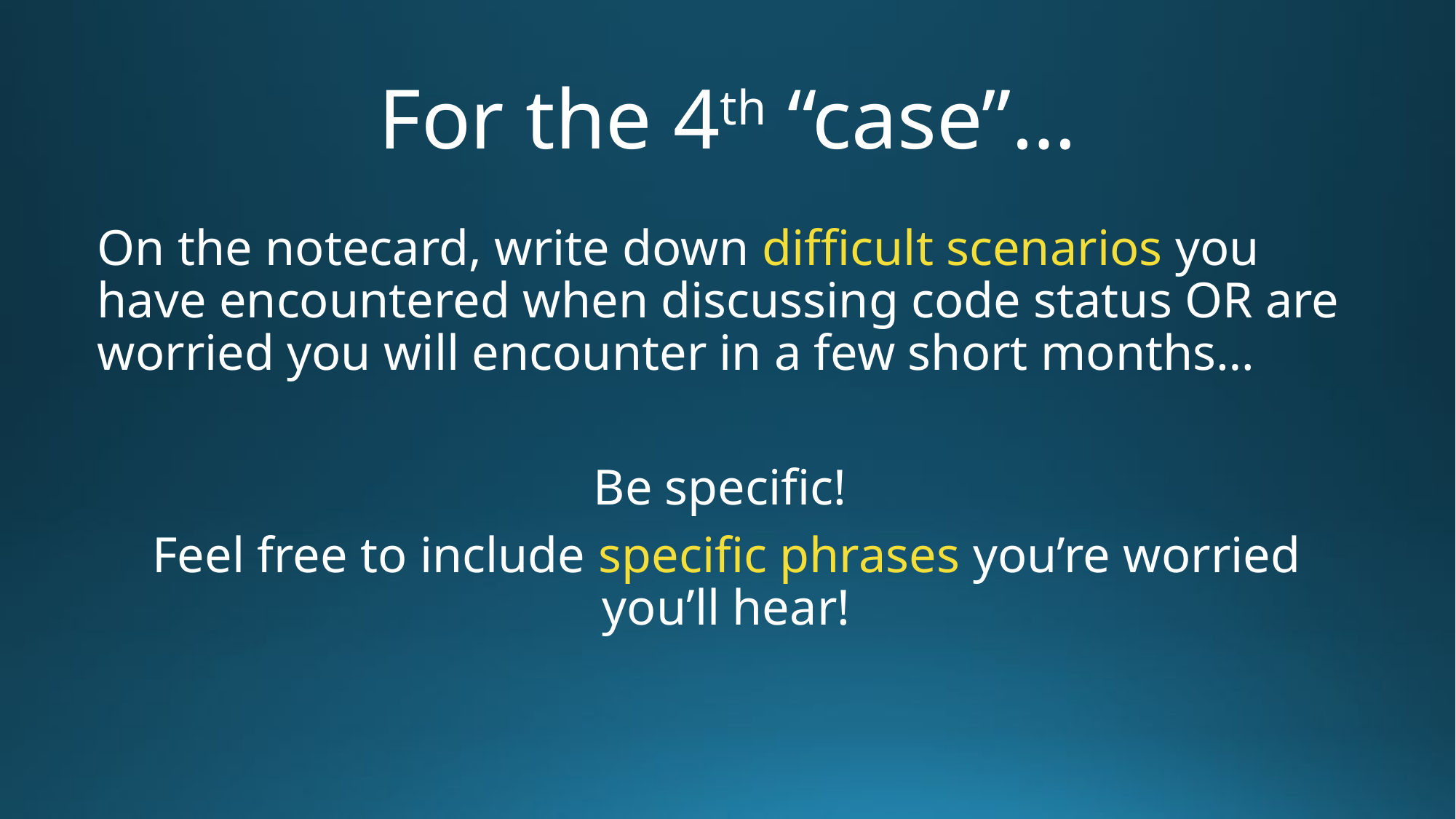

# For the 4th “case”…
On the notecard, write down difficult scenarios you have encountered when discussing code status OR are worried you will encounter in a few short months…
Be specific!
Feel free to include specific phrases you’re worried you’ll hear!

## Slide 42
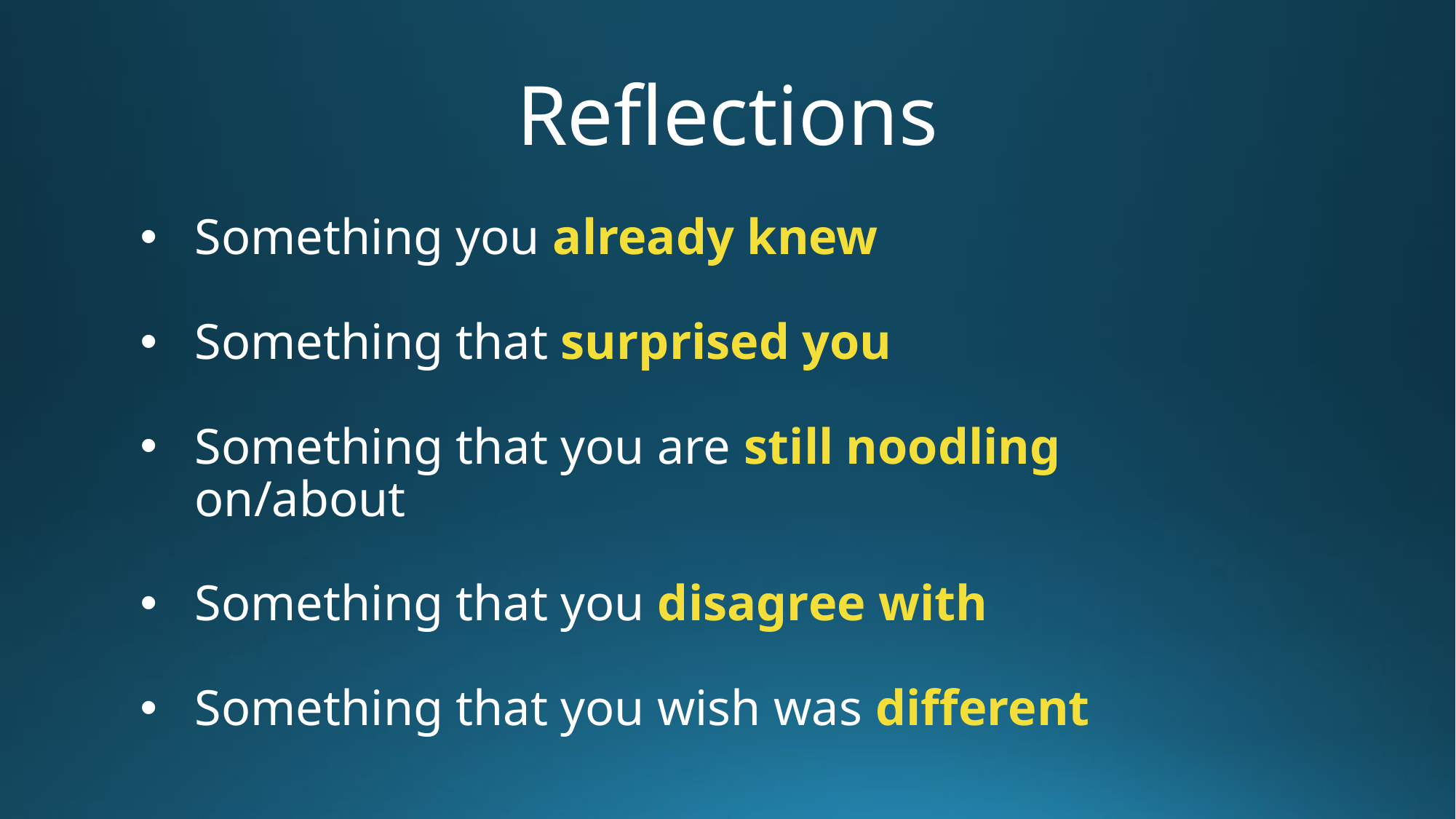

# Reflections
Something you already knew
Something that surprised you
Something that you are still noodling on/about
Something that you disagree with
Something that you wish was different
