# Supplementary material for: Code Status Discussions: A Standardized Patient Workshop for Senior Medical Students
Source: MedEdPORTAL. 2025 Sep 2;21:11546. doi: 10.15766/mep_2374-8265.11546 (PMC12402213; doi:10.15766/mep_2374-8265.11546)
Supplement: Supplementary file 1 — Didactic.pptxStudent Case Handouts.docxFacilitator Guide.docxWorkshop Frameworks Handouts.docxPre- and Postworkshop Survey.docxSP Guide.docx [file mep_2374-8265.11546-s001.zip › B. Student Case Handouts.docx]

**Medicine Scenario #1: Discussing Code Status on Admission**

Intern:

You are admitting Mr. / Ms. Reed, a 63yo with RUQ pain, fever, and leukocytosis, found to have acute cholecystitis. They are being admitted for IV fluids, pain control, and antibiotics – your upper level has finished placing the orders. The patient is now afebrile and appears comfortable. You wish to discuss code status.

**Medicine Scenario #2: Discussing Code Status in the Clinic**

Intern:

You are seeing one of your primary care clinic patients, Mr. / Ms. Smith, for follow-up. During their last visit 2 weeks ago, you reviewed the unfortunate news they received from oncology that their metastatic melanoma has progressed through the most recent line of treatment and unfortunately, there are no more cancer-directed treatment options available. You scheduled this visit today as a general check-in. Now that Mr. / Ms. Smith has had time to process this news, your attending asks you to discuss code status with them.

**Medicine Scenario #3 – Discussing Code Status with Clinical Worsening while Inpatient**

Intern:

You are the intern in the ICU caring for Mr./Mrs. Baker, who is a 38yo with a history of substance use disorder admitted after an unintentional heroin overdose, at which time they suffered a cardiac arrest. They have unfortunately remained minimally responsive without sedation and now, on day 9 of admission, have developed a ventilator-associated pneumonia. Throughout their course, you have spoken with their spouse about what Mr./Ms. Baker values — their sense of humor, being active, time with family, and their independence. They are now in septic shock and requiring three pressors. Your team met with their spouse earlier in the day and delivered the unfortunate news that given the severity of their infection now with multi-organ failure, you worry Mr./Mrs. Baker is going to die. After giving their spouse some time to process this, your upper level asks you to address code status.

**Peds Scenario #1: Discussing Code Status in the NICU**

Intern:

You are caring for Jade, a 10-day-old full term baby girl with a skeletal dysplasia. As part of her condition, she has a small thorax with abnormal ribs. Her family was prepared that she would very likely require respiratory support and were accepting of intubation if needed. She is now on the ventilator and clinically stable, though you know her hospitalization is likely to be prolonged and perhaps complicated. You have come to know her family over the past few days and after providing medical updates, wish to discuss code status.

**Peds Scenario #2: Discussing Code Status in the Clinic**

Intern:

You are rotating through the neurology clinic and are now seeing TJ, an 18-month-old with SMA type 1. SMA is *spinal muscle atrophy*, a neuromuscular disorder characterized by progressive muscle weakness, type 1 being most severe and typically associated with death by 2 years of age due to respiratory failure. TJ is diffusely weak, minimally able to move his body. His parents have noticed his cough is weak and he has begun to lose weight, unable to take sufficient oral nutrition. Given his progressive decline, your attending wishes for you to discuss what parents may prefer for TJ’s medical treatment in the future. They specifically ask that you discuss code status.

**Peds Scenario #3: Discussing Code Status with Clinical Decline While in the PICU**

Intern:

You are the intern caring for Luke, a 15yo M with history of refractory AML admitted with complications related to bone marrow transplant. He has been in the hospital for 2 months and is now bacteremic with septic shock. He is intubated and is requiring maximal pressor support. Throughout his course, you have spoken with his parents about what makes Luke himself – his sense of humor, his love of family, his independence – all things he values deeply. You meet with his parents and deliver the unfortunate news that given the severity of his infection, you worry Luke will die. After giving them some time to process this news, your upper level asks you to return to discuss code status.

**MP/FM Scenario #1: Discussing Code Status on Admission**

Intern:

You are admitting Mr. / Ms. Reed, a 63yo with RUQ pain, fever, and leukocytosis, found to have acute cholecystitis. They are being admitted for IV fluids, pain control, and antibiotics – your upper level has finished placing their orders. They are now afebrile and appear comfortable. You wish to discuss code status.

**MP/FM Scenario #2: Discussing Code Status in the Clinic**

Intern:

You are rotating through the neurology clinic and are now seeing TJ, an 18-month-old with SMA type 1. SMA is *spinal muscle atrophy*, a neuromuscular disorder characterized by progressive muscle weakness, type 1 being most severe and typically associated with death by 2 years of age due to respiratory failure. TJ is diffusely weak, minimally able to move his body. His parents have noticed his cough is weak and he has begun to lose weight, unable to take sufficient oral nutrition. Given his progressive decline, your attending wishes for you to discuss what parents may prefer for TJ’s medical treatment in the future. They specifically ask that you discuss code status with his parent.

**MP/FM Scenario #3: Discussing Code Status with Clinical Decline While in the PICU**

Intern:

You are the intern caring for Luke, a 15yo M with history of refractory AML admitted with complications related to bone marrow transplant. He has been in the hospital for 2 months and is now bacteremic with septic shock. He is intubated and is requiring maximal pressor support. Throughout his course, you have spoken with his parents about what makes Luke himself – his sense of humor, his love of family, his independence – all things he values deeply. You meet with his parents and deliver the unfortunate news that given the severity of his infection, you worry Luke will die. After giving them some time to process this news, your upper level asks you to return to discuss code status.

**OB/GYN Scenario #1: Discussing Code Status on Admission**

Intern

Intern: You are admitting Ms. Reed, a nulliparous 63-year-old presenting with abnormal uterine bleeding. She is being admitted for overnight observation after receiving 1 unit packed red blood cell transfusion for a hemoglobin of 6.8. She has a known history of iron deficiency anemia with a baseline hemoglobin of 7.5, and she has been hemodynamically stable since arrival to the ER today. Your upper level is placing her orders and you are tasked with discussing her code status.

**OB/GYN Scenario #2: Discussing Code Status with Worsening Prognosis**

Intern:

You are admitting Ms. Root, a 58 year-old with known stage III ovarian cancer presenting to the hospital from the GYN/ONC clinic with failure to thrive. She was diagnosed 4 years ago and has received several rounds of chemotherapy. She initially responded well to therapy but her latest CMP revealed markedly elevated ALP and transaminases concerning for extension of her disease into the liver. CT in the ED confirmed new liver metastases. You are admitting her to the GYN/ONC inpatient service and now will discuss her code status. She has previously been full code.

**OB/GYN Scenario #3: Discussing Code Status with Clinical Worsening while Inpatient**

Intern:

You are the intern caring for Ms. Road, a 70 yo F who presented with a saddle pulmonary embolism (PE) and was found to have a new diagnosis of high-grade endometrial carcinoma. Her PE was complicated by cardiac arrest for which she received chest compressions, was intubated, and required management in the cardiac ICU. After extubation, she was transferred to the GYN/ONC team for further work-up of her abnormal uterine bleeding. Biopsy revealed high grade endometrial carcinoma and she was not offered surgery due to the extent of her disease. She continues to have vaginal bleeding that requires frequent blood transfusions and, unfortunately, her kidneys are beginning to fail. Due to her organ dysfunction and poor performance status, she is not currently a candidate for chemotherapy, but is receiving palliative radiation to control bleeding. She has been disturbed by the recent events, but believes she can recover if she receives chemotherapy. Your team is worried that she may soon require ICU-level care and that she is at high risk for having another cardiac arrest. Your upper level asks you to address code status.

**Surgery Scenario #1: Discussing Code Status on Hospital Admission**

Intern:

You are admitting Mr. / Ms. Reed, a 63yo with RUQ pain, fever, and leukocytosis, found to have acute cholecystitis. They are being admitted for IV fluids, pain control, and antibiotics – your upper level has finished placing the orders. The patient is now afebrile and appears comfortable. You wish to discuss code status.

**Surgery Scenario #2: Discussing Code Status Prior to Surgery**

Intern:

Mr./Ms. Smith is an 85yo with history of hypertension admitted now with left hip fracture after sustaining a fall at their assisted living facility. Their spouse passed away several years ago and, due to mounting debility, Mr./Mrs. Smith previously made the decision to be DNR/DNI, stating “when it’s my time to go, I want to go”. Due to pain, they are wishing to undergo fracture repair. Your upper level asks that you discuss with Mr./Ms. Smith’s code status in the context of their upcoming surgery.

**Surgery Scenario #3: Clinical Worsening While in the SICU**

Intern:

Mr./Ms. Lee is a 65yo with a history of T2DM admitted two weeks ago with urosepsis and profound hypotension. Hemodynamics have improved, but they remain intubated due to weakness. Three days ago, they developed abdominal distension with intolerance of NG tube feeds. CT of the abdomen demonstrated bowel wall edema. They were taken to the OR where they were found to have bowel necrosis and underwent small bowel resection. They returned to the SICU with open abdomen. Unfortunately, over the past 24 hours, their clinical condition has deteriorated. Re-look of the abdomen reveals extensive necrosis of the remaining bowel not amenable to surgical intervention. You meet with the spouse and deliver the unfortunate news that given the severity of Mr./Ms. L’s illness, you believe they will not survive. After giving their spouse some time to process this news, you wish to discuss code status.
