# Supplementary material for: Code Status Discussions: A Standardized Patient Workshop for Senior Medical Students
Source: MedEdPORTAL. 2025 Sep 2;21:11546. doi: 10.15766/mep_2374-8265.11546 (PMC12402213; doi:10.15766/mep_2374-8265.11546)
Supplement: Supplementary file 1 — Didactic.pptxStudent Case Handouts.docxFacilitator Guide.docxWorkshop Frameworks Handouts.docxPre- and Postworkshop Survey.docxSP Guide.docx [file mep_2374-8265.11546-s001.zip › _Educational Summary Report_11546.pdf]

MedEdPORTAL® The AAMC Journal of Teaching and Learning Resources

Original Publication Open Access

Code Status Discussions: A Standardized Patient Workshop for Senior Medical Students

Hunter Bechtold, MD*, Reagan T. Smith, Peter Wallenhorst, MD, Kaylee Gouge, MD, MPH, Sarah Schaefer, MD, Kelsey Karnik, PhD, Kristen McQuerry, PhD, Kristen E. Fletcher, MD

*Corresponding author: hunter.bechtold@uky.edu

Abstract

Introduction: Residents frequently conduct code status discussions (CSDs) with patients, but many report not receiving formal training in this skill. While institutions have attempted to address this, there remains a need for a curriculum that uses standardized patients (SPs), is generalizable to students interested in different medical specialties, and has been tested on a large sample of students. **Methods:** We trained 192 fourth-year medical students across four different campuses in how to conduct CSDs during one 2-hour workshop as part of their Transition to Residency course in April 2024. Students worked with SPs as part of this workshop. Students at our main campus completed pre- and postworkshop paper surveys evaluating their confidence levels across five domains. **Results:** A total of 84 (69%) of the 121 students at the main campus completed both a pre- and postworkshop survey. There was a statistically significant change in confidence levels from pre- to postworkshop, with a higher proportion of students selecting increased confidence levels across all five questions on the postworkshop survey. **Discussion:** Our workshop increased the confidence of fourth-year medical students in conducting CSDs. This session successfully implemented simulations that used SPs for training a large group of fourth-year medical students bound for different medical specialties. This is the first workshop of its size and breadth, and it may serve as a standardized curriculum for other institutions wishing to formally train medical students to conduct CSDs.

Keywords
Code Status Discussion, Transition to Residency, Standardized Patient, Communication Skills, End of Life/Palliative Care

Educational Objectives

By the end of this activity, learners will be able to:

1. Identify when code status should be addressed.
2. Implement a framework for code status discussions.
3. Identify the role of code status discussions in overall care.

Introduction

Code status discussions (CSDs), the process in which clinicians obtain a patient's preferences regarding life-sustaining interventions, are essential in clinical practice. CSDs are high-stakes, stressful communications and occur commonly at pivotal junctures of care for patients.[1] Resident physicians frequently lead CSDs, although they commonly lack prior formal training.[2-5]

Citation:
Bechtold H, Smith RT, Wallenhorst P, et al. Code status discussions: a standardized patient workshop for senior medical students. *MedEdPORTAL*. 2025;21:11546.
https://doi.org/10.15766/mep_2374-8265.11546

Despite the importance of knowing how to lead CSDs early in one's career, there is no standardized curriculum for teaching this skill to medical trainees. A 2005 literature review by Gorman and colleagues highlighted that many residents feel that they are inadequately trained for providing end-of-life care, including CSDs.[6] Multiple studies since then have emphasized this gap, including studies highlighting that residents across specialties and institutions report not receiving formal education in medical school, and that many lack confidence in conducting CSDs.[4,5,7,8]

Several institutions have attempted to address this lack of formal CSD training. Studies evaluating CSD education have demonstrated success through varied approaches at different training levels. Prior curricula have included learners ranging from second-year medical students to resident trainees and have varied in pedagogy. Boot camp-style programs for graduating medical students have utilized didactic sessions followed by standardized patient (SP) simulations, showing improved CSD performance compared to untrained interns.[9] Preclinical medical school interventions have enhanced awareness and confidence through preliminary reading materials, didactics,

Copyright © 2025 Bechtold et al. This is an open-access publication distributed under the terms of the Creative Commons Attribution license.

and nonstandardized role-play.[10] Published resident curricula have focused on guideline-based approaches and frameworks, including resuscitation outcome education and risk-benefit analysis, with participants reporting increased comfort and practical knowledge.[11] Despite the variety of approaches, overall evidence suggests that SP involvement and proximity to residency are particularly effective.[12-14]

Improved resident physician confidence and skill can benefit both the resident leading the conversation and the patient engaging in the conversation. For the resident, improved confidence surrounding CSDs has been shown to decrease trainee stress response in high-stakes communications, including CSDs.[1] This empowers residents to engage in such communication without the aid of palliative medicine specialists. Higher self-efficacy of physicians delivering serious news has been associated with lower risk of burnout.[15] Conversely, in one study, objectively poor communication performance in a high-stakes communication simulation scenario was associated with high depersonalization and fatigue levels among the physicians involved.[16] Thus, CSD training may reduce trainee stress and mitigate burnout by improving confidence in high-stakes communication.

Patients also benefit from physicians skilled in CSD. Yet, a recent study found that 61% of admitted patients did not recall having a CSD during their hospitalization; of those who did recall a discussion, there was a discordance between the patient's perception of resuscitation decisions and what was documented in the electronic medical record.[17] CSD training is likely to increase the frequency of discussions due to greater trainee comfort, and will hopefully lessen discordance by enabling clearer discussions with a conclusive summary statement regarding the patient's wishes.[18] Furthermore, training in CSDs has shown that physicians are more likely to make recommendations regarding code status, a core tenet of CSD.[19]

Given the heterogeneity of published curricula, we sought to create a CSD workshop leveraging didactics and SPs in varied clinical settings using a just-in-time instructional approach. We scheduled the workshop during the Transition to Residency (TTR) course, taught during the final semester of medical school, to optimize retention and application of CSD concepts during internship. We chose this approach to equip graduating students with the required skills they will ideally employ within the subsequent 6 months of their training, a learning approach widely used in various simulation settings.[20] We also elected to engage in this approach based on its well-established and wide use in medical education, particularly for training communication skills. Incorporation of SPs into the simulations allows students to practice leading difficult conversations, such as CSDs, in a lower-stakes environment and can improve learner confidence.[13] To our knowledge, this is one of the first published workshops centered around obtaining a code status utilizing SPs for specialty-specific case scenarios in the just-in-time TTR curriculum.

Methods

Course Design

We designed this workshop to help senior medical students identify when CSDs are appropriate and practice empathic communication skills across a range of clinical scenarios with varying patient prognoses. Two fellowship-trained, board-certified hospice and palliative medicine physicians developed this workshop by drawing on their experiences supervising interns during code status and goals-of-care conversations. In collaboration with a TTR co-course director, we met monthly for 6 months to refine the pedagogy. Ultimately, we chose a format that combines a brief didactic introduction with a case-based SP workshop, where students practice communication skills in small groups, followed by large-group debriefs.

The University of Kentucky Institutional Review Board deemed this project exempt from review (case 90880; February 22, 2024).

Setting

We delivered this workshop in April 2024 during our TTR course at the University of Kentucky College of Medicine. The College has one main campus, two regional campuses, and one regional site. Each graduating student completed this workshop during TTR. However, we present here the data from the main campus only, as we evaluated learners only at the main campus. The main campus was prioritized due to personnel resource limitations and given that most students (121 of 192) attended this campus.

The students within the course were asked to choose the track that most closely resembles their future residency specialty from the following options: adult medicine, family medicine/medicine-pediatrics, pediatrics, OB/GYN, and surgery/trauma. The students were organized by their track, and at the main campus, student group sizes ranged from as few as four students up to 35 students. Though the cases presented to each track were written to resemble the unique demographic of patients for that track, each workshop followed the same general format.

Workshop Overview

The workshop consisted of a didactic session (Appendix A) followed by small-group practice with SPs. Ideally, student prerequisite experience would include having seen at least

Copyright © 2025 Bechtold et al. This is an open-access publication distributed under the terms of the Creative Commons Attribution license.

one code status conversation during their clinical experiences. However, as clinical exposure is variable, the didactic portion introduced basic concepts of the code status. Specifically, the didactic portion introduced different settings in which to discuss code status, basing discussions on trajectories of illness and risk of death, reviewing frameworks that can be used to discuss code status in a variety of prognoses, and identifying the success rate of cardiopulmonary resuscitation. Additionally, we stressed key differences applicable to pediatrics, surgery, and OB/GYN when discussing code status. The didactic portion ended with the presenters modeling a case in which the framework of discussing code status with an SP was implemented, presenting the case of a patient with an advanced chronic illness.

For each clinical case of the three total cases (Appendix B), we asked one student to play the role of an intern, while the SP would play the role of either the patient or a family member. Those students who did not interact with the SP observed their peers' interaction and were asked to take note of what went well and what seemed challenging. Groups had about 15 minutes to work through each case, during which time we encouraged more than one student to practice. Next, we performed a large-group debrief to discuss lessons learned and learning points for each case. The SPs rotated groups before proceeding to the next case to add to the realistic portrayal of the scenarios. This process ran for three cases, after which we allowed about 15 minutes for questions and final conclusions.

Though cases were written based on the students' self-selected tracks, the cases for each workshop progressed from lower to higher difficulty and are summarized as follows:

Case 1 involves a clinically stable patient admitted for an acute disease process. The student is tasked to discuss code status for an admitted in-patient and to explain the term code status in a clear, nonthreatening manner.

Case 2 simulates a patient who has an advanced chronic illness for which there are no further curative treatment options. This case allows the student to explore the patient's values and encourages them to make a recommendation to the patient.

Case 3 introduces a critically ill patient who has had a complex course with no anticipated recovery. The student is tasked with discussing code status with the patient/guardian/next-of-kin, who requests that everything be done.

Workshop Delivery

This workshop was delivered in a large classroom or auditorium with a computer and audiovisual equipment allowing projection of a PowerPoint presentation. We recommend a ratio of three students per SP, and the room should be of sufficient size to allow the small groups of students with their SP to congregate at least 10 feet away from other groups. This workshop can be successfully delivered by one facilitator; if more than one facilitator is present, facilitators can perform direct observation of groups while they work through the cases with the SPs and provide real-time feedback.

As discussed in Appendix C, the overall workshop runs over 1 hour and 45 minutes, including 20 minutes allocated for an introductory didactic session, 15 minutes for addressing each of the three cases, with 5-minute debriefs after each one, a 5-minute break midway through the workshop, 5 minutes for students to write questions on index cards, and 15 minutes for facilitators to respond to questions and wrap up the workshop. After the workshop, two handouts (Appendix D) were posted to an online platform available to students, which they could use to review the frameworks practiced in this workshop. One handout reviewed CSDs for those with advanced illness while the other handout pertained to CSDs in those without advanced illness. To encourage students to develop their own words rather than rely on a templated handout, the handouts were not provided during the workshop but were made available afterward as a resource for continued training.

Faculty Preparation

To prepare faculty facilitators across two regional campuses and one regional site, we set up a 1-hour virtual meeting during which we reviewed the Facilitator Guide (Appendix C), which includes the workshop timeline, cases, SP instructions for each case, and debriefing points. Additionally, we reviewed the introductory didactic PowerPoint which outlined the learning objectives for the workshop. Faculty had opportunities to ask questions and review talking points. The student and facilitator handouts were shared with the lead faculty at each campus/site in advance of the training session. This virtual session was also recorded and made available to faculty unable to attend the live 1-hour meeting.

Learner Assessment

We evaluated student feedback with a pre- and postworkshop paper survey (Appendix E), which included five questions assessing student confidence, with answers rated on a 5-point Likert scale. We created the surveys by adapting a previously published medical education communication curriculum's learner survey.[21] Upon arrival at the workshop, each student was briefed on the surveys and then handed two identical copies of it. The surveys in each packet were labeled with an identical number

Copyright © 2025 Bechtold et al. This is an open-access publication distributed under the terms of the Creative Commons Attribution license.

followed by either A or B to deidentify survey respondents while also allowing pre- and postworkshop surveys to be matched. Students were given 5 minutes to complete the first survey, then the completed copy was collected. At the workshop's conclusion, students completed the second survey prior to leaving the classroom.

SP Training

SPs were recruited based on skill and availability from among the employed SPs through the University of Kentucky's Standardized Patient Program. Participating SPs attended a 60-minute training session via synchronous videoconference, led by the lead faculty. During the training, the faculty briefly reviewed the learning objectives and workshop agenda; most of the time was spent dissecting the three clinical cases to help guide the SPs about appropriate emotional responses. They were provided with the workshop SP guide (Appendix F).

Results

A total of 121 medical students completed the workshop at the main campus, where we distributed in-workshop surveys. A pre- or postworkshop survey was completed by 84% of the students ($n = 102$); 69% ($n = 84$) completed both. We used asymptotic McNemar's tests to assess the relationship between students' answers before and after the workshop, accounting for the paired nature of the data. Only students who completed both surveys were included in the data analysis. Data were analyzed using R (R Core Team, 2023). A *p* value of .05 was used as the cutoff for statistical significance.

Overall, we found an increase in student confidence levels in conducting CSDs, as measured on a 5-point Likert scale (1 = *not confident at all*, 5 = *extremely confident*) in response to all five survey questions. The Figure illustrates the overall proportions of students with confidence responses from before the workshop versus after the workshop.

Table 1 compares confidence levels between pre- and postworkshop responses to the five questions, with significance of change measured by McNemar's test, for all students combined ($n = 84$) and for each cohort stratified by separate track. Among all respondents, a statistically significant trend toward higher confidence was seen following the workshop. There was no statistically significant differences when comparing pre- and postworkshop responses in the individual cohorts; the smaller number of participants in these individual cohorts may explain the nonsignificant *p* values.

For a numeric and more simplified summary of change in confidence following the workshop, Table 2 displays the mean confidence levels from both before and after the workshop for those students who completed both the pre- and postworkshop surveys. For these summaries, no statistical analyses were performed given the preference for McNemar's tests, which are specifically designed to account for the paired pre- and postworkshop categorical responses.

Discussion

To prepare medical students to lead CSDs in residency, we created a 1-day workshop that offers structured, low-stakes

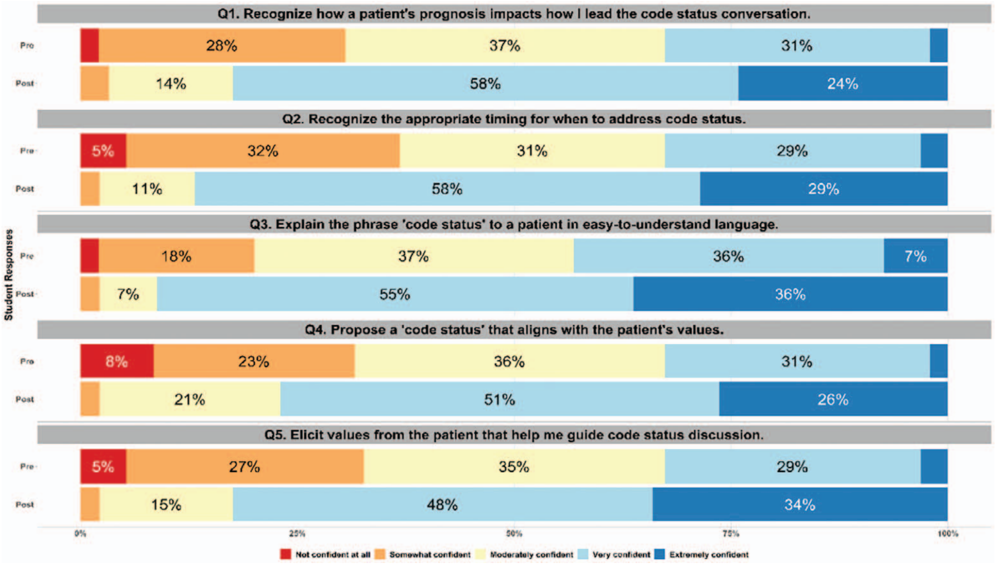

Figure. Overall response proportions (pre- and postworkshop) on five survey questions regarding student confidence levels.

Copyright © 2025 Bechtold et al. This is an open-access publication distributed under the terms of the Creative Commons Attribution license.

Table 1. Summary *p* Values for McNemar's Tests of the Changes Between Pre- and Postworkshop Confidence by Overall Responses and Individual Track Cohort Responses

Statement	Overall ($N = 84$)	Surgery ($n = 27$)	Internal Medicine ($n = 28$)	Med-Peds/Family Medicine ($n = 16$)	OB/GYN ($n = 4$)	Pediatrics ($n = 9$)
Q1. Recognize how a patient's prognosis impacts how I lead the code status conversation.	<.001	.02	.001	.01	.04	.12
Q2. Recognize the appropriate timing for when to address code status.	<.001	.005	<.001	.09	.14	.07
Q3. Explain the phrase code status to a patient in easy-to-understand language.	<.001	.06	<.001	.02	.26	.06
Q4. Propose a code status that aligns with the patient's values.	<.001	.08	.001	.05	.14	.04
Q5. Elicit values from the patient that help me guide code status discussion.	<.001	.12	<.001	.002	.22	.14

Abbreviations: Med-Peds, medicine-pediatrics.

practice with SPs. The workshop is delivered just before graduation during the TTR course, in accordance with the just-in-time approach used for other essential intern-level skills. Our workshop focuses on relatable, commonly encountered clinical scenarios and includes cases framed through multiple specialty lenses. Thus, our workshop fills a well-documented educational gap, one that affects both the learners and the vulnerable patients they serve.

Our results indicate a statistically significant improvement in student confidence across each of the five domains: recognizing how patient prognosis impacts the framing of the CSD; recognizing when to address code status; explaining the phrase code status to a patient; eliciting patient values that guide the conversation; and proposing a code status that aligns with the patient's values. The improvement in each of these domains suggests achievement of Kirkpatrick Level 1 for each, especially as most students chose responses of *very confident* or *extremely confident* in each domain following the workshop. Notably, compared to student responses regarding the other domains, more students initially lacked confidence in the domain of proposing a code status that aligns with a patient's values. Clinicians must have confidence in their assessment of a patient's health and appreciation of a patient's values when helping a patient decide on code status. It seems fitting that this workshop boosted learner confidence in this task, given the opportunity to practice such scenarios; indeed, our results show that all students indicated feeling at least *somewhat confident* in this domain after the workshop, with the majority feeling *very confident* to *extremely confident*. Other institutions may wish to create their own OSCE or other validated instrument to test whether Kirkpatrick Level 2 was achieved. We believe the integration of SPs was a critical component of our success, offering both external motivation to learners and a realistic environment in which learners could safely practice this challenging skill.

Table 2. Student Mean Confidence Levels Pre- and Postworkshop in Paired Samples ($N = 84$)

Question	Pre *M*	Post *M*	*M* Increase
Q1. Recognize how a patient's prognosis impacts how I lead the code status conversation.	2.99	4.01	1.02
Q2. Recognize the appropriate timing for when to address code status.	2.94	4.12	1.18
Q3. Explain the phrase code status to a patient in easy-to-understand language.	3.25	4.23	0.98
Q4. Propose a code status that aligns with the patient's values.	2.96	4.0	1.04
Q5. Elicit values from the patient that help me guide code status discussion.	2.95	4.12	1.17

Learner confidence in high-stakes communication, including CSDs and delivering serious news, is critically important, as it can be protective against in-the-moment stress responses and may reduce burnout.[1,15] Conducting CSDs is a skill built over time through repetition and coaching. Our findings suggest that this workshop helps learners enter residency with greater confidence in CSDs. Just-in-time training in advanced communication skills like CSDs has the potential to increase learner confidence and promote physician well-being during the transition from medical student to resident.

While several CSD trainings have been published, our workshop is notable for its larger number of participants and strategic timing during the final weeks of medical school. Many existing curricula report sample sizes of fewer than 30 learners.[9,11,14] In contrast, we included 121 participants from the main campus, with 84 students completing both pre- and postworkshop surveys, strengthening the reliability of our results. Although the sizes of each individual cohort limited statistical analysis, most of our cohort sizes were comparable to those reported in the literature. Of the two publications with larger sample sizes (N ranging from 106 to 135 participants), one included

Copyright © 2025 Bechtold et al. This is an open-access publication distributed under the terms of the Creative Commons Attribution license.

second-year medical students and the other included fourth-year medical students participating in a required critical care clerkship starting in August of their final year.[10,12] Our just-in-time workshop, delivered just before graduation, offers a uniquely short interval between training and application, which may increase skill retention. Consequently, workshop participants may be better equipped to conduct CSDs with more clarity, specificity, and confidence, making recommendations that align with each patient's values.[18,19]

A key strength of our curriculum is its generalizability, supported by the inclusion of specialty-specific case scenarios. Potential applications may include specialty-specific intern boot camps, TTR courses, intern orientations, and resident training programs. Engagement among senior medical students increases when they practice and debrief patient cases aligned with their intended specialty, making the track-based design especially relevant for fourth-year students. The workshop's clear, organized layout is another strength. Given the nuance and emotional weight of CSDs, this workshop provides an organized framework tailored to interns' appropriate developmental needs. The didactic introduction sets the stage for this framework, and subsequently, the cases progress in required skill by acuity and simulated patient/family tension. The opportunity to practice these discussions in several different simulated settings (i.e., stable patient versus critically ill patient) provides learners with a scaffold to use when considering the role of CSDs in patient care.

Our workshop and its evaluation had several limitations. First, we were unable to directly measure the long-term impact of our workshop, as we did not follow learners into residency. Moreover, our survey was not independently validated prior to implementation. While the sample size at our main campus was robust, the regional cohorts were relatively small. Future analyses would ideally incorporate results from all locations, not just the main campus. Ideally, CSDs occur in a quiet, private environment. If space and time permit, facilitators could have the small groups practice in small, private breakout rooms to better simulate these ideal conditions. Due to classroom scheduling constraints, this was not possible in our implementation. Another area for future work involves analyzing subgroups by specialty. Although our overall number of learners trained was relatively large, individual specialty groups (e.g., OB/GYN, pediatrics) were too small to evaluate confidence differences between groups. Lastly, future studies could examine whether this workshop yields sustained improvement in learner confidence and objective skills, potentially through follow-up assessments and pre- and postworkshop performance measurements.

Appendices

A. Didactic.pptx
B. Student Case Handouts.docx
C. Facilitator Guide.docx
D. Workshop Frameworks Handouts.docx
E. Pre- and Postworkshop Survey.docx
F. SP Guide.docx

All appendices are peer reviewed as integral parts of the Original Publication.

Hunter Bechtold, MD: Resident, Internal Medicine/Pediatrics Residency Program, Department of Medicine, University of Kentucky College of Medicine; ORCID: https://orcid.org/0000-0003-3147-6784

Reagan T. Smith: Medical Student, University of Kentucky College of Medicine

Peter Wallenhorst, MD: Assistant Professor, Department of Medicine, University of Kentucky College of Medicine and Lexington Veterans Affairs Health Care System

Kaylee Gouge, MD, MPH: Resident, Internal Medicine/Pediatrics Residency Program, Department of Medicine, University of Kentucky College of Medicine

Sarah Schaefer, MD: Assistant Professor, Division of Pediatric Palliative Care, Department of Pediatrics, University of Kentucky College of Medicine

Kelsey Karnik, PhD: Biomedical Data Scientist, Department of Biostatistics, University of Kentucky College of Public Heath

Kristen McQuerry, PhD: Associate Professor, Department of Biostatistics, University of Kentucky College of Public Heath

Kristen E. Fletcher, MD: Associate Professor, Department of Medicine, University of Kentucky College of Medicine and Lexington Veterans Affairs Health Care System

Disclosures
None to report.

Funding/Support
None to report.

Ethical Approval
The University of Kentucky Institutional Review Board reviewed this project.

References

1. Wolfe AHJ, Hinds PS, du Plessis AJ, Gordish-Dressman H, Arnold RM, Soghier L. Defining objective measures of physician stress in

Copyright © 2025 Bechtold et al. This is an open-access publication distributed under the terms of the Creative Commons Attribution license.

simulated critical communication encounters. *Crit Care Explor*. 2022;4(7):e0721. https://doi.org/10.1097/CCE.0000000000000721

2. Binder AF, Huang GC, Buss MK. Uninformed consent: do medicine residents lack the proper framework for code status discussions? *J Hosp Med*. 2016;11(2):111-116. https://doi.org/10.1002/jhm.2497
3. Tulsky JA, Chesney MA, Lo B. See one, do one, teach one? House staff experience discussing do-not-resuscitate orders. *Arch Intern Med*. 1996;156(12):1285-1289. https://doi.org/10.1001/archinte.156.12.1285
4. Chung EH, Truong T, Jooste KR, Fischer JE, Davidson BA. The implementation of communication didactics for OB/GYN residents on the disclosure of adverse perioperative events. *J Surg Educ*. 2021;78(3):942-949. https://doi.org/10.1016/j.jsurg.2020.09.001
5. Siddiqui MF, Holley JL. Residents' practices and perceptions about do not resuscitate orders and pronouncing death: an opportunity for clinical training. *Am J Hosp Palliat Care*. 2011;28(2):94-97. https://doi.org/10.1177/1049909110374599
6. Gorman TE, Ahern SP, Wiseman J, Skrobik Y. Residents' end-of-life decision making with adult hospitalized patients: a review of the literature. *Acad Med*. 2005;80(7):622-633. https://doi.org/10.1097/00001888-200507000-00004
7. Margolis B, Blinderman C, de Meritens AB, et al. Educational intervention to improve code status discussion proficiency among obstetrics and gynecology residents. *Am J Hosp Palliat Care*. 2018;35(4):724-730. https://doi.org/10.1177/1049909117733436
8. Mills LM, Rhoads C, Curtis JR. Medical student training on code status discussions: how far have we come? *J Palliat Med*. 2016;19(3):323-325. https://doi.org/10.1089/jpm.2015.0125
9. Wayne DB, Cohen ER, Singer BD, et al. Progress toward improving medical school graduates' skills via a "boot camp" curriculum. *Simul Healthc*. 2014;9(1):33-39. https://doi.org/10.1097/SIH.0000000000000001
10. Rowe KA, Ouchi K, Kennedy M, Breu A, Tolchin DW, Schwartz AW. Preparing preclinical medical students for routine code status discussions: a mixed-methods study. *J Pain Symptom Manage*. 2024;67(2):138-146. https://doi.org/10.1016/j.jpainsymman.2023.10.017
11. Mills L, Rhoads C, Curtis J. Upgrading the admission code status discussion: a curriculum for medical trainees. *MedEdPORTAL*. 2015;11:10086. https://doi.org/10.15766/mep_2374-8265.10086
12. Lorin S, Rho L, Wisnivesky JP, Nierman DM. Improving medical student intensive care unit communication skills: a novel educational initiative using standardized family members. *Crit Care Med*. 2006;34(9):2386-2391. https://doi.org/10.1097/01.CCM.0000230239.04781.BD
13. Flanagan OL, Cummings KM. Standardized patients in medical education: a review of the literature. *Cureus*. 2023;15(7):e42027. https://doi.org/10.7759/cureus.42027
14. Vettese T, Weinberger J, Thati N. "Do you want us to do everything?": teaching residents to discuss resuscitation with hospitalized patients. *MedEdPORTAL*. 2015;11:10122. https://doi.org/10.15766/mep_2374-8265.10122
15. Messerotti A, Banchelli F, Ferrari S, et al. Investigating the association between physicians self-efficacy regarding communication skills and risk of "burnout." *Health Qual Life Outcomes*. 2020;18(1):271. https://doi.org/10.1186/s12955-020-01504-y
16. Brown R, Dunn S, Byrnes K, Morris R, Heinrich P, Shaw J. Doctors' stress responses and poor communication performance in simulated bad-news consultations. *Acad Med*. 2009;84(11):1595-1602. https://doi.org/10.1097/ACM.0b013e3181baf537
17. Becker C, Knzli N, Perrig S, et al. Code status discussions in medical inpatients: results of a survey of patients and physicians. *Swiss Med Wkly*. 2020;150:w20194. https://doi.org/10.4414/smw.2020.20194
18. Cubbison C, Rai A, Reid C, et al. Effect of palliative care curriculum on serious illness conversation preparedness. *ATS Sch*. 2021;2(4):642-650. https://doi.org/10.34197/ats-scholar.2021-0019IN
19. Sharma RK, Jain N, Peswani N, Szmuilowicz E, Wayne DB, Cameron KA. Unpacking resident-led code status discussions: results from a mixed methods study. *J Gen Intern Med*. 2014;29(5):750-757. https://doi.org/10.1007/s11606-014-2791-3
20. Patocka C, Pandya A, Brennan E, et al. The impact of just-in-time simulation training for healthcare professionals on learning and performance outcomes: a systematic review. *Simul Healthc*. 2024;19(1)(suppl):S32-S40. https://doi.org/10.1097/SIH.0000000000000764
21. Gouge K, Schadler A, Fletcher KE. Anger de-escalation: a standardized patient workshop for third- and fourth-year medical students. *MedEdPORTAL*. 2025;21:11504. https://doi.org/10.15766/mep_2374-8265.11504

Received: March 11, 2025
Accepted: July 20, 2025
Published: September 2, 2025

Copyright © 2025 Bechtold et al. This is an open-access publication distributed under the terms of the Creative Commons Attribution license.
